# Cyclopenta‐Fused Polyaromatic Hydrocarbon (CP‐PAH) Radicals: Synthesis, Characterization, and Quantum Chemical Calculations

**DOI:** 10.1002/chem.202502467

**Published:** 2025-10-05

**Authors:** Ali S. Acan, Jonas O. Wenzel, Joachim Podlech

**Affiliations:** ^1^ Institute of Organic Chemistry Karlsruhe Institute of Technology (KIT) Kaiserstraße 12 76131 Karlsruhe Germany; ^2^ Institute of Inorganic Chemistry Karlsruhe Institute of Technology (KIT) Kaiserstraße 12 76131 Karlsruhe Germany

**Keywords:** aromatic compounds, aromaticity, cyclopenta‐fused polyaromatic hydrocarbons, DFT calculations, radicals

## Abstract

Two air‐ and moisture‐stable cyclopenta‐fused polyaromatic hydrocarbon (CP‐PAH) radicals with six six‐ and three five‐membered rings alternately fused to nonacycles were obtained by *ortho* fusion in suitably *ortho*,*ortho*’‐substituted dinaphthylfluorenes and subsequent establishment of the conjugation. The radicals were obtained in five consecutive steps with total yields of 38 and 16%, respectively; key steps are Suzuki couplings and cyclizing S_E_Ar reactions. Mesityl substituents at the five‐membered rings ensure the kinetic stability of the radicals. They were characterized by EPR and UV/Vis spectroscopy. Quantum chemical calculations led to simulated UV/Vis/NIR spectra and disclosed further properties like spin densities, aromaticity, and orbital energies. Both radicals are best described with the unpaired electron centered in the outer five‐membered rings. The respective resonance formulas show the largest number of fully intact benzene rings. A possible triradical character was computed to be small in both compounds. The five‐membered rings, especially the central rings show significant antiaromatic character.

## Graphical abstract

Extending the π system of a fully conjugated framework composed of alternating five‐ and six‐membered rings led to further rare examples of bench‐stable carbon‐centered hydrocarbon radicals. Spectroscopic analysis allowed extensive characterization, which was further supported by computational methods.

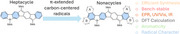

## 1 Introduction

**Figure 1:**
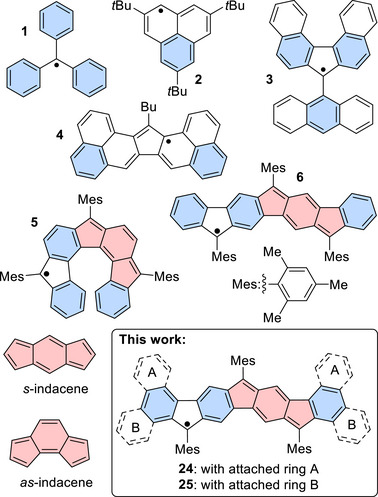
Triphenylmethyl, phenalenyl, and fluorenyl radicals and combinations thereof; linearly arranged CP‐PAH radicals, synthesized previously in our group; target structures. (Fully intact benzene rings are highlighted in blue; indacene units are shaded in red.).

π‐Conjugated polycyclic hydrocarbons (PHs),^[^ ^1^, ^2^, ^3^, ^4^, ^5^, ^6^ ^]^ in which six‐ and five‐membered rings are arranged alternately, are referred to as cyclopenta‐fused polyaromatic hydrocarbons (CP‐PAHs).^[^ ^7^, ^8^, ^9^, ^10^ ^]^ CP‐PAHs with an uneven number of five‐membered rings are inevitably radicals in their neutral and fully conjugated states (when all rings are fused over only one bond). These open shell systems are intrinsically unstable, making them challenging to study. However, since 1900, when the triphenylmethyl radical **1** was originally proposed by Gomberg,^[^ ^11^, ^12^ ^]^ there has been a growing interest in organic radicals, and several carbon‐centered hydrocarbon radicals have been reported and investigated.^[^ ^13^, ^14^, ^15^, ^16^, ^17^, ^18^, ^19^, ^20^, ^21^, ^22^, ^23^ ^]^ Actually, virtually all of these can be assorted into specific classes of radicals. These include triarylmethyl radicals, cyclopentadienyl‐^[^ ^17^, ^24^, ^25^ ^]^ and fluorenyl‐based^[^ ^26^ ^]^ structures, as well as radicals, which are part of electron push‐pull systems^[^ ^27^ ^]^ or larger π systems.^[^ ^28^, ^29^, ^30^, ^31^ ^]^ A small selection of hydrocarbon radicals belonging to different classes is given in Figure 1 and includes Yamamoto's and Nakasuji's phenalenyl radical **2**,^[^ ^15^ ^]^ a π‐extended fluorenyl radical **3**,^[^ ^19^ ^]^ and radical **4** as a combination of both the phenalenyl and fluorenyl motif.^[^ ^18^ ^]^

The synthesis of radicals with sufficient stability is a prerequisite for their further examination and application. Generally, a kinetic stabilization can be achieved by protecting the positions of highest spin densities with bulky substituents, preventing possible side reactions emerging from the radical properties, e.g., σ‐dimerization.^[^ ^32^ ^]^ However, only a small fraction of the presented carbon‐centered radicals is isolable and stable against air, moisture, solvents, and against dimerization or disproportionation. In addition to their structural properties, these radicals have a number of promising electronic, optical, and magnetic properties allowing for their application in organic electronics^[^ ^4^ ^]^ or nonlinear optics.^[^ ^33^ ^]^ Their paramagnetic properties further allow their utilization in the field of spintronics^[^ ^34^, ^35^ ^]^ or as organic magnets^[^ ^36^, ^37^, ^38^ ^]^ and could enable fully organic spintronic devices.^[^ ^39^ ^]^ Furthermore, considering that the physical properties of hydrocarbon radicals primarily depend on the arrangement of the π electrons, the design of novel hydrocarbon radicals is crucial for the development of new molecular scaffolds that promise versatile application in spintronics.^[^ ^19^ ^]^ As one of the simplest members of the conjugated hydrocarbon radicals, the cyclopentadienyl radical was hardly investigated and found limited use in applications due to its inherent high reactivity.^[^ ^17^ ^]^

Aromaticity is an important feature of fully conjugated polycyclic systems.^[^ ^40^ ^]^ The [4*n* + 2] π electron rule, established by Hückel in 1931, provides a fundamental criterion for the identification of aromatic systems.^[^ ^41^ ^]^ Dewar and Breslow later introduced the [4*n*] π electron rule to describe antiaromatic systems.^[^ ^42^, ^43^, ^44^ ^]^ These rules are easily applicable but do not account for the specifics of polycyclic systems.^[^ ^45^, ^46^ ^]^ Moreover, since aromaticity cannot be measured directly, its definition and practical relevance have been subject of ongoing discussion.^[^ ^47^ ^]^ Despite the controversy, the concepts’ undeniable utility has led to its continued use.^[^ ^48^ ^]^ In recent years computational density functional theory (DFT) methods became a powerful tool to elucidate and analyze the aromatic or antiaromatic character of investigated structures. Among possible approaches, the Nucleus‐Independent Chemical Shift XY (NICS‐XY) scan turned out to be particularly useful and allows to predict and visualize the ring current in polycyclic systems by simulating a molecule's magnetic response to an external magnetic field.^[^ ^49^ ^]^ Recently, we have succeeded in synthesizing, isolating, and characterizing two stable fluorenyl‐based radicals, i.e., a helical carbon‐centered radical **5** ^[^ ^50^ ^]^ and its isomer **6** with a roughly linearly arranged and thus planar core (Figure 1).^[^ ^51^ ^]^ Both were obtained by *ortho* fusions in suitable *ortho*,*ortho*’‐disubstituted teraryls and subsequent establishment of the π conjugation. Both synthesized structures contain the indacene core (Figure 1) along with its polycyclic fused indenofluorene moiety. In the concept of aromaticity indacene represents a particularly interesting system. Due to its twelve π electrons [4*n*], it would formally be classified as an antiaromatic species and is consequently expected to be an unstable molecule with alternating bond lengths.^[^ ^42^, ^43^ ^]^ If not part of a larger polycyclic systems, *s*‐indacene is kinetically stable only if substituted with bulky residues. In this case it can be isolated and thoroughly characterized.^[^ ^52^ ^]^ In this work, we aimed to extend the π system of such CP‐PAH radicals: This allows for an elucidation of structure‐property relationships, especially with respect to the aromatic and antiaromatic characteristics.

### 1.1 Synthesis

**Scheme 1:**
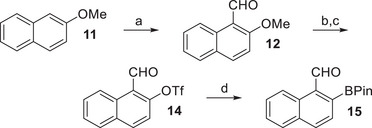
Synthesis of naphthalene‐derived boronate **15**. Conditions: a) DMF, POCl_3_, 95 °C, 5 hours (94%); b) AlCl_3_, CH_2_Cl_2_, −5 °C to rt, 4 hours (**13**, 76%); c) DMAP, Tf_2_O, CH_2_Cl_2_, 0 °C to rt, 5 hours (61%); d) B_2_Pin_2_, PdCl_2_(dppf), KOAc, 1,4‐dioxane, 80 °C, 48 hours (quant.).

**Scheme 2:**
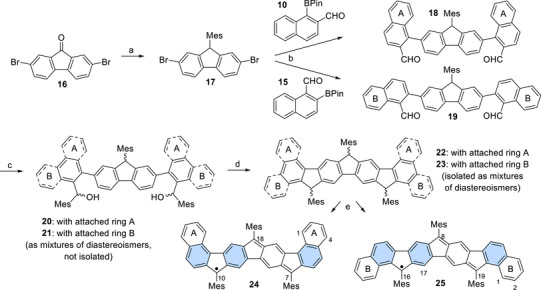
Synthesis of nonacyclic radicals **24** and **25**. Conditions: a) 1. FeCl_3_, Me_3_SiCl, Et_3_SiH, mesitylene, rt, 2 hours; 2. 50 °C, 18 hours (74%); b) **10** or **15**, Cs_2_CO_3_, PdCl_2_(dppf), toluene/H_2_O (1:1), 105 °C, 18 hours [67% (**18**); 44% (**19**)]; c) MesMgBr, THF, 0 °C to rt, 15 minutes (not isolated); d) BF_3_·OEt_2_, CH_2_Cl_2_, 0 °C to rt, 1 hours [79% (**22**); 78% (**23**)]; e) *t*BuOK, THF, 60 °C, 18 hours, then *p*‐chloranil, rt, 10 minutes [96% (**24**); 64% (**25**)].

Synthesis of radicals **24** and **25** was initiated by preparation of the corresponding boronates **10** and **15** (Scheme 1), followed by Suzuki coupling with the respective dibromofluorene building block **17**, and finalized by an *ortho* cyclization with subsequent rearomatization (Scheme 2). The synthesis of boronate **10** was already reported in the literature and was achieved accordingly in a total yield of 58% over three consecutive steps (compounds **7**, **8**, and **9** are shown in the SI).^[^ ^53^, ^54^ ^]^ Synthesis of isomeric boronate **15** was achieved by an initial Vilsmeyer formylation of commercially available 2‐methoxynaphthalene (**11**).^[^ ^55^ ^]^ Demethylation of **12** furnished 2‐hydroxynaphthalene‐1‐carbaldehyde (**13**),^[^ ^56^ ^]^ which was transformed into the corresponding triflate **14** ^[^ ^57^ ^]^ and again borylated in a Miyaura reaction to yield boronate **15** in a total yield of 43% over four consecutive steps.

**Figure 2:**
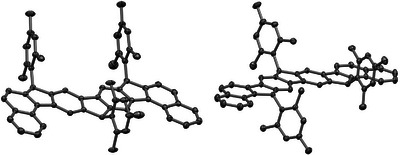
Molecular solid‐state structures of all‐*cis*‐**22** (left) and *cis*,*trans*‐**23** (right) obtained by single crystal X‐ray diffraction.^[^ ^60^ ^]^ Hydrogen atoms and co‐crystallized toluene molecules are not shown for clarity. Thermal ellipsoids are drawn at the 30% probability level. Enlarged figures are given in the SI.

Transforming commercially available 2,7‐dibromo‐fluoren‐9‐one (**16**) into the respective mesityl‐substituted fluorene **17** was achieved by Lewis acid‐mediated reduction/electrophilic aromatic substitution (Scheme 2).^[^ ^51^, ^58^ ^]^ Double Suzuki coupling of **17** with boronates **10** or **15**, respectively, furnished dicarbaldehydes **18** or **19**, which were subjected to nucleophilic additions with mesityl Grignard reagents. Thus accessible dialcohols **20** and **21** were cyclized with boron trifluoride etherate.^[^ ^59^ ^]^ Nonacycles **22** and **23** were obtained as mixtures of diastereoisomers whose separation was neither possible nor necessary for their subsequent reaction. Treatment with potassium *tert*‐butanolate and immediate oxidation with tetrachloro‐*p*‐benzoquinone (chloranil) yielded fully conjugated isolable radicals **24** and **25**.^[^ ^20^ ^]^ Radical **24** was thus obtained in eight consecutive steps from **7** in a total yield of 22%; radical **25** required nine consecutive steps from **11**, for which a total yield of 7% was achieved. Both compounds turned out to be stable against air, moisture, daylight, and dimerization. Melting points of 432 °C (radical **24**) and 422 °C (radical **25**) were determined by differential scanning calorimetry with integrated thermogravimetric analysis (DSC/TGA). Further transitions were not observed below the radicals’ decomposition temperatures at 477 °C and 481 °C, respectively (see SI). We previously reported that numerous attempts to obtain single crystals of radical **6** suitable for X‐ray crystallographic analysis were unsuccessful due to dendritic crystal formation, preventing a crystallographic structure elucidation.^[^ ^51^ ^]^ Similar issues were observed for radicals **24** and **25**. However, we were successful to crystallize the corresponding precursors **22** and **23** by slow cooling in toluene and obtained colorless crystals. The respective X‐ray crystallographic analyses not only confirmed the constitutional arrangement of the polycyclic scaffolds of **22** and **23**, but were furthermore an implicit proof for the constitution of radicals **24** and **25**, since the finalizing steps do not lead to any scaffold modification.^[^ ^50^ ^]^ It turned out that we randomly picked a crystal of **22** in its all‐*cis* (*meso*) configuration and of **23** in the chiral *cis*,*trans* configuration (Figure 2) where the latter crystallized as a racemate.^[^ ^60^ ^]^ **22** crystallized in a triclinic and **23** in a monoclinic crystal system. This supports our assumption that more than one diastereomer was formed during the respective cyclizations.^[^ ^51^ ^]^

### 1.2 Spectroscopic and Quantum‐Chemical Investigations

**Figure 3:**
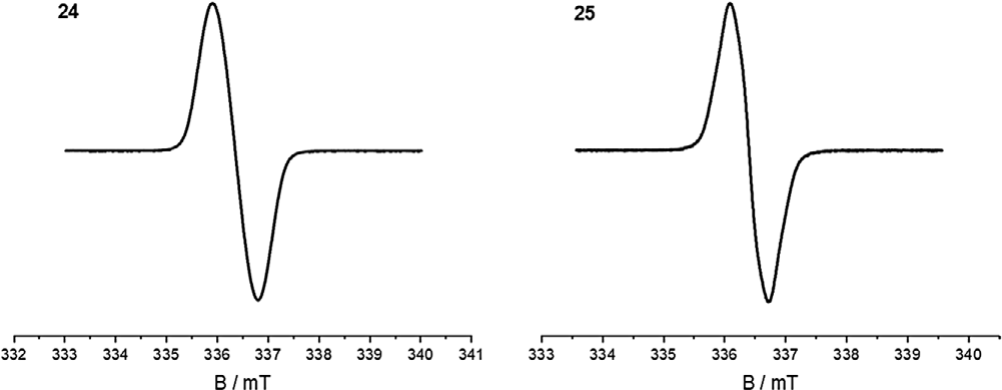
Cw X‐band [9.4280 GHz (**24**); 9.4279 GHz (**25**)] EPR spectra of radicals **24** and **25** in toluene (0.1 mM) at ambient temperature showing a single resonance signal centered at *g* = 2.0027 (**24**) and *g* = 2.0024 (**25**).

Evidence for the radical character of **24** and **25** was provided by the absence of any resonances in standard frequency ranges of their respective nuclear magnetic resonance (NMR) spectra and by their electron paramagnetic resonance (EPR) activity (Figure 3). Measured X‐band EPR spectra for radicals **24** and **25** show a single line centered around *g* = 2.0027 (**24**) and *g* = 2.0024 (**25**) without any discernible hyperfine couplings.

**Figure 4:**
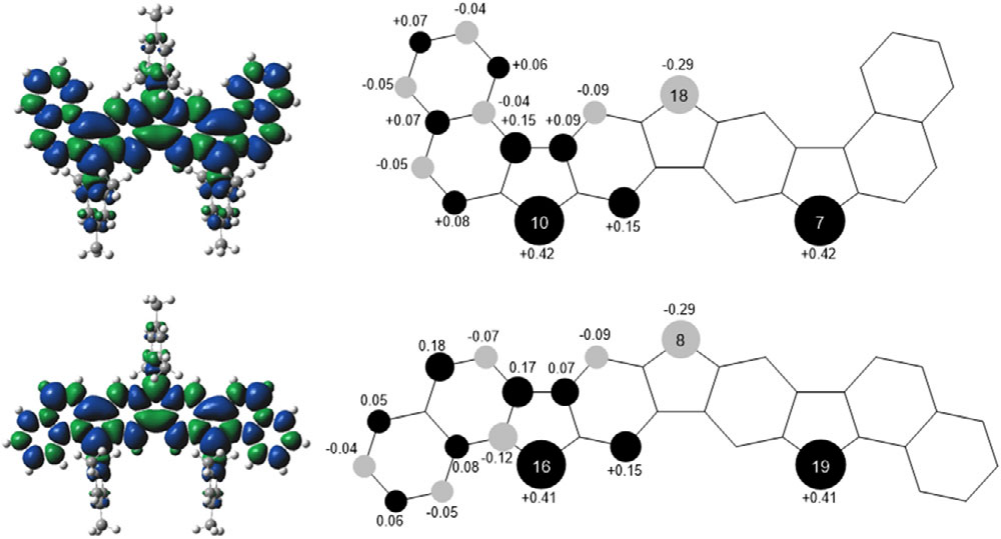
Calculated spin densities of radicals **24** (top) and **25** (bottom); blue: α spin, green: β spin (isovalue: 0.004 electrons·bohr^‒3^; calculated at the upbe0/def2‐TZVP/GD3BJ level).

Calculated EPR data indicate a strong delocalization of spin densities over all carbon atoms of the nonacyclic cores, which are thus relatively small at the respective positions. This explains the unresolved hyperfine structure in the measured spectra, which is further supported by calculated small coupling constants (see SI). Distinct Mulliken atomic spin densities (Figure 4) are nevertheless calculated for those carbon atoms, which are not part of a benzene ring (i.e., at the mesityl‐substituted positions), where densities at the respective outer carbons (C‐7, C‐10 in radical **24** and C‐16, C‐19 in **25**) turned out to be highest (0.42 and 0.41, respectively) and those at the central positions (C‐18 in **24**, C‐8 in **25**) are slightly smaller (−0.29 for both radicals).

**Figure 5:**
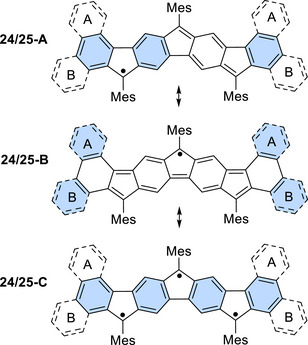
Resonance formulas of radicals **24** (ring fusion A) and **25** (B). Fully intact benzene rings are highlighted.

The corresponding equivalent resonance formulas **24‐A** and **25‐A** and their (non‐depicted) mirror images (Figure 5) show three fully intact benzene rings and thus should be most relevant in the respective resonance ensembles according to Clar's rule.^[^ ^61^ ^]^ In resonance formulas **24‐B** and **25‐B**, where the unpaired electron is located at the central five‐membered ring, only two fully intact benzene units are recognized at the termini of the nonacyclic core; these represent less significant resonance formulas among the possible structures. Formulas **24‐C** and **25‐C** would reflect a possible triradical character and show four intact benzene units. Calculation of the natural orbital occupation numbers (NOONs)^[^ ^62^ ^]^ and application of the Yamaguchi scheme led to *y* values of 0.36 and 0.39, respectively, indicating only a minor degree of triradical character. Consequently, resonance structures **24‐A**/**25‐A** and their mirror images should have the highest relevance in the respective resonance ensembles, which is in agreement with prior studies of our group.^[^ ^50^, ^51^ ^]^

**Figure 6:**
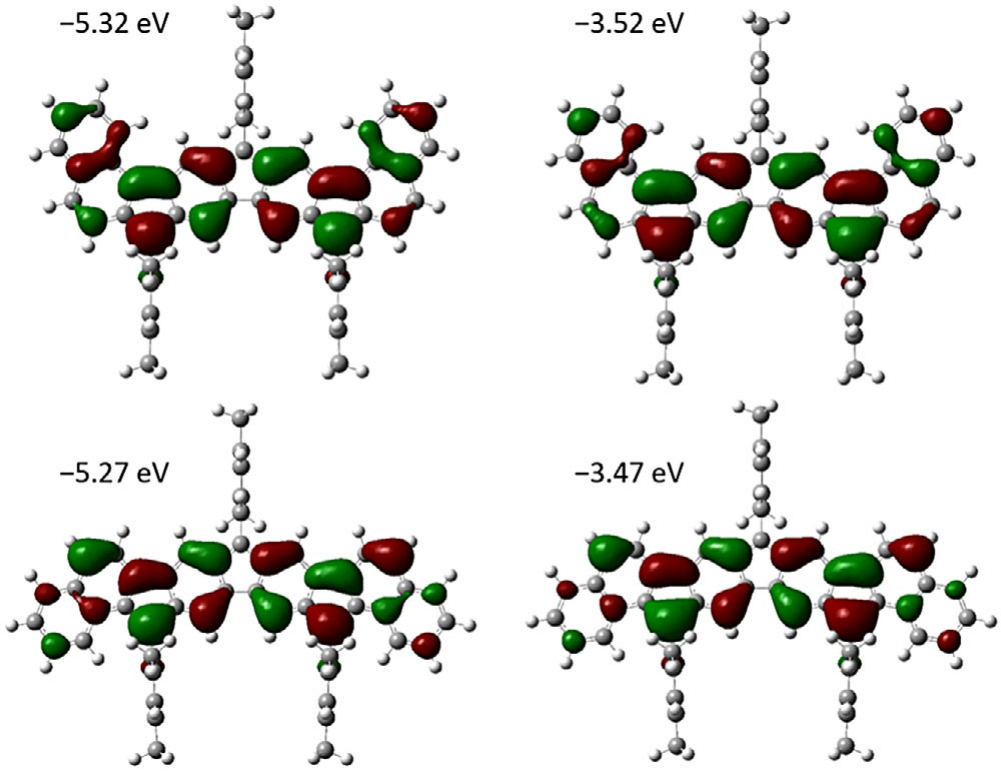
Calculated SOMOs of α and β electrons for radicals **24** (top) and **25** (bottom) (also termed SOMO and SUMO in the literature;^[^ ^63^ ^]^ isovalue: 0.02 electrons^1/2^ bohr^−3/2^; calculated at the upbe0/def2‐TZVP/GD3BJ level).

SOMOs of radicals **24** (top) and **25** (bottom) (also referred to as SOMO and SUMO in the literature;^[^ ^63^ ^]^ Figure 6) show nodal planes through the centers of the molecules; the negative spin density values at C‐8 and C‐18 could therefore be due to negative spin polarization. The energies of the SOMOs are −5.32 and −3.52 eV for radical **24** and −5.27/−3.47 eV (radical **25**) for the α and β spin, respectively.

**Figure 7:**
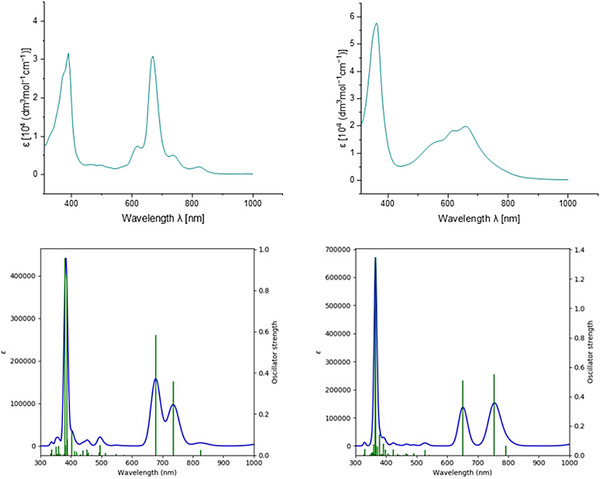
UV/Vis spectra of radical **24** (left) and **25** (right) measured in CH_2_Cl_2_ (top) and calculated (bottom).

UV/Vis spectra of radicals **24** and **25** were calculated in time‐dependent (TD) DFT calculations [upbe0/def2tzvp/GD3BJ level] with a simulated solvent field of methylene chloride (Figure 7, bottom; enlarged and expanded versions are given in the SI). Both spectra are quite close to their corresponding measured spectra in the same solvent (top). The calculated data for radical **24** indicate a vanishingly small calculated absorption at 1201 nm (1.03 eV, oscillator strength *f* = 0.0002) which is mainly due to a SOMO → LUMO (94%) transition of the β spin. A weak absorption at 825 nm, which is similarly visible in the measured spectrum, (1.50 eV, *f* = 0.027) can be attributed to the HOMO – 1 → LUMO transition of the α spin and the SOMO → L + 1 transition of the β spin. Pronounced absorptions at 736 nm (1.68 eV, *f* = 0.36), 678 nm (1.83 eV, *f* = 0.58), and 403 nm (3.08 eV, *f* = 0.12) can essentially be ascribed to transitions of the α spin (SOMO → LUMO, 42%; H – 1 → LUMO, 19%; and SOMO → L + 2, 27%) and of the β spin (H – 1 → LUMO, 19%; SOMO → L + 1, 18%; H – 1 → L + 2, 17%). A similar trend is observed for radical **25**: A barely existing calculated absorption at 1107 nm (1.12 eV, *f* = 0.0000) is mainly due to SOMO → LUMO transition (94%) of the β spin. A weak absorption at 792 nm (1.56 eV, *f* = 0.06) induced by H – 1 → LUMO transition (61%) of the α spin and SOMO → L + 1 transition (23%) of the β spin is hardly visible in the measured spectra due to the broad absorption in this region. Again, a pronounced absorption at 754 nm (1.64 eV, *f* = 0.55) can be assigned to SOMO → LUMO transition (94%) of the α spin and H – 1 → LUMO transition (16%) of the β spin. There is hardly any visible contribution of the mesityl groups in these low energy transitions; only π orbitals of the nonacyclic core are involved here. Further transitions are indicated in the SI. Low‐energy electronic transitions of **24** and **25** are thus significantly red‐shifted as compared to those of the heptacyclic radicals **5** and **6**. Respective transitions of helical radical **5** were calculated to be 1.17 (1059 nm, *f* = 0.002) and 1.53 eV (810 nm, *f* = 0.17)^[^ ^50^ ^]^ and linear radical **6** showed negligible transitions at 1.34 (925 nm, *f* = 0.0) and 1.42 eV (873 nm, *f* = 0.03) and a first significant transition at 1.86 eV (666 nm, *f* = 0.57).^[^ ^51^ ^]^

No fluorescence activity was observed for radicals **24** and **25**. It has been reported that indenofluorenes show extremely short excitation lifetimes, possibly due to conical intersection.^[^ ^64^ ^]^ This behavior is in agreement to prior findings by our group.^[^ ^50^, ^51^ ^]^

The electrochemical characterization of **24** and **25** by cyclic voltammetry (CV) turned out to be unsuccessful, since the acquisition of evaluable CV data for compounds **24** and **25** was not possible. A somewhat more detailed discussion is given in the SI together with the corresponding CV plots.

**Figure 8:**
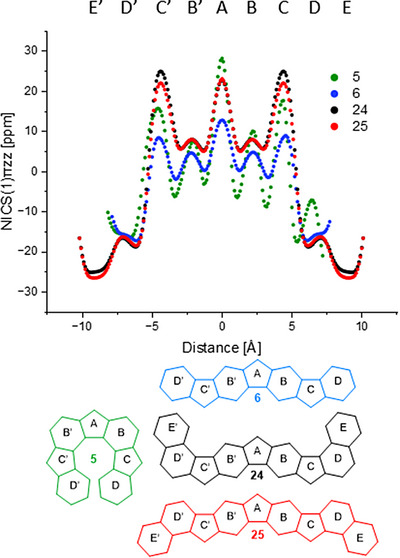
NICS(1)_πzz_‐XY‐scans of radicals **5**, **6**, **24**, and **25**.

Aromaticity, i.e., an aromatic or antiaromatic character, is a key feature of a fully conjugated cyclic or polycyclic system as it is strongly related with its optoelectronic properties. It can be estimated for a compound as a whole or separately for each of the rings in a polycyclic system.^[^ ^40^ ^]^ Among further methods, calculations of NICS (Nucleus‐Independent Chemical Shift) values utilize the response of an aromatic system to an external magnetic field. These are usually determined at 1 Å distance from the center of each ring [NICS_zz_(1.0) values]^[^ ^49^, ^65^, ^66^, ^67^, ^68^ ^]^ and are used to quantify a compound's aromatic character in the respective ring: Negative values indicate an aromatic ring, while antiaromatic systems give rise to positive numbers. NICS_zz_(1) values of −21.6, −14.5, +22.2, +10.2, and +16.0, respectively, were calculated for rings E to A for radical **24** and values of −23.1, −13.4, +20.0, +10.2, and +23.8, respectively, for radical **25**. Rings E, E’, D, and D’ show aromaticity close to that of a single benzene ring, while rings C and C’ and especially A turned out to be antiaromatic. Rings B and B’ show a borderline behavior, they are neither clearly aromatic nor antiaromatic. NICS‐XY‐scans offer further insight into a polycycle's aromaticity pattern: These are constructed by calculation of NICS values (typically again at 1 Å distance to the ring system) along a path through the core of the compound passing all ring centers. This allows for a better estimation of the aromatic character in polyaromatic systems with condensed rings showing more than one ring current.^[^ ^69^ ^]^ The calculated scans (Figure 8) show local maxima with values that are virtually identical to those obtained from NICS_zz_(1) calculations.

**Figure 9:**
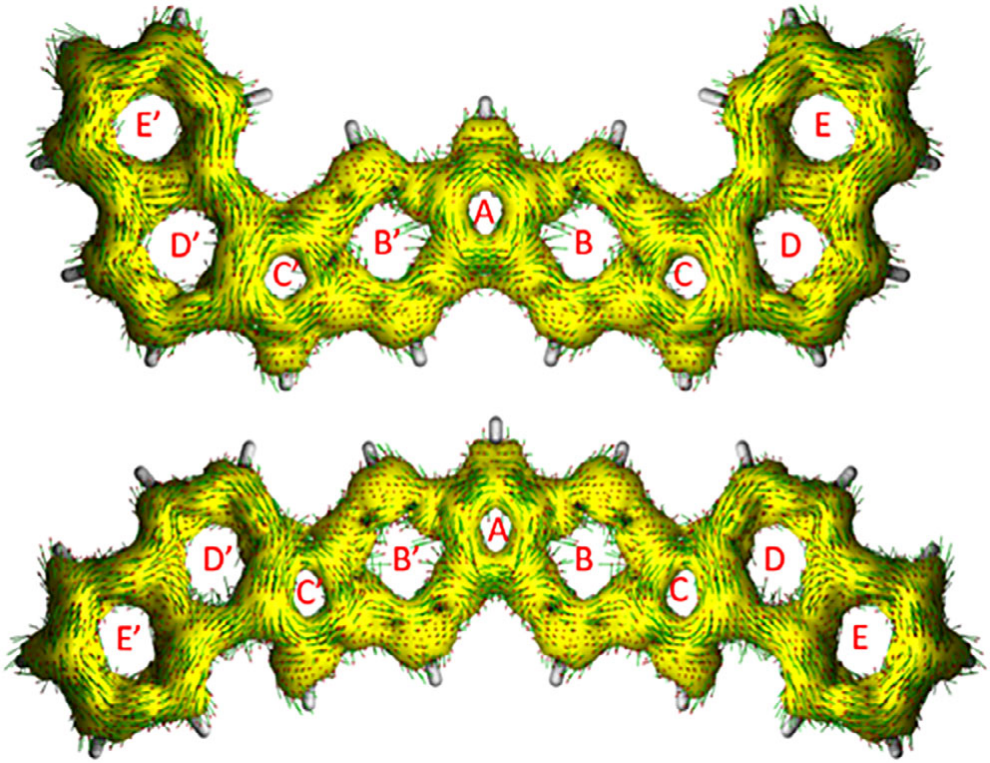
ACID isosurface plot of radicals **24** (top) and **25** (bottom) (calculated without mesityl groups; isosurface values: 0.025).

A last method used herein for a quantification of aromaticity is based on calculated induced ring currents. The anisotropy of induced current densities was calculated and visualized using Herges’ ACID method (Figure 9).^[^ ^70^, ^71^ ^]^ Rings E, E’, D, and D’ show diatropic (clockwise in this picture) ring currents again indicating an aromatic character, which is in agreement with the obtained NICS values. Rings C, C’, and A, however, show paratropic (anticlockwise) ring currents, indicating their antiaromatic character. A rather unsteady behavior is recognized for rings B and Bʼ: Paratropic as well as diatropic ring currents are present, which is again in agreement with the obtained NICS values. (An enlarged picture of the figure is given in the SI.) The respective calculations were performed with the parent polycycles without mesityl groups to obviate their influence on the ring currents.

Comparison of the NICS‐XY‐scans (Figure 8) of radicals **5**, **6**, **24**, and **25** reveals significant differences in the NICS values. Comparison of the plots of compounds **5** (green) and **6** (blue) shows good agreement for the outer six‐membered rings D and D’, but remarkable differences for all other rings. A more pronounced antiaromatic behavior of **5** is not only observed in the outer five‐membered rings C, C’ and even more significant in the central ring A, but also in the inner six‐membered rings B and B’. In compound **6** (blue) the inner six‐membered rings are closer to the borderline between aromatic and antiaromatic character. This rather unusual behavior (six‐membered rings, i.e., benzene rings usually show distinctive aromatic character) might be explained by the fact that these six‐membered rings are part of an indacene unit within the heptacyclic scaffold (c.f., Figure 1). As previously mentioned, indacenes are compounds with significant antiaromatic character. Assuming the six‐membered rings are part of the indacene unit, they do not exhibit an untypical behavior, but rather reflect the paratropic ring current defined for indacene. A comparison of **24** (black) and **25** (red) reveals no substantial differences. However, extending the outer π system of the linearly aligned heptacyclic scaffold reveals remarkable changes in the NICS‐XY‐scans, especially in the five‐membered rings, which were also reported for indenofluorene derivatives.^[^ ^72^ ^]^ Comparing scans of **6** (blue) with **24** (black) shows a two‐fold increase of the antiaromatic character for ring A (from 12.7 ppm for radical **6** to 23.0 ppm for **24**) and a three‐fold increase for rings C and C’ (from 8.3 ppm to 24.9 ppm). For six‐membered rings B and B’ only a small increase (4.5 ppm to 7.5 ppm) is observed, whereas virtually identical values are calculated for rings D and D’. However, rings E and E’, which are only present in **24** (25.0 ppm) and **25** (26.5 ppm), exhibit strong aromatic behavior. The obtained data from the NICS‐XY‐scans thus indicate strong antiaromatic behavior for the majority of the scaffold. This is further supported by calculated low SOMO/LUMO gaps (**5**: 2.36 eV; **6**: 2.48 eV; **24**: 2.37 eV; **25**: 2.33 eV), which is typical for antiaromatic compounds.^[^ ^73^ ^]^ Despite their strong antiaromatic character, all compounds indicate high stability, again challenging the conventional definitions of aromaticity and antiaromaticity.^[^ ^52^, ^74^, ^75^ ^]^

## 2 Conclusion

In summary, we synthesized two new CP‐PAH radicals consisting of nine fused rings by extending the π system of a CP‐PAH radical previously reported from our group. Both structures were obtained by cyclization of an *ortho*,*ortho*’‐substituted dinaphthylfluorene and reestablishment of the conjugation. They are best described by their monoradical state and exhibit at most a small triradical character. Comparison of these radicals with already reported structures provided insights into their structure‐property relationships, especially with respect to their aromatic and antiaromatic properties.

## Supporting Information

Experimental procedures, NMR and further spectra for new compounds, crystallographic data for compounds **22** and **23**, details of DFT calculations, and geometries for all calculated structures are included in the Supporting Information. The authors have cited additional references within the Supporting Information.^[^ ^76^, ^77^, ^78^, ^79^, ^80^, ^81^, ^82^, ^83^, ^84^, ^85^, ^86^, ^87^, ^88^, ^89^, ^90^, ^91^, ^92^, ^93^, ^94^, ^95^, ^96^, ^97^, ^98^, ^99^, ^100^, ^101^, ^102^, ^103^, ^104^, ^105^, ^106^, ^107^, ^108^, ^109^, ^110^, ^111^, ^112^, ^113^, ^114^, ^115^, ^116^, ^117^, ^118^, ^119^, ^120^, ^121^ ^]^

## Conflict of Interest

The authors declare no conflict of interest.

## Supporting information



Supporting Information

Supporting Information

## Data Availability

The data that support the findings of this study are available in the supplementary material of this article.
