# Supplementary material for: Cyclopenta‐Fused Polyaromatic Hydrocarbon (CP‐PAH) Radicals: Synthesis, Characterization, and Quantum Chemical Calculations
Source: Chemistry. 2025 Oct 5;31(61):e02467. doi: 10.1002/chem.202502467 (PMC12587023; doi:10.1002/chem.202502467)
Supplement: Supplementary file 2 — Supporting Information [file CHEM-31-e02467-s001.pdf]

Cyclopenta-Fused Polyaromatic Hydrocarbon (CP-PAH) Radicals: Synthesis, Characterization, and Quantum Chemical Calculations

Ali S. Acan,[a] Jonas O. Wenzel,[b] and Joachim Podlech*[a]

[a] M.Sc. A. S. Acan, Prof. Dr. J. Podlech
Institute of Organic Chemistry
Karlsruhe Institute of Technology (KIT)
Kaiserstraße 12, 76131 Karlsruhe, Germany
E-mail: joachim.podlech@kit.edu

[b] Dr. J. O. Wenzel
Institute of Inorganic Chemistry
Karlsruhe Institute of Technology (KIT)
Kaiserstraße 12, 76131 Karlsruhe, Germany

Content:

1. Experimental Procedures
2. ^{1}H and ^{13}C NMR Spectra of all Compounds, Mass and IR Spectra
3. DSC/TGA curves
4. UV/Vis Spectra
5. EPR Spectra
6. Cyclic Voltammograms of 24 and 25
7. XRD Data of Precursors 22 and 23
8. Computational Details
9. Archive Entries for Single-Point Energy Calculations on a Minimum Structure
10. Frequency Analyses and Visualized IR Spectra
11. SOMOs of α and β Electrons
12. Calculated Spin Densities
13. TD Calculations
14. Calculated UV/Vis/NIR Spectra
15. Calculated EPR Data
16. Triradical Character
17. NICS Values
18. NICS-XY-Scans
19. ACID Plots
20. References

1. Experimental procedures

Technical grade solvents (CH_2Cl_2, *n*-hexane, and *n*-pentane) were distilled prior to use. THF was dried over sodium, CH_2Cl_2 was dried over CaH_2, and both were distilled prior to use. Anhydrous toluene (99.8%) was commercially obtained and used without further purification. Flash column chromatography was performed using Merck SiO_2 60 (230−400 mesh), thin layer chromatography was performed on commercially available Merck F_{254} pre-coated sheets. ^{1}H and ^{13}C NMR spectra were recorded on a *Bruker* Avance 400 spectrometer. Chemical shifts are given in ppm and are referenced by using the residual solvent signals as internal standard.[76] IR spectra were recorded on a Bruker Alpha FT-IR spectrometer using ATR technique and mass spectra were recorded with a Finnigan MAT-95 mass spectrometer and a Thermo Fisher Scientific Q Exactive Orbitrap mass spectrometer. CW-EPR spectra were recorded on a Bruker EMXplus Xband spectrometer (microwave frequency: 9.42 GHz) and were calibrated against DPPH (*g* = 2.0036).[77] The spectra were simulated using the MATLAB/EasySpin software package.[78] Diffraction data were measured using a Stoe STADIVARI diffractometer and Ga-Kα (1.34134 Å) radiation. Absorption corrections were carried out using the STOE LANA software package.[79] Structure solution was carried out using OLEX2 1.5[80] by dual-space direct methods with SHELXT,[81] by full-matrix least-squares refinement using SHELXL-2014/7.[82] All non-hydrogen atoms were refined anisotropically. The contribution of the hydrogen atoms, in their calculated positions, was included in the refinement using a riding model.

1-Bromo-2-(dibromomethyl)naphthalene (8)

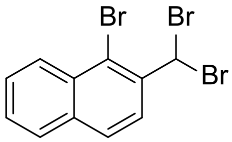

In slight variation of a published synthesis,[53] a solution of 1-bromo-2-methylnaphthalene (**7**; 2.21 g, 10.0 mmol, 1.00 equiv.), *N*-bromosuccinimide (4.45 g, 25.0 mmol, 2.50 equiv.), and benzoyl peroxide (0.484 g, 2.00 mmol, 0.200 equiv.) in anhydrous benzene (40 mL) was heated to reflux for 24 h. The mixture was cooled to rt, filtered through Celite, washed with an aqueous sodium disulfite solution (3×100 mL) and the filtrate was extracted with CH_2Cl_2 (3×100 mL). The combined organic layers were concentrated at reduced pressure and purified by column chromatography (silica gel, *n*-hexane/EtOAc 9:1) to yield **8** (3.45 g, 9.10 mmol, 91%) as an orange solid. ^{1}H NMR (400 MHz, $CDCl_3$): *δ* (ppm) 8.31 (dd, *J* = 8.6 Hz, *J* = 1.1 Hz, 1 H, Ar-H), 8.1 (d, *J* = 8.7 Hz, 1 H, Ar-H), 7.90 (d, *J* = 8.7 Hz, 1 H, Ar-H), 7.86–7.81 (m, 1 H, Ar-H), 7.64 (ddd, *J* = 8.4 Hz, *J* = 6.9 Hz, *J* = 1.4 Hz, 1 H, Ar-H), 7.61–7.53 (m, 1 H, Ar-H), 7.50 (s, 1 H, $CHBr_2$). The NMR data of the product are in agreement with published data.[53]

1-Bromo-2-naphthaldehyde (9)

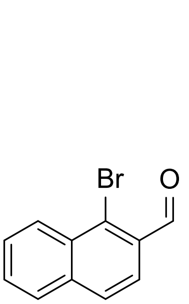

Following a published synthesis,[53] 1-bromo-2-(dibromomethyl)naphthalene (**8**; 10.0 g, 26.4 mmol, 1.00 equiv.) was added to a solution of KOAc (9.81 g, 100 mmol, 3.80 equiv.) in HOAc (130 mL) and the mixture was heated to reflux for 24 h. Aqueous HCl solution (2M; 40 mL) was added and the mixture was heated to reflux for further 4 h, cooled to rt, and extracted with EtOAc (3×200 mL). The combined organic layers were washed with H_2O (3×200 mL), saturated aqueous $NaHCO_3$ solution (3×200 mL), and brine (3×200 mL), and concentrated at reduced pressure to yield **9** (5.79 g, 24.6 mmol, 93%) as a beige solid. ^{1}H NMR (400 MHz, $CDCl_3$): *δ* (ppm) 10.66 (d, *J* = 0.9 Hz, 1 H,

CHO), 8.55–8.45 (m, 1 H, Ar-H), 7.93 (d, *J* = 8.5 Hz, 1 H, Ar-H), 7.89–7.82 (m, 2 H, Ar-H), 7.74–7.62 (m, 2 H, Ar-H). The NMR data of the product are in agreement with published data.[53]

1-(4,4,5,5-Tetramethyl-1,3,2-dioxaborolan-2-yl)-2-naphthaldehyde (10)

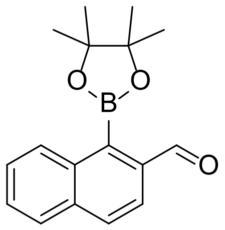

$PdCl_2$(dppf) (311 mg, 425 µmol, 0.100 equiv.) was added under an argon atmosphere to a degassed (ultrasonication, 10 min) solution of naphthaldehyde **9** (1.00 g, 4.25 mmol, 1.00 equiv.), KOAc (1.25 g, 12.8 mmol, 3.00 equiv.), and B_2Pin_2 (1.40 g, 5.53 mmol, 1.30 equiv.) in 1,4-dioxane (25 mL) and the solution was heated to 80 °C for 48 h. The mixture was cooled to rt and concentrated at reduced pressure. EtOAc (50 mL) was added and the solution was filtered (Celite), concentrated at reduced pressure, and purified by column chromatography (silica gel, *n*-hexane/EtOAc 9:1) to yield **10** (818 mg, 2.90 mmol, 68%) as a pale yellow solid. ^{1}H NMR (400 MHz, $CDCl_3$): δ (ppm) 10.28 (s, 1 H, CHO), 8.25–8.13 (m, 1 H, Ar-H), 8.00–7.85 (m, 3 H, Ar-H), 7.59 (ddd, *J* = 8.6 Hz, *J* = 6.1 Hz, *J* = 1.6 Hz, 2 H, Ar-H), 1.55 (s, 12 H, 4×CH_3). The NMR data of the product are in agreement with published data.[54]

2-Methoxy-1-naphthaldehyde (12)

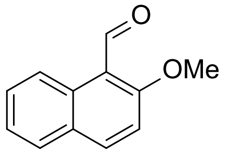

Following a published synthesis,[55] $POCl_3$ (16.5 g, 9.81 mL, 107 mmol, 3.40 equiv.) was added dropwise to a cooled solution (0 °C) of 2-methoxynaphthalene (**11**; 5.00 g, 31.6 mmol, 1.00 equiv.) in anhydrous DMF (25 mL) and the solution was stirred for 1 h at 0 °C and heated to 95 °C for 4 h. After cooling to rt, the mixture was quenched by pouring on water ice. The precipitate was filtered and the residue washed with small amounts of water and dried to yield **12** (5.52 g, 29.6 mmol, 94%) as a beige solid. The compound could be used without further purification in the next step. ^{1}H NMR (400 MHz, $CDCl_3$): δ (ppm) 10.90 (s, 1 H, CHO), 9.28 (dd, *J* = 8.7 Hz, *J* = 1.0 Hz, 1 H, Ar-H), 8.07 (d, *J* = 9.2 Hz, 1 H, Ar-H), 7.83–7.75 (m, 1 H, Ar-H), 7.62 (ddd, *J* = 8.6 Hz, *J* = 6.9 Hz, *J* = 1.5 Hz, 1 H, Ar-H), 7.42 (ddd, *J* = 8.1 Hz, *J* = 6.8 Hz, *J* = 1.2 Hz, 1 H, Ar-H), 7.31 (d, *J* = 9.1 Hz, 1 H, Ar-H), 4.06 (s, 3 H, CH_3). The NMR data of the product are in agreement with published data.[55]

2-Hydroxy-1-naphthaldehyde (13)

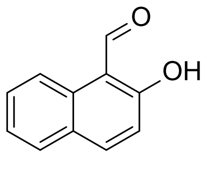

Following a published procedure,[56] $AlCl_3$ (4.14 g, 31.0 mmol, 1.50 equiv.) was added in one batch to a cooled (−5 °C) solution of methoxynaphthaldehyde **12** (3.85 g, 20.7 mmol, 1.00 equiv.) in CH_2Cl_2 (100 mL). The mixture was stirred for 5 min, was allowed to warm to rt, and stirred for further 4 h. The reaction was quenched by pouring the mixture on water ice. The mixture was extracted with CH_2Cl_2 (3×150 mL) and the combined organic layers were washed with $NaHCO_3$ (3×100 mL) and brine (3×100 mL), dried (Na_2SO_4), concentrated at reduced pressure, and purified by column chromatography (silica gel, *n*-hexane/EtOAc 9:1) to yield **13** (2.71 g, 15.7 mmol, 76%) as a pale yellow solid. ^{1}H NMR (400 MHz, $CDCl_3$, ppm) δ (ppm) 13.16 (s, 1 H, OH), 10.82 (s, 1 H, CHO), 8.35 (d, *J* = 8.6 Hz, 1 H, Ar-H), 7.98 (d, *J* = 9.1 Hz, 1 H, Ar-H), 7.88–7.78 (m, 1 H, Ar-H), 7.62 (ddd, *J* = 8.5 Hz, *J* = 7.0 Hz, *J* = 1.4 Hz, 1 H, Ar-H), 7.44 (ddd, *J* = 8.1 Hz, *J* = 6.9 Hz, *J* = 1.1 Hz, 1 H, Ar-H),

7.15 (d, J = 9.0 Hz, 1 H, Ar-H). The NMR data of the product are in agreement with published data.[56]

1-Formylnaphthalen-2-yl Trifluoromethanesulfonate (14)

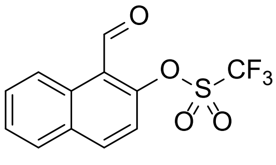

Following a published procedure,[57] trifluoromethanesulfonic anhydride (7.50 g, 4.47 mL, 26.6 mmol, 1.20 equiv.) was added under an argon atmosphere to a cooled (0 °C) solution of naphthol **13** (3.81 g, 22.2 mmol, 1.00 equiv.) and 4-(dimethylamino)pyridine (DMAP; 5.41 g, 44.3 mmol, 2.00 equiv.) in CH_2Cl_2 (60 mL), stirred for 30 min at 0 °C, allowed to warm to rt, and stirred for further 4 h. The reaction was quenched with water (50 mL) at 0 °C and extracted with CH_2Cl_2 (3×100 mL). The combined organic layers were washed with aqueous HCl (1M; 3×50 mL) and brine (3×100 mL), dried (Na_2SO_4), concentrated at reduced pressure, and purified by column chromatography (silica gel, *n*-hexane/EtOAc 9:1) to yield **14** (4.11 g, 13.5 mmol, 61%) as a beige solid. ^{1}H NMR (400 MHz, $CDCl_3$): δ (ppm) 10.80 (s, 1 H, CHO), 9.18 (dd, J = 8.8 Hz, J = 1.0 Hz, 1 H, Ar-H), 8.19 (d, J = 9.0 Hz, 1 H, Ar-H), 7.94 (dt, J = 8.3 Hz, J = 1.0 Hz, 1 H, Ar-H), 7.77 (ddd, J = 8.6 Hz, J = 6.9 Hz, J = 1.5 Hz, 1 H, Ar-H), 7.66 (ddd, J = 8.2 Hz, J = 6.9 Hz, J = 1.2 Hz, 1 H, Ar-H), 7.49 (d, J = 9.0 Hz, 1 H, Ar-H). The NMR data of the product are in agreement with published data.[57]

2-(4,4,5,5-Tetramethyl-1,3,2-dioxaborolan-2-yl)-1-naphthaldehyde (15)

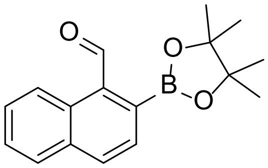

$PdCl_2$(dppf) (265 mg, 362 µmol, 0.100 equiv.) was added under an argon atmosphere to a degassed (ultrasonication, 10 min) solution of tosylate **14** (1.10 g, 3.62 mmol, 1.00 equiv.), KOAc (1.07 g, 10.9 mmol, 3.00 equiv.), and B_2Pin_2 (1.19 g, 4.70 mmol, 1.30 equiv.) in 1,4-dioxane (25 mL). Th mixture was heated to 80 °C for 48 h. After cooling to rt, the solvent was removed at reduced pressure. EtOAc (50 mL) was added and the solution was filtered (Celite), concentrated at reduced pressure, and purified by column chromatography (silica gel, *n*-hexane/EtOAc 9:1) to yield **15** (1.05 g, 3.72 mmol, quant.) as a pale yellow solid. ^{1}H NMR (400 MHz, $CDCl_3$): δ (ppm) 10.97 (s, 1 H, CHO), 9.00 (dd, J = 8.6 Hz, J = 1.1 Hz, 1 H, Ar-H), 8.05 (d, J = 8.1 Hz, 1 H, Ar-H), 7.93–7.86 (m, 1 H, Ar-H), 7.81 (d, J = 8.1 Hz, 1 H, Ar-H), 7.65 (ddd, J = 8.5 Hz, J = 6.8 Hz, J = 1.5 Hz, 1 H, Ar-H), 7.58 (ddd, J = 8.1 Hz, J = 6.8 Hz, J = 1.2 Hz, 1 H, Ar-H), 1.45 (s, 12 H, 4×CH_3); ^{13}C NMR (100 MHz, $CDCl_3$): δ (ppm) 194.8 (CHO), 137.0 (C), 134.7 (C), 133.5 (CH), 131.0 (CH), 129.9 (CH), 128.7 (CH), 128.6 (CH), 127.3 (CH), 124.6 (CH), 84.7 (C-4', C-5'), 25.0 (4×CH_3), one signal (C-2) was not observed due to the quadrupole moment of boron*; IR* (ATR): $\tilde{\nu}$ (cm^{-1}) = 3471 (w), 3455 (w), 3437 (w), 3422 (w), 3415 (w), 3407 (w), 3397 (w), 3383 (w), 3295 (w), 2979 (m), 2944 (w), 1681 (w), 1469 (m), 1370 (vs), 1349 (s), 1326 (s), 1275 (w), 1259 (w), 1213 (w), 1143 (vs), 1115 (vs), 1061 (w), 1048 (w), 1006 (w), 983 (w), 949 (vs), 884 (m), 864 (w), 850 (m), 827 (m), 781 (w), 775 (w), 751 (m), 711 (w), 697 (w), 671 (m), 649 (m), 639 (m), 618 (m), 609 (m), 596 (m), 578 (m), 552 (s), 521 (s), 510 (s), 496 (s), 479 (m), 465 (m), 458 (m), 449 (s), 442 (m), 428 (s), 407 (m), 398 (m), 381 (m); MS (ESI): *m/z* (%): 284.1 (4) [M+2]$^+$, 283.1 (23) [M+1]$^+$, 282.1 (6) [M]$^+$, 592.2 (3) [M]$^+$; HRMS (ESI): *m/z* calcd for $C_{17}H_{19}BO_3$: 282.1427 [M]$^+$; found: 282.1535.

2,7-Dibromo-9-mesityl-9*H*-fluorene (17)

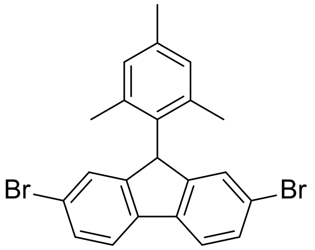

Following a published method,[58] Me_3SiCl (1.06 g, 1.24 mL, 9.76 mmol, 1.10 equiv.) and Et_3SiH (1.09 g, 1.50 mL, 9.37 mmol, 1.06 equiv.) were added under an argon atmosphere to a solution of fluorenone **16** (3.00 g, 8.88 mmol, 1.00 equiv.) and anhydrous $FeCl_3$ (72.0 mg, 444 µmol, 0.05 equiv.) in mesitylene (8 mL). The mixture was stirred for 2 h at rt, heated for 18 h to 50 °C, cooled to rt, quenched with H_2O (20 mL), and extracted with CH_2Cl_2 (3×100 mL). The combined organic layers were washed with H_2O (3×100 mL), dried (Na_2SO_4), concentrated at reduced pressure, and purified by column chromatography (silica gel, *n*-hexane) to yield **17** (2.89 g, 6.54 mmol, 74%) as pale orange crystals. R_f = 0.38 (*n*-hexane); ^{1}H NMR (400 MHz, $CDCl_3$): δ (ppm) 7.65 (d, 2 H, Ar-H), 7.55–7.48 (m, 2 H, Ar-H), 7.33 (t, J = 1.5 Hz, 2 H, Ar-H), 7.03–7.01 (m, 1 H, Ar-H), 6.72–6.65 (m, 1 H, Ar-H), 5.44 (s, 1 H, Ar-H), 2.64 (s, 3 H, CH_3), 2.30 (s, 3 H, CH_3), 1.13 (s, 3 H, CH_3); ^{13}C NMR (100 MHz, $CDCl_3$): δ (ppm) 149.1 (C), 138.9 (C), 137.8 (C), 137.8 (C), 137.1 (C), 132.3 (C), 130.9 (CH), 130.4 (CH), 129.2 (CH), 127.5 (CH), 121.7 (C), 121.5 (CH), 49.7 (CH), 21.8 (CH_3), 21.0 (CH_3), 18.9 (CH_3); IR (ATR): $\tilde{\nu}$ (cm^{-1}) = 2949 (w), 2918 (w), 2860 (w), 1881 (vw), 1734 (vw), 1596 (w), 1568 (w), 1482 (w), 1455 (s), 1443 (m), 1411 (m), 1401 (m), 1394 (m), 1377 (w), 1258 (w), 1235 (w), 1162 (w), 1058 (s), 1031 (w), 1004 (m), 898 (m), 878 (m), 868 (w), 853 (m), 830 (w), 810 (vs), 796 (vs), 768 (w), 756 (w), 663 (m), 616 (m), 465 (s), 414 (m); MS (FAB): *m/z* (%): 443.9 (10) $[M]^+$, 442.9 (6) $[M]^+$, 440.9 (5) $[M]^+$, 363.0 (7) $[M-Br]^+$, 361.0 (7) $[M-Br]^+$, 324.8 (4) $[M-Mes]^+$, 322.8 (8) $[M-Mes]^+$, 320.8 (4) $[M-Mes]^+$; HRMS (FAB): *m/z* calcd for $C_{22}H_{18}{}^{79}Br^{81}Br$: 441.9749 $[M]^+$; found: 441.9751.

1,1'-(9-Mesityl-9*H*-fluorene-2,7-diyl)bis(2-naphthaldehyde) (18)

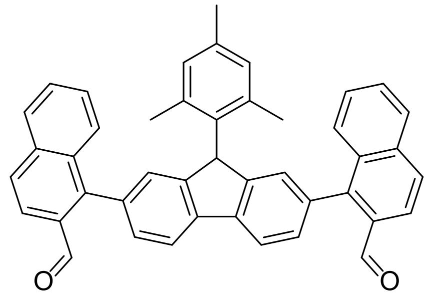

$PdCl_2$(dppf) (12.4 mg, 17.0 µmol, 0.15 equiv.) was added under an argon atmosphere to a degassed (ultrasonication, 10 min) mixture of dibromofluorene **17** (50 mg, 113 µmol, 1.00 equiv.), boronate **10** (80.0 mg, 283 µmol, 2.50 equiv.), and Cs_2CO_3 (184 mg, 565 µmol, 5.00 equiv.) in toluene/H_2O (1:1; 10 mL). The mixture was heated to 105 °C for 18 h, cooled to rt, and the aqueous layer was extracted with CH_2Cl_2 (3×100 mL). The combined organic layers were dried (Na_2SO_4), concentrated at reduced pressure, and purified by column chromatography (silica gel, *n*-hexane/EtOAc 9:1) to yield **18** (45.0 mg, 76.0 µmol, 67%) as a beige solid. The product was obtained as a mixture of isomers, thus resulting in additional hardly discriminable signals in the spectra. R_f = 0.50 (*n*-hexane/EtOAc 9:1); ^{1}H NMR (400 MHz, $CDCl_3$): δ (ppm) 10.07 (s, 1 H, CHO), 9.87 (s, 1 H, CHO), 8.16–7.99 (m, 4 H, Ar-H), 7.92 (m, J = 8.8 Hz, J = 5.4 Hz, 4 H, Ar-H), 7.78 (m, J = 8.0 Hz, 1 H, Ar-H), 7.67–7.56 (m, 3 H, Ar-H), 7.55–7.47 (m, 3 H, Ar-H), 7.46–7.39 (m, 1 H, Ar-H), 7.31 (m, J = 8.4 Hz, 2 H, Ar-H), 6.85 (s, 1 H, Ar-H), 6.66 (d, J = 6.0 Hz, 1 H, Ar-H), 5.73 (m, J = 10.4 Hz, 1 H, Ar-H), 2.58 (m, J = 4.0 Hz, 3 H, CH_3), 2.16 (m, J = 5.8 Hz, 3 H, CH_3), 1.48–1.28 (m, 3 H, CH_3); ^{13}C NMR (100 MHz, $CDCl_3$): δ (ppm) 193.0 (CHO), 192.6 (CHO), 148.0 (C), 146.8 (C), 140.6 (C), 140.5 (C), 136.2 (C), 134.8 (C), 132.8 (C), 131.5 (C), 131.3 (C), 130.9 (C), 130.4 (CH), 130.2 (CH), 129.1 (CH), 129.0 (CH), 128.9 (CH), 128.8 (CH), 128.5 (CH), 128.4 (CH), 127.9 (CH), 127.8 (CH), 127.6 (CH), 127.1 (CH), 127.1 (CH), 127.0 (CH), 126.8 (CH), 122.3 (CH), 122.2 (CH), 120.3 (CH), 50.1 (CH), 21.9 (CH_3), 20.9 (CH_3), 18.9 (CH_3); IR (ATR): $\tilde{\nu}$ (cm^{-1}) = 3058 (vw), 2956 (w), 2918 (w), 2850 (w), 2731 (w), 2322 (w), 2245 (vw), 2167 (vw), 1983

(vw), 1939 (vw), 1680 (vs), 1612 (w), 1594 (w), 1571 (w), 1460 (m), 1428 (m), 1411 (w), 1378 (s), 1332 (m), 1261 (m), 1232 (s), 1211 (m), 1188 (m), 1150 (m), 1086 (s), 1062 (s), 1028 (vs), 1018 (s), 1013 (s), 919 (s), 870 (s), 850 (s), 819 (vs), 800 (vs), 790 (vs), 771 (vs), 747 (vs), 727 (vs), 688 (s), 616 (s), 578 (m), 540 (m), 523 (s), 513 (s), 486 (vs), 470 (vs), 459 (vs), 450 (vs), 441 (vs), 433 (vs), 428 (vs), 414 (vs), 388 (vs); MS (ESI): *m/z* (%): 595.2 (7) [*M*+3]$^+$, 594.2 (33) [*M*+2]$^+$, 593.2 (71) [*M*+1]$^+$, 592.2 (3) [*M*]$^+$, 565.4 (19) [*M*+2–CHO]$^+$, 564.4 (75) [*M*+1–CHO]$^+$, 563.5 (77) [*M*–CHO]$^+$; HRMS (ESI): *m/z* calcd for $C_{44}H_{32}O_2$: 592.2402 [*M*]$^+$; found: 592.2412.

2,2'-(9-Mesityl-9*H*-fluorene-2,7-diyl)bis(1-naphthaldehyde) (19)

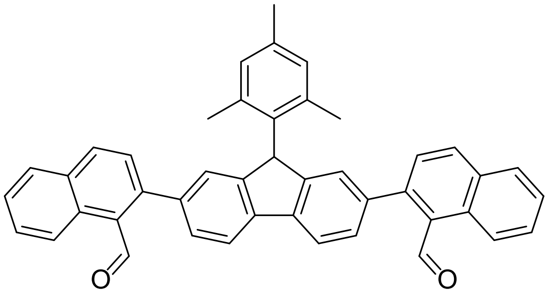

$PdCl_2$(dppf) (112 mg, 153 µmol, 0.15 equiv.) was added under an argon atmosphere to a degassed (ultrasonication, 10 min) mixture of dibromofluorene **17** (450 mg, 1.02 mmol, 1.00 equiv.), boronate **15** (718 mg, 2.54 mmol, 2.50 equiv.), and Cs_2CO_3 (1.66 g, 5.09 mmol, 5.00 equiv.) in toluene/H_2O (1:1; 20 mL). The mixture was heated to 105 °C for 18 h, cooled to rt, and the aqueous layer was extracted with CH_2Cl_2 (3×100 mL). The combined organic layers were dried (Na_2SO_4), concentrated at reduced pressure, and purified by column chromatography (silica gel, *n*-hexane/EtOAc 9:1) to yield **19** (266 mg, 0.450 mmol, 44%) as a beige solid. R_f = 0.33 (*n*-hexane/EtOAc 9:1); ^{1}H NMR (400 MHz, $CDCl_3$): *δ* (ppm) 10.22 (s, 2 H, CHO), 9.29–9.19 (m, 2 H, Ar-H), 8.03 (m, *J* = 17.1 Hz, *J* = 8.1 Hz, 4 H, Ar-H), 7.90 (m, *J* = 8.3 Hz, *J* = 1.4 Hz, 2 H, Ar-H), 7.73–7.66 (m, 2 H, Ar-H), 7.58 (m, *J* = 8.1 Hz, *J* = 6.9 Hz, *J* = 1.2 Hz, 2 H, Ar-H), 7.54 (m, *J* = 8.4 Hz, 2 H, Ar-H), 7.51 (m, *J* = 7.0 Hz, *J* = 1.3 Hz, 2 H, Ar-H), 7.36 (m, 2 H, Ar-H), 6.97 (s, *J* = 1.9 Hz, 1 H, Ar-H), 6.69 (s, *J* = 1.9 Hz, 1 H, Ar-H), 5.69 (s, 1 H, Ar-H), 2.68 (s, 3 H, CH_3), 2.23 (s, 3 H, CH_3), 1.25 (s, 3 H, CH_3); ^{13}C NMR (100 MHz, $CDCl_3$): *δ* (ppm) 194.6 (CHO), 148.2 (C), 148.1 (C), 140.5, (C) 138.4 (C), 137.9 (C), 137.6 (C), 136.9 (C), 134.0 (CH), 133.1 (C), 132.9 (C), 130.9 (CH), 130.5 (C), 130.3 (CH), 129.4 (CH), 129.2 (CH), 129.0 (C), 128.5 (CH), 128.4 (CH), 126.9 (CH), 126.2 (CH), 125.8 (CH), 120.3 (CH), 50.1 (CH), 22.0 (CH_3), 21.0 (CH_3), 19.1 (CH_3); IR (ATR): $\tilde{\nu}$ (cm^{-1}) = 3050 (w), 3033 (w), 3012 (w), 3003 (w), 2946 (w), 2917 (w), 2851 (w), 2798 (w), 2755 (w), 1679 (vs), 1615 (w), 1589 (m), 1557 (w), 1504 (m), 1475 (m), 1455 (m), 1429 (m), 1408 (m), 1375 (w), 1353 (w), 1346 (w), 1310 (w), 1298 (w), 1264 (w), 1241 (w), 1213 (w), 1174 (m), 1146 (m), 1109 (w), 1055 (s), 1026 (w), 1016 (w), 1004 (w), 953 (w), 899 (w), 881 (w), 850 (w), 819 (vs), 800 (vs), 782 (m), 751 (vs), 735 (vs), 710 (s), 698 (s), 679 (w), 640 (m), 609 (m), 575 (w), 550 (m), 509 (s), 467 (m), 460 (m), 422 (m), 411 (m), 394 (w), 384 (w); MS (ESI): *m/z* (%): 595.3 (1) [*M*+3]$^+$, 594.3 (9) [*M*+2]$^+$, 593.2 (19) [*M*+1]$^+$, 592.2 (1) [*M*]$^+$, 565.5 (7) [*M*+2–CHO]$^+$, 564.5 (39) [*M*+1–CHO]$^+$, 563.5 (100) [*M*–CHO]$^+$; HRMS (ESI): *m/z* calcd for $C_{44}H_{32}O_2$: 592.2402 [*M*]$^+$; found: 592.2347.

10,18-Dihydro-7,10,18-trimesityl-7*H*-bisbenz[6,7]indeno[1,2-*b*:2′,1′-*h*]fluorene (22)

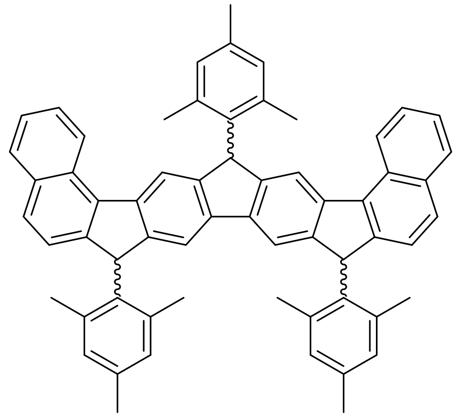

In accordance to a published method,[59] MesMgBr (1.00 M in THF; 84.0 mg, 0.38 mL, 380 µmol, 5.00 equiv.) was added dropwise under an argon atmosphere to a cooled (0 °C) solution of biscarbaldehyde **18** (45.0 mg, 76.0 µmol, 1.00 equiv.) in anhydrous THF (10 mL). After addition was completed, the cooling bath was removed and the mixture was stirred for 15 min at rt. Saturated aqueous NH_4Cl solution (20 mL) was added, stirring was continued for 10 min, and the mixture was extracted with CH_2Cl_2 (3×100 mL). The combined organic layers were dried (Na_2SO_4) and concentrated at reduced pressure. The crude remnant **20** was dissolved in anhydrous CH_2Cl_2 (10 mL) and the solution was cooled to 0 °C. $BF_3{\cdot}OEt_2$ (74.1 mg, 66 µL, 522 µmol, 5.00 equiv.) was added under an argon atmosphere and the mixture was stirred for 1 h at rt. Saturated aqueous NH_4Cl solution (20 mL) was added, stirring was continued for 10 min, and the mixture was extracted with CH_2Cl_2 (3×100 mL). The combined organic layers were dried (Na_2SO_4), concentrated at reduced pressure, and purified by column chromatography (silica gel, n-pentane/CH_2Cl_2) to yield **22** (66.0 mg, 83.0 µmol, 79%) as a pale orange solid. The product was obtained as a mixture of isomers, thus resulting in sets of hardly discriminable signals in the spectra. Only the most pronounced signals are given. R_f = 0.43 (*n*-hexane/CH_2Cl_2 4:1); ^{1}H NMR (400 MHz, $CDCl_3$): *δ* (ppm) 8.63 (m, *J* = 8.0 Hz, 2 H), 8.26–8.12 (m, 2 H), 7.92 (m, *J* = 8.4 Hz, *J* = 3.9 Hz, 2 H), 7.72 (m, *J* = 8.3 Hz, *J* = 4.3 Hz, 2 H), 7.67–7.57 (m, 4 H), 7.50 (m, *J* = 7.8 Hz, *J* = 3.3 Hz, 2 H), 7.33 (m, *J* = 8.2 Hz, *J* = 6.1 Hz, 2 H), 7.25–7.20 (m, 1 H), 7.12–7.06 (m, 2 H), 6.72–6.60 (m, 3 H), 5.84 (m, 1 H), 5.60 (m, *J* = 5.8 Hz, 2 H), 2.99 (m, *J* = 7.0 Hz, 3 H), 2.78 (m, *J* = 4.3 Hz, 6 H), 2.40–2.20 (m, 9 H), 1.14–1.00 (m, 9 H); ^{13}C NMR (100 MHz, $CDCl_3$): *δ* (ppm) 147.5 (C), 147.1 (C), 146.6 (C), 141.4 (C), 139.6 (C), 138.2 (C), 138.0 (C), 136.4 (C), 135.4 (C), 133.8 (C), 133.6 (C), 130.7 (CH), 130.5 (CH), 129.4 (CH), 129.2 (CH), 129.0 (CH), 127.9 (CH), 126.6 (CH), 125.0 (CH), 124.1 (CH), 122.3 (CH), 118.7 (CH), 115.8 (CH), 50.0 (CH), 22.0 (CH_3), 20.9 (CH_3), 18.4 (CH_3); IR (ATR) $\tilde{\nu}$ (cm^{-1}) = 3048 (m), 3012 (s), 2956 (vs), 2918 (vs), 2853 (vs), 2819 (m), 2555 (m), 2517 (m), 2448 (m), 2360 (m), 2252 (m), 2170 (m), 2027 (m), 1890 (w), 1728 (w), 1612 (m), 1562 (m), 1516 (m), 1477 (s), 1453 (vs), 1378 (s), 1361 (vs), 1307 (vs), 1220 (m), 1142 (m), 1033 (m), 1013 (m), 1001 (m), 933 (m), 878 (m), 844 (s), 823 (s), 798 (s), 785 (vs), 773 (vs), 765 (vs), 732 (vs), 710 (w), 656 (w), 613 (w), 578 (m), 557 (w), 543 (m), 504 (w), 463 (w), 429 (w), 411 (m), 397 (m); MS (ESI): *m/z* (%): 799.3 (2) $[M+3]^+$, 798.3 (4) $[M+2]^+$, 797.3 (8) $[M+1]^+$, 796.3 (5) $[M]^+$, 795.3 (9) $[M-1]^+$; HRMS (ESI): *m/z* calcd for $C_{62}H_{52}$: 796.4069 $[M]^+$; found: 796.3495.

16,19-Dihydro-8,16,19-trimesityl-8*H*-bisbenz[4,5]indeno[1,2-*b*:2′,1′-*h*]fluorene (23)

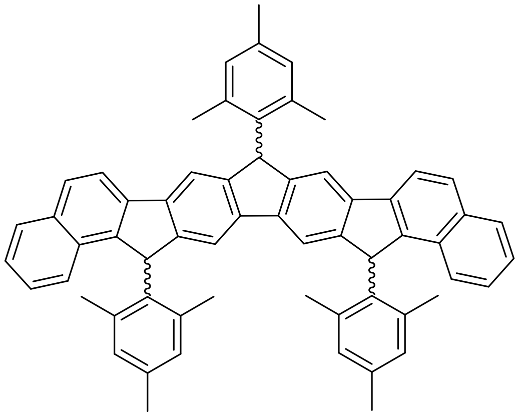

In accordance to a published method,[59] MesMgBr (1.00M in THF; 678 mg, 3.0 mL, 3.04 mmol, 5.00 equiv.) was added dropwise under an argon atmosphere to a cooled (0 °C) solution of biscarbaldehyde **19** (360 mg, 0.607 mmol, 1.00 equiv.) in anhydrous THF (10 mL). After addition was completed, the cooling bath was removed and the mixture stirred for 15 min at rt. Saturated aqueous NH_4Cl solution (20 mL) was added, stirring was continued for 10 min, and the mixture was extracted with CH_2Cl_2 (3×100 mL). The combined organic layers were dried (Na_2SO_4) and concentrated at reduced pressure. The crude remnant **21** was dissolved in anhydrous CH_2Cl_2 (20 mL) and the solution was cooled to 0 °C. $BF_3{\cdot}OEt_2$ (517 mg, 462 µL, 3.64 mmol, 5.00 equiv.) was added under an argon atmosphere and the mixture was stirred for 1 h at rt. Saturated aqueous NH_4Cl solution (20 mL) was added, stirring was continued for 10 min, and the mixture was extracted with CH_2Cl_2 (3×100 mL). The combined organic layers were dried (Na_2SO_4), concentrated at reduced pressure, and purified by column chromatography (silica gel, n-pentane/CH_2Cl_2) to yield **23** (378 mg, 474 µmol, 78%) as a pale orange solid. The product was obtained as a mixture of isomers, thus resulting in sets of hardly discriminable signals in the spectra. Only the most pronounced signals are given. R_f = 0.37 (*n*-hexane/CH_2Cl_2 4:1); ^{1}H NMR (400 MHz, $CDCl_3$): *δ* (ppm) 7.91–7.81 (m, 6 H), 7.70 (m, *J* = 13.8 Hz, 3.0 Hz, 2 H), 7.65–7.56 (m, 2 H), 7.46 (m, *J* = 8.0 Hz, *J* = 2.3 Hz, 2 H), 7.39–7.32 (m, 2 H), 7.29 (m, *J* = 5.9 Hz, *J* = 1.5 Hz, 2 H), 7.22–7.12 (m, 2 H), 6.79–6.57 (m, 3 H), 5.85–5.67 (m, 3 H), 2.96–2.86 (m, 9 H), 2.40–2.27 (m, 9 H), 1.22–0.97 (m, 9 H); ^{13}C NMR (100 MHz, $CDCl_3$): *δ* (ppm) 147.1 (C), 147.0 (C), 146.9 (C), 146.6 (C), 146.5 (C), 146.4 (C), 146.4 (C), 143.3 (C), 143.2 (C), 143.2 (C), 141.1 (C), 141.1 (C), 141.0 (C), 140.7 (C), 140.6 (C), 140.6 (C), 138.8 (C), 138.3 (C), 138.3 (C), 138.3 (C), 138.1 (C), 137.9 (C), 137.2 (C), 137.1 (C), 136.9 (C), 136.5 (C), 136.4 (C), 136.3 (C), 135.4 (C), 135.3 (C), 135.2 (C), 134.9 (C), 133.2 (C), 131.0 (CH), 130.8 (CH), 130.8 (CH), 130.7 (CH), 129.4 (CH), 129.3 (CH), 129.1 (CH), 128.2 (CH), 128.1 (CH), 127.0 (CH), 126.5 (CH), 125.2 (CH), 123.9 (CH), 119.0 (CH), 116.0 (CH), 115.9 (CH), 115.4 (CH), 49.7 (CH), 49.6 (CH), 49.5 (CH), 22.5 (CH_3), 22.5 (CH_3), 22.2 (CH_3), 21.4 (CH_3), 21.1 (CH_3), 21.1 (CH_3), 18.8 (CH_3), 18.4 (CH_3), 18.1 (CH_3); IR (ATR) $\tilde{\nu}$ (cm^{-1}) = 3053 (w), 3003 (w), 2951 (w), 2917 (m), 2853 (w), 2731 (w), 1723 (w), 1611 (w), 1579 (vw), 1519 (vw), 1479 (m), 1458 (m), 1431 (m), 1375 (w), 1366 (w), 1307 (s), 1262 (w), 1244 (w), 1197 (w), 1142 (w), 1033 (w), 1016 (w), 933 (w), 887 (m), 860 (w), 844 (m), 816 (vs), 795 (vs), 744 (vs), 698 (w), 595 (w), 564 (w), 543 (w), 516 (w), 492 (w), 466 (s), 424 (w), 409 (w)*; MS* (ESI): *m/z* (%): 798.4 (1) [*M*+2]$^+$, 797.4 (3) [*M*+1]$^+$, 796.4 (5) [*M*]$^+$; HRMS (ESI): *m/z* calcd for $C_{62}H_{52}$: 796.4069 [*M*]$^+$; found: 796.4062.

7,10,18-Trimesitylbisbenz[6,7]indeno[1,2-*b*:2′,1′-*h*]fluorenyl Radical (24)

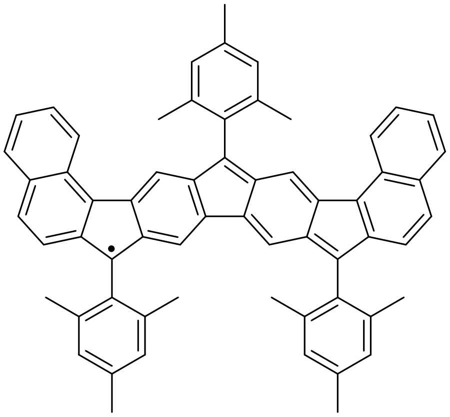

In accordance to a published method,[20] precursor **22** (70.0 mg, 88.0 µmol, 1.00 equiv.) and *t*BuOK (138 mg, 1.23 mmol, 14.0 equiv.) were dissolved in anhydrous THF (10 mL) under an argon atmosphere and heated for 16 h to 60 °C. The mixture was allowed to cool to rt and *p*-chloranil (tetrachloro-*p*-benzoquinone; 86.0 mg, 351 µmol, 4.50 equiv.) was added. After stirring for 10 min, the mixture was concentrated at reduced pressure. The obtained remnant was purified by column chromatography (silica gel, *n*-pentane/CH_2Cl_2 4:1) to yield **24** (67.0 mg, 84.4 µmol, 96%) as a dark green solid. R_f = 0.30 (*n*-hexane/CH_2Cl_2 4:1); m.p. 432 °C; IR (ATR) $\tilde{\nu}$ (cm^{-1}) = 3356 (w), 2953 (m), 2919 (s), 2851 (s), 2728 (w), 1687 (s), 1677 (vs), 1650 (m), 1562 (s), 1520 (m), 1504 (m), 1489 (m), 1456 (m), 1375 (w), 1337 (m), 1316 (w), 1256 (m), 1232 (m), 1210 (m), 1183 (w), 1105 (vs), 1035 (m), 1014 (m), 972 (w), 905 (w), 888 (m), 850 (m), 810 (m), 744 (vs), 711 (vs), 613 (w), 586 (vw), 543 (w), 510 (w), 469 (w), 463 (w), 414 (w); UV/Vis (CH_2Cl_2): λ_{max} (ε) [nm ($mol^{-1}dm^3cm^{-1}$)] = 295 (39,000), 375 (26,000), 390 (32,000), 495 (2,000), 620 (7,000), 670 (31,000), 735 (5,000), 825 (2,000); MS (ESI): *m/z* (%): 796.4 (5) $[M+3]^+$, 795.4 (23) $[M+2]^+$, 794.4 (67) $[M+1]^+$, 793.4 (100) $[M]^+$; HRMS (ESI): *m/z* calcd for $C_{62}H_{49}$: 793.3834 $[M]^+$; found: 793.3818.

8,16,19-Trimesitylbisbenz[4,5]indeno[1,2-*b*:2′,1′-*h*]fluorenyl Radical (25)

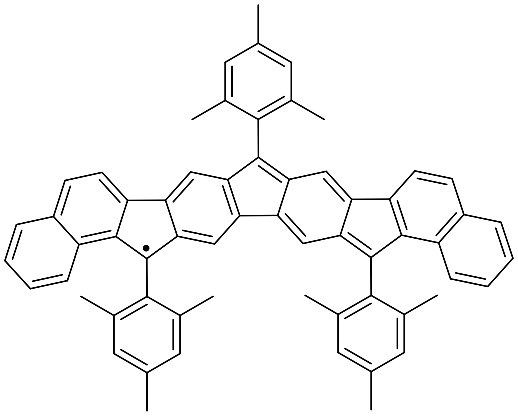

In accordance to a published method,[20] precursor **23** (55.0 mg, 69.0 µmol, 1.00 equiv.) and *t*BuOK (108 mg, 966 µmol, 14.0 equiv.) were dissolved in anhydrous THF (10 mL) under an argon atmosphere and heated for 16 h to 60 °C. The mixture was allowed to cool to rt and *p*-chloranil (tetrachloro-*p*-benzoquinone; 76.4 mg, 311 µmol, 4.50 equiv.) was added. After stirring for 10 min, the mixture was concentrated at reduced pressure. The obtained remnant was purified by column chromatography (silica gel, *n*-pentane/CH_2Cl_2 4:1) to yield **25** (35.0 mg, 44.1 µmol, 64%) as a dark blue solid. R_f = 0.48 (*n*-hexane/CH_2Cl_2 4:1); m.p. 422 °C; IR (ATR) $\tilde{\nu}$ (cm^{-1}) = 3376 (w), 3342 (m), 3330 (m), 3319 (m), 3296 (w), 3043 (w), 2952 (m), 2918 (vs), 2871 (m), 2850 (s), 1725 (vw), 1690 (w), 1626 (vs), 1611 (s), 1596 (m), 1587 (m), 1561 (vs), 1543 (vs), 1483 (m), 1458 (m), 1442 (s), 1384 (s), 1354 (s), 1343 (s), 1303 (vs), 1285 (vs), 1247 (s), 1217 (m), 1205 (s), 1177 (vs), 1154 (vs), 1133 (vs), 1116 (s), 1098 (m), 1089 (m), 1064 (m), 1037 (m), 1010 (s), 1001 (s), 982 (m), 956 (m), 899 (vs), 884 (s), 864 (m), 850 (s), 813 (vs), 795 (m), 790 (m), 782 (m), 771 (s), 747 (vs), 738 (vs), 705 (vs), 657 (m), 636 (w), 628 (w), 609 (m), 579 (m), 571 (m), 541 (m), 509 (m), 467 (m), 459 (m), 401 (w), 388 (m); UV/Vis (CH_2Cl_2): λ_{max} (ε) [nm ($mol^{-1}dm^3cm^{-1}$)] = 296 (20,000), 362 (58,000), 575 (14,000), 620 (18,000), 660 (20,000); MS (ESI): *m/z* (%): 796.4 (4) $[M+3]^+$, 795.4 (22) $[M+2]^+$, 794.4 (68) $[M+1]^+$, 793.4 (100) $[M]^+$; HRMS (ESI): *m/z* calcd for $C_{62}H_{49}$: 793.3834 $[M]^+$; found: 793.3850.

2. NMR Spectra of all Compounds, Mass and IR Spectra

^{1}H NMR spectrum of 1-bromo-2-(dibromomethyl)naphthalene (**8**, 400 MHz, $CDCl_3$)

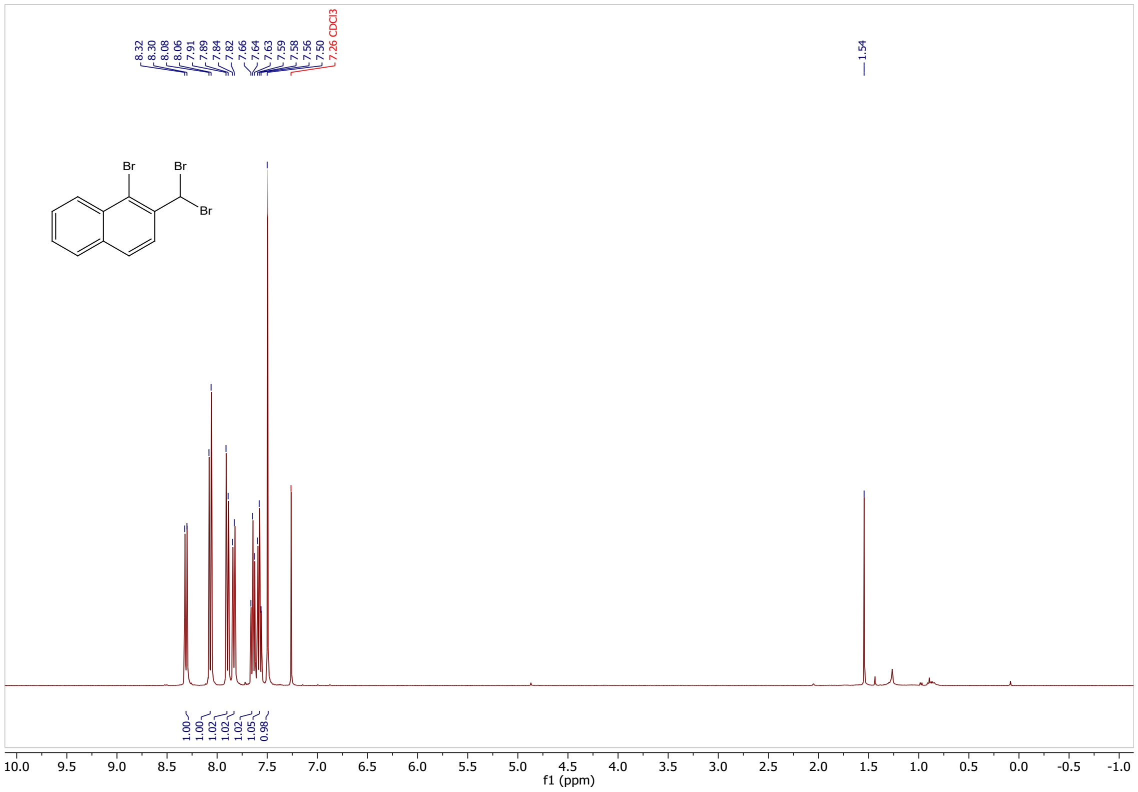

^{1}H NMR spectrum of 1-bromo-2-naphthaldehyde (**9**, 400 MHz, $CDCl_3$)

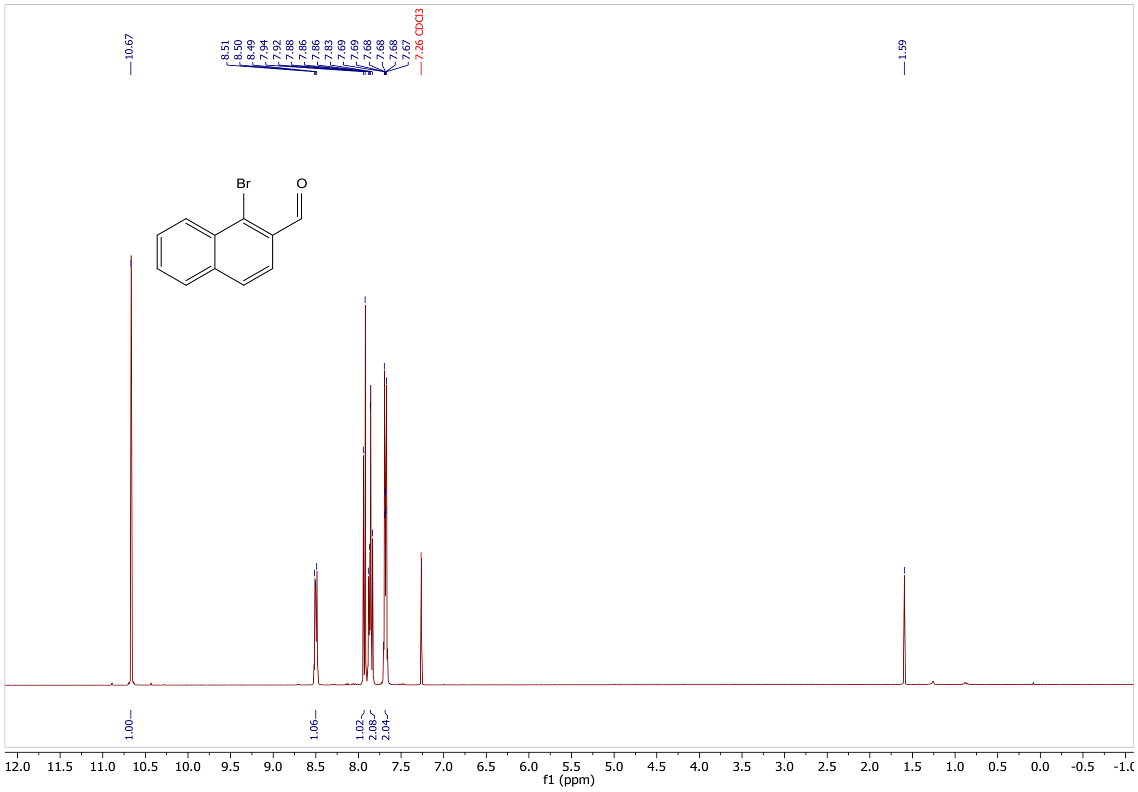

^{1}H NMR spectrum of 1-(4,4,5,5-tetramethyl-1,3,2-dioxaborolan-2-yl)-2-naphthaldehyde (**10**, 400 MHz, $CDCl_3$)

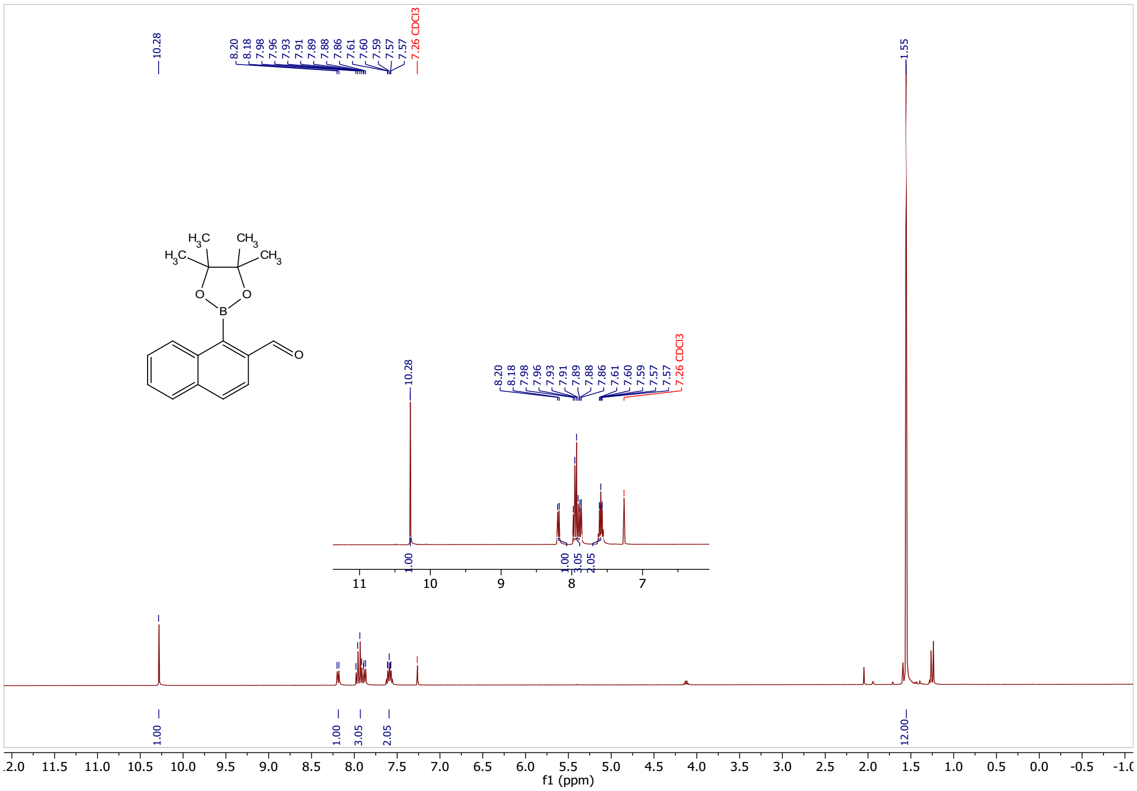

^{1}H NMR spectrum of 2-methoxy-1-naphthaldehyde (**12**, 400 MHz, $CDCl_3$)

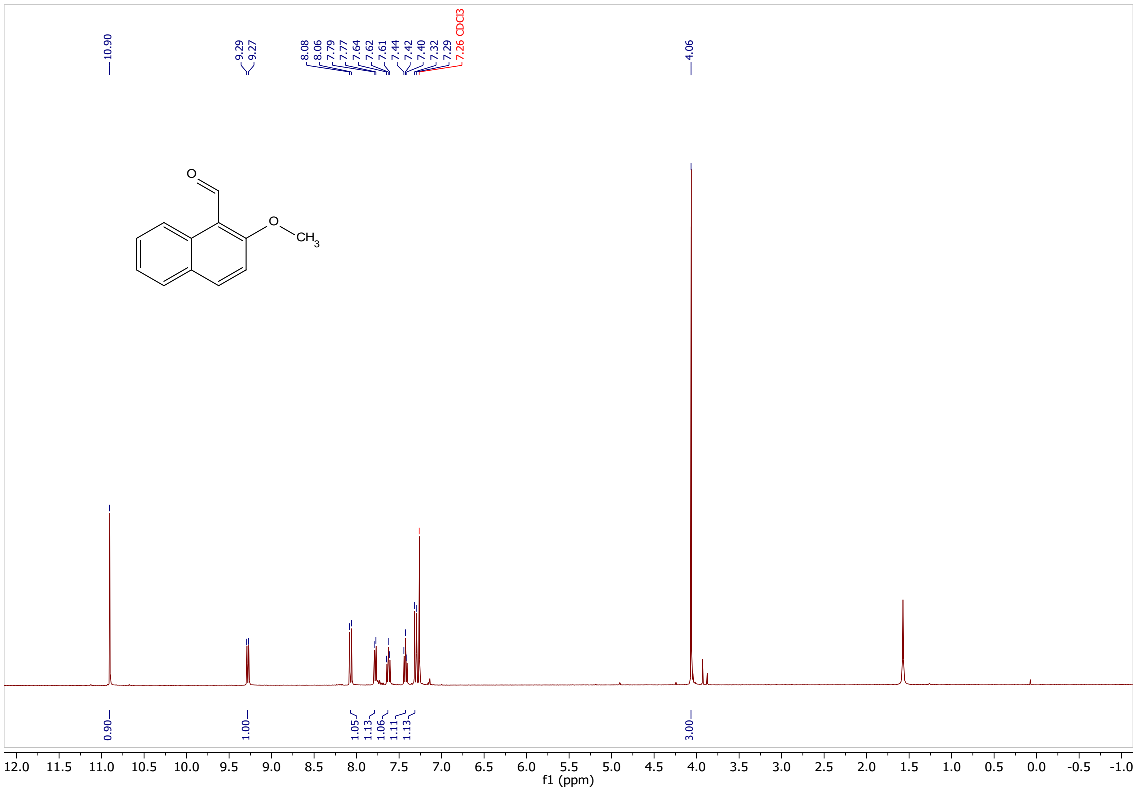

^{1}H NMR spectrum of 2-hydroxy-1-naphthaldehyde (**13**, 400 MHz, $CDCl_3$)

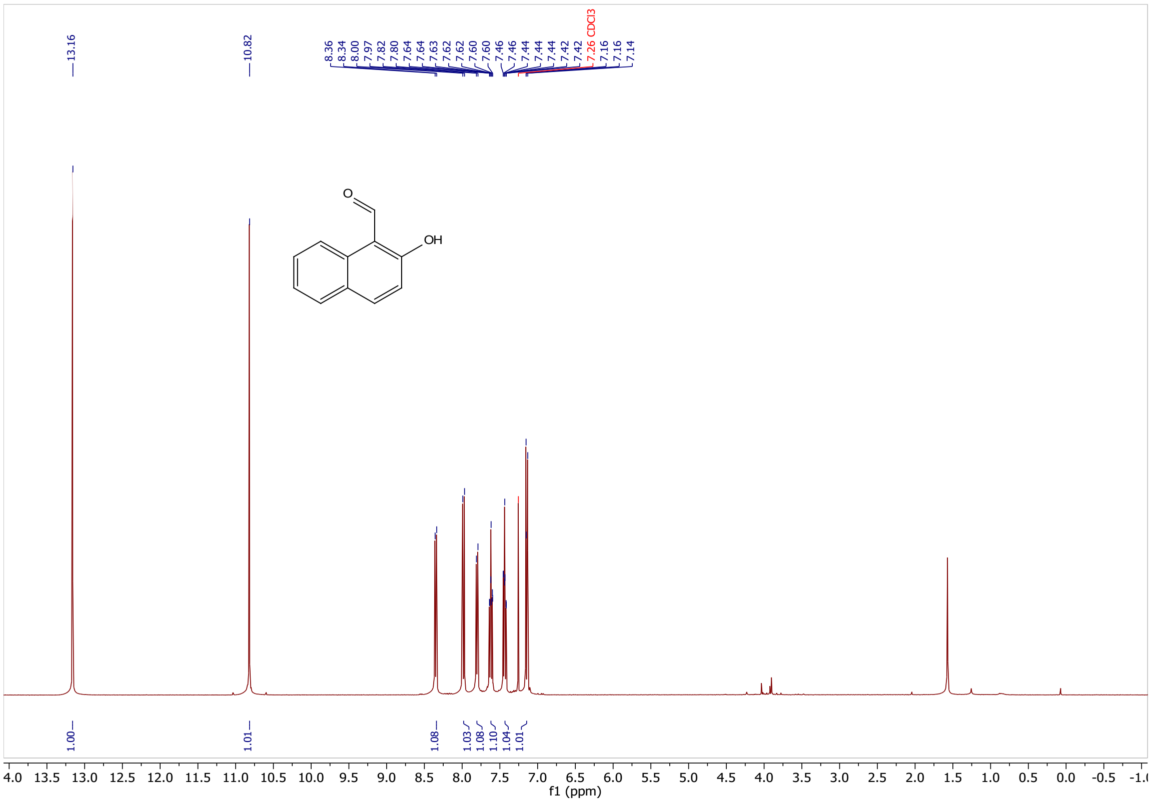

^{1}H NMR spectrum of 1-formylnaphthalen-2-yl trifluoromethanesulfonate (**14**, 400 MHz, $CDCl_3$)

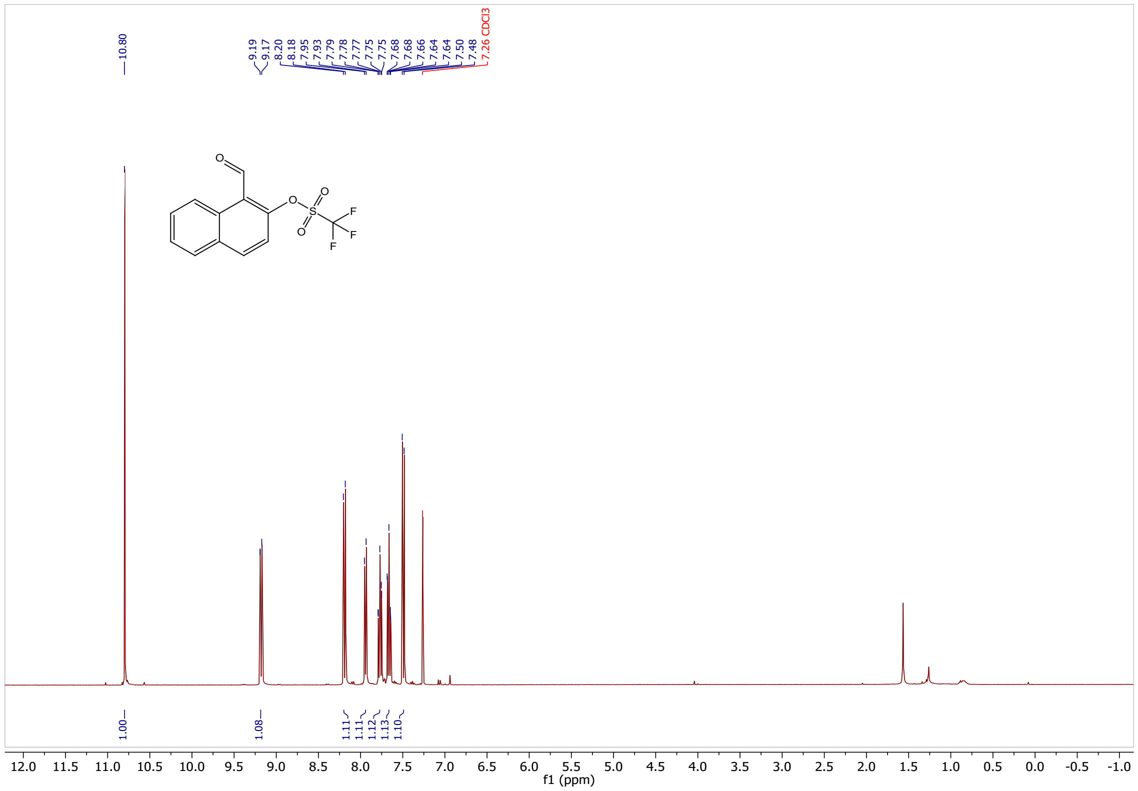

^{1}H NMR spectrum of 2-(4,4,5,5-tetramethyl-1,3,2-dioxaborolan-2-yl)-1-naphthaldehyde (**15**, 400 MHz, $CDCl_3$)

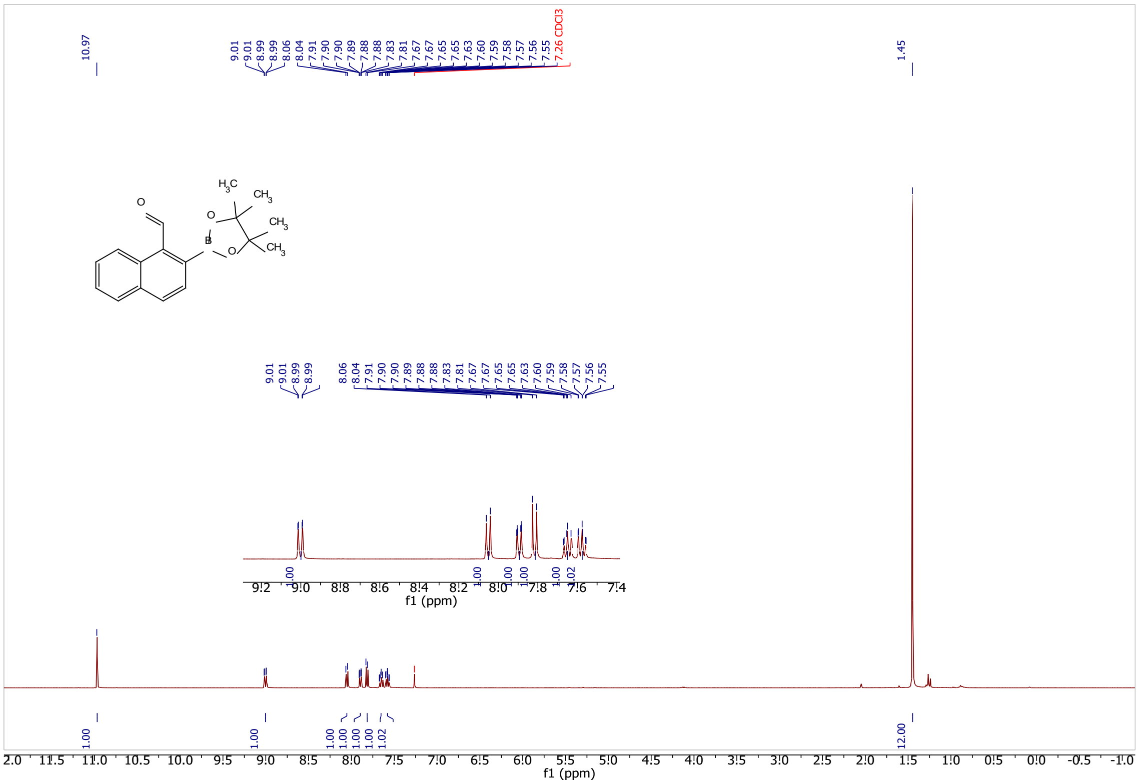

^{13}C NMR spectrum of 2-(4,4,5,5-tetramethyl-1,3,2-dioxaborolan-2-yl)-1-naphthaldehyde (**15**, 100 MHz, $CDCl_3$)

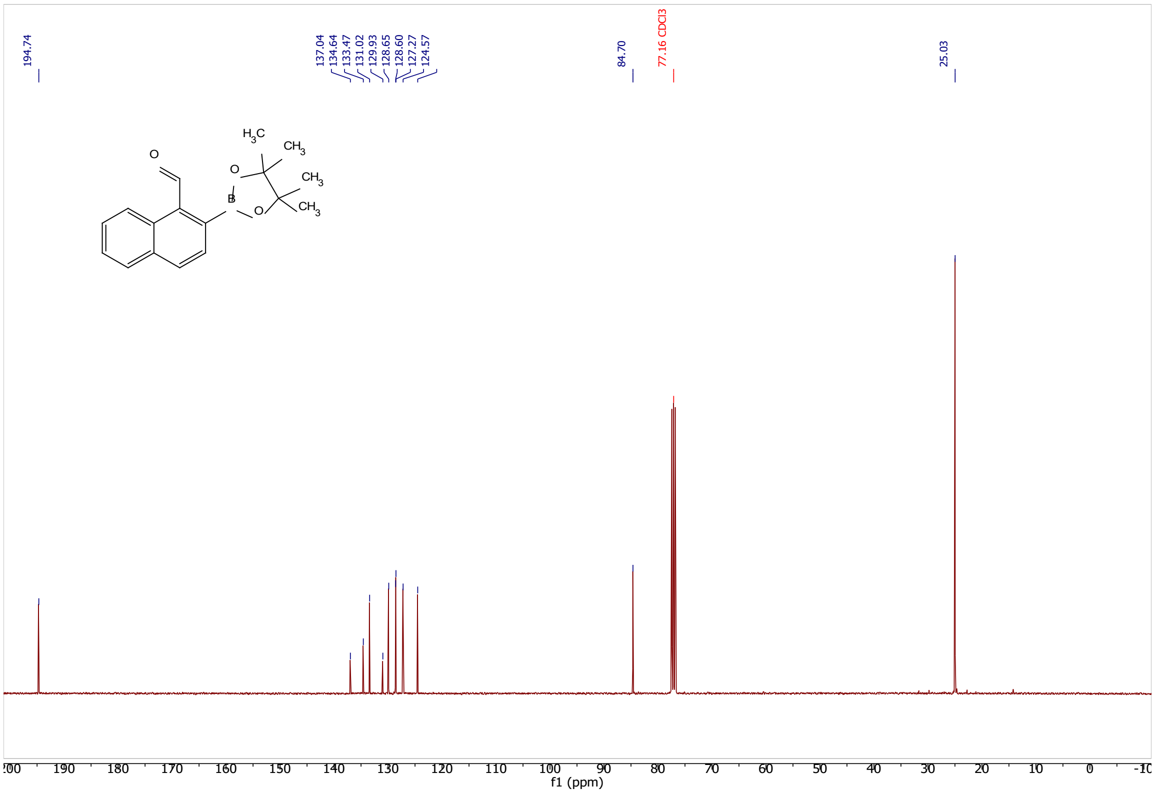

^{1}H NMR spectrum of 2,7-dibromo-9-mesityl-9*H*-fluorene (**17**, 400 MHz, $CDCl_3$)

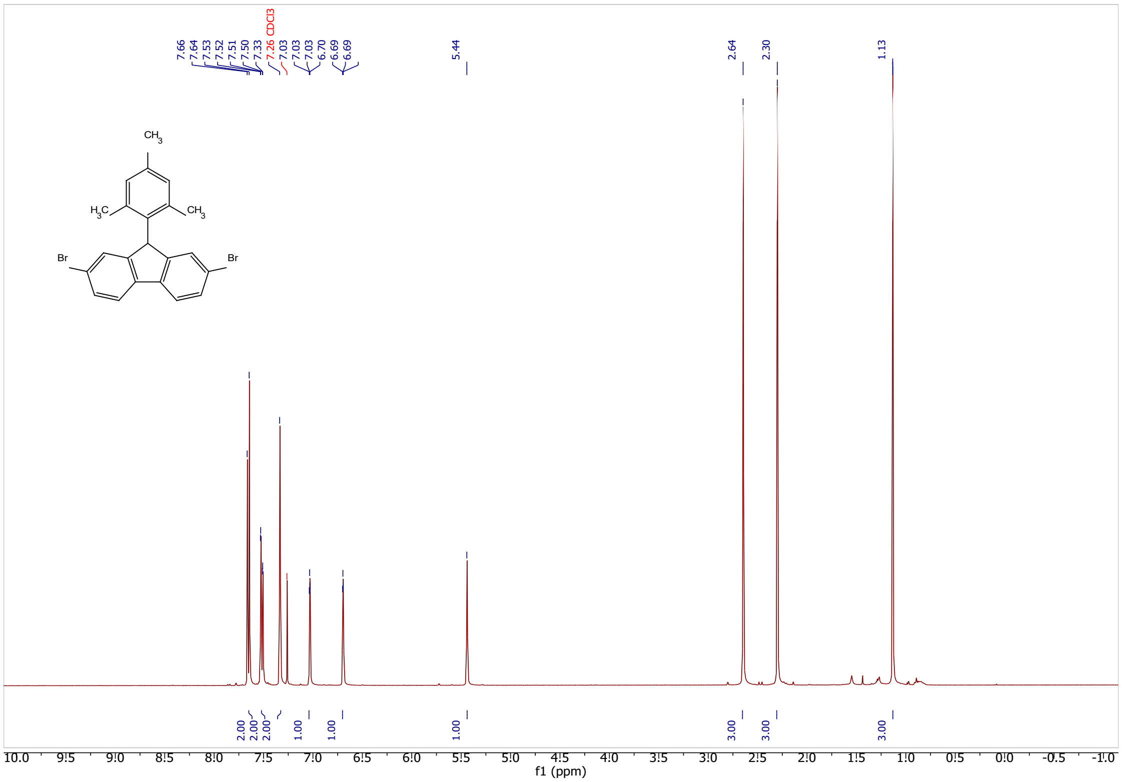

^{13}C NMR spectrum of 2,7-dibromo-9-mesityl-9*H*-fluorene (**17**, 100 MHz, $CDCl_3$)

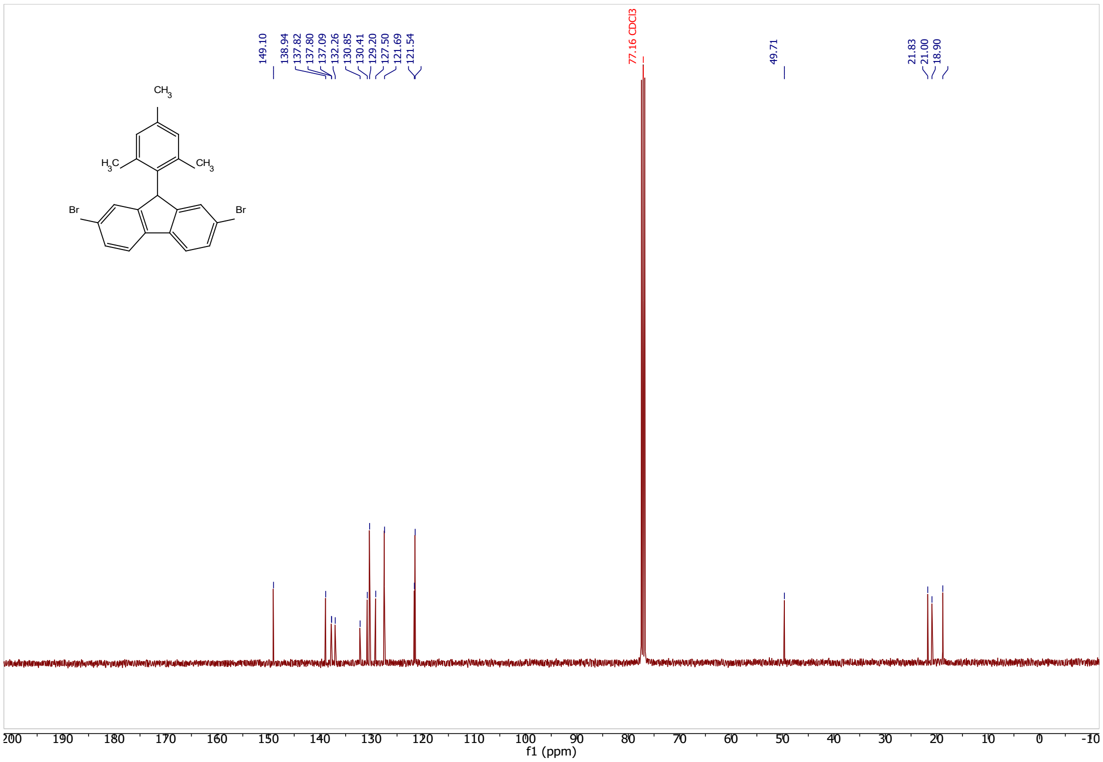

^{1}H NMR spectrum of 1,1'-(9-mesityl-9*H*-fluorene-2,7-diyl)bis(2-naphthaldehyde) (**18**, 400 MHz, $CDCl_3$)

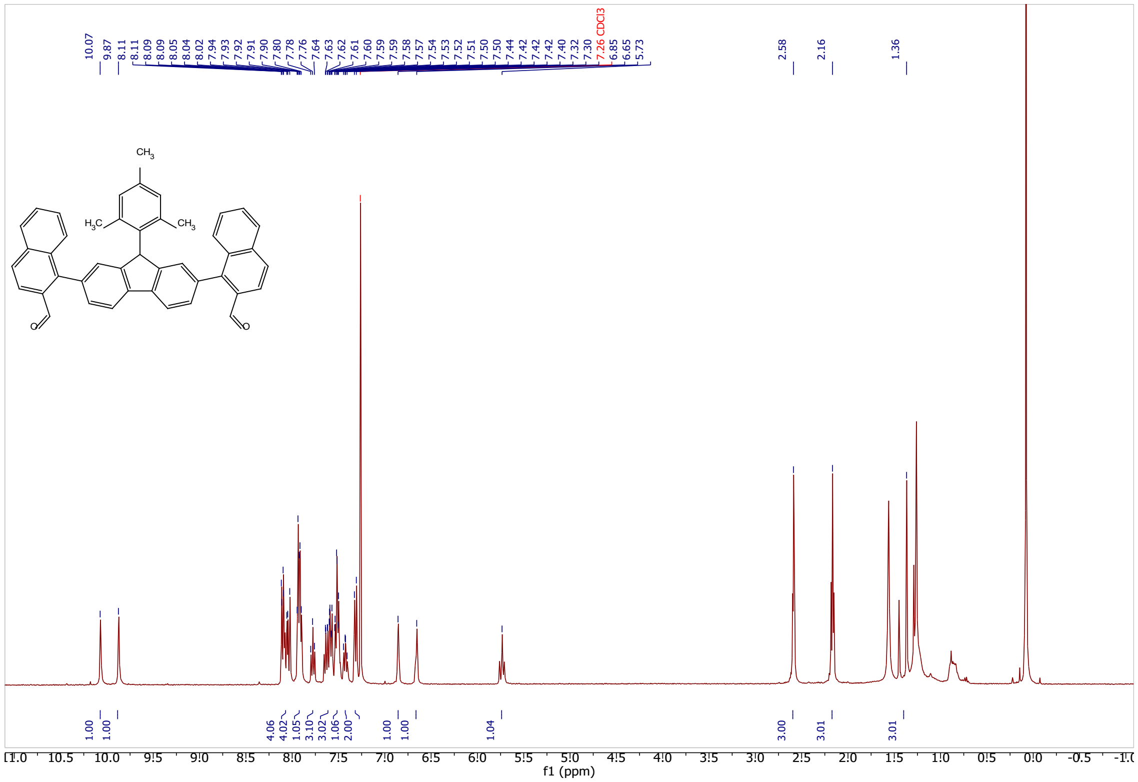

^{13}C NMR spectrum of 1,1'-(9-mesityl-9*H*-fluorene-2,7-diyl)bis(2-naphthaldehyde) (**18**, 100 MHz, $CDCl_3$)

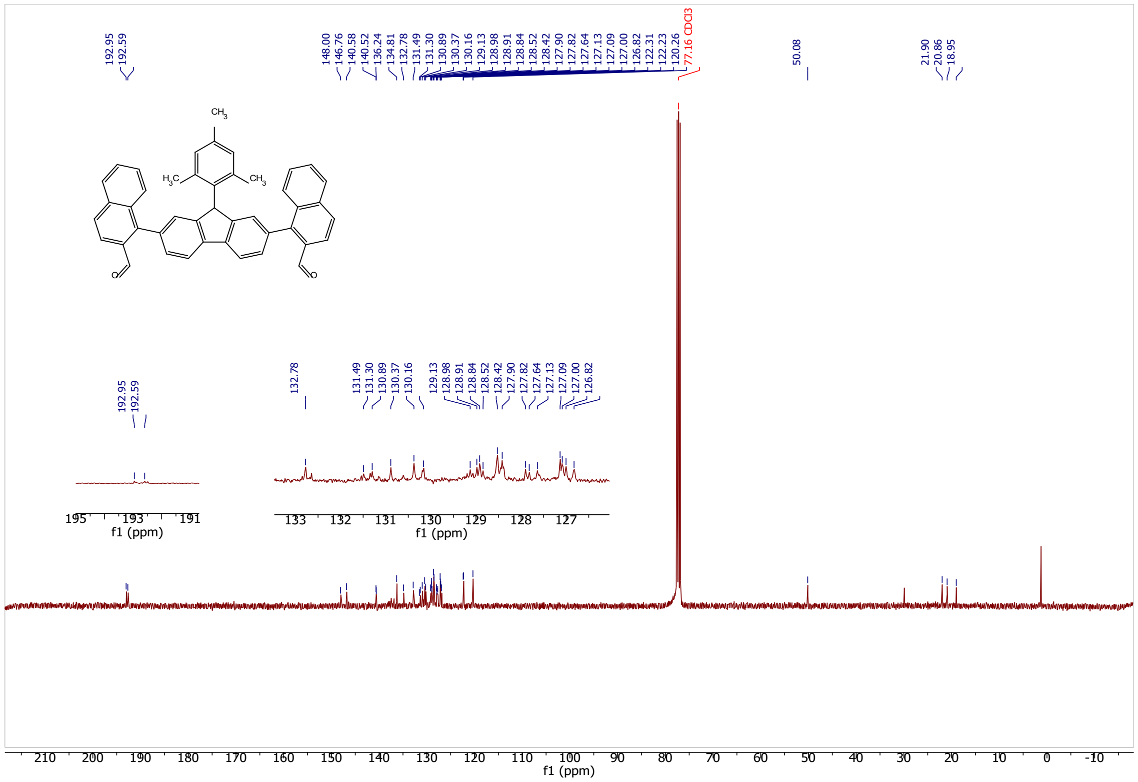

^{1}H NMR spectrum of 2,2'-(9-mesityl-9*H*-fluorene-2,7-diyl)bis(1-naphthaldehyde) (**19**, 400 MHz, $CDCl_3$)

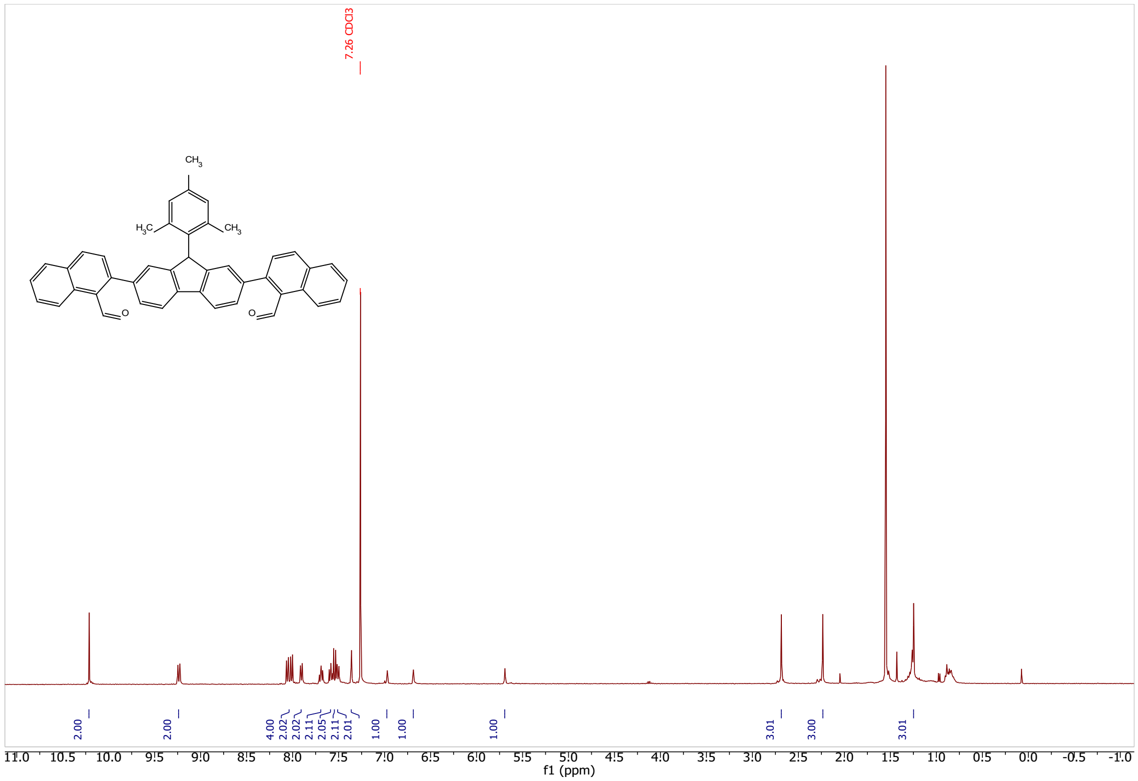

^{13}C NMR spectrum of 2,2'-(9-mesityl-9*H*-fluorene-2,7-diyl)bis(1-naphthaldehyde) (**19**, 100 MHz, $CDCl_3$)

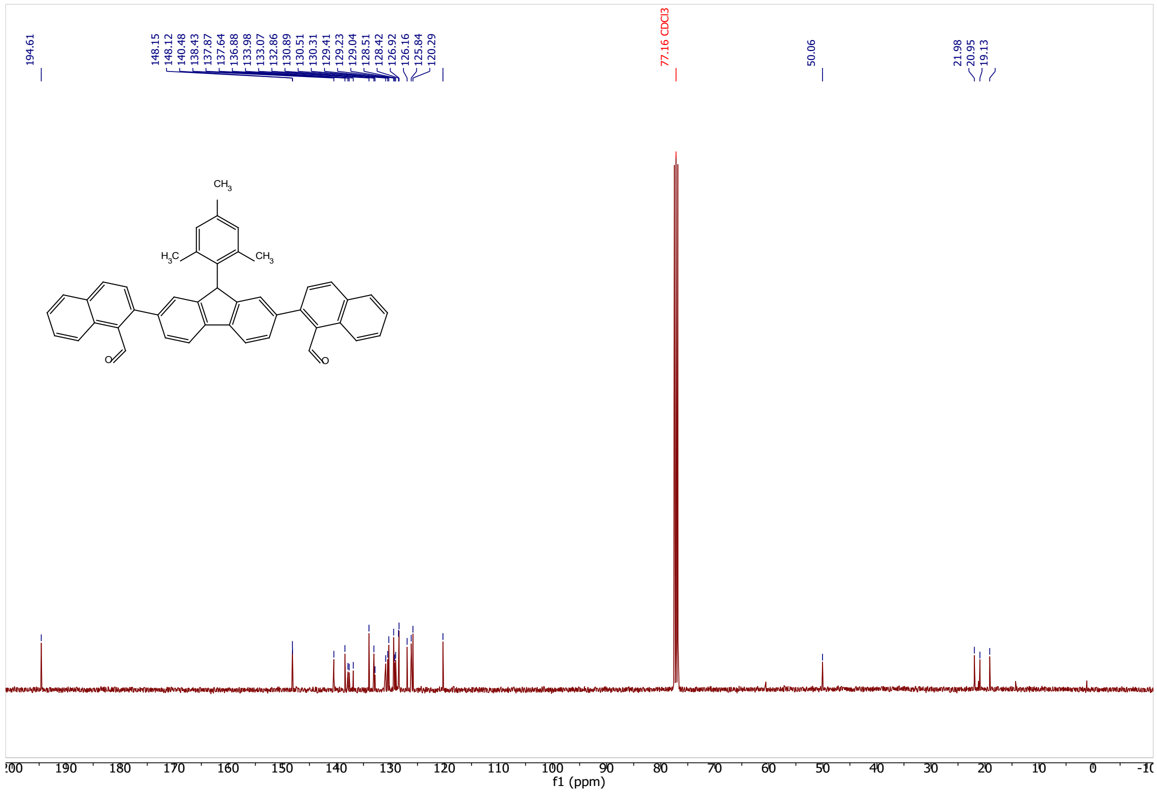

^{1}H NMR spectrum of 10,18-dihydro-7,10,18-trimesityl-7*H*-bisbenz[6,7]indeno[1,2-*b*:2′,1′-*h*]fluorene (**22**, 400 MHz, $CDCl_3$)

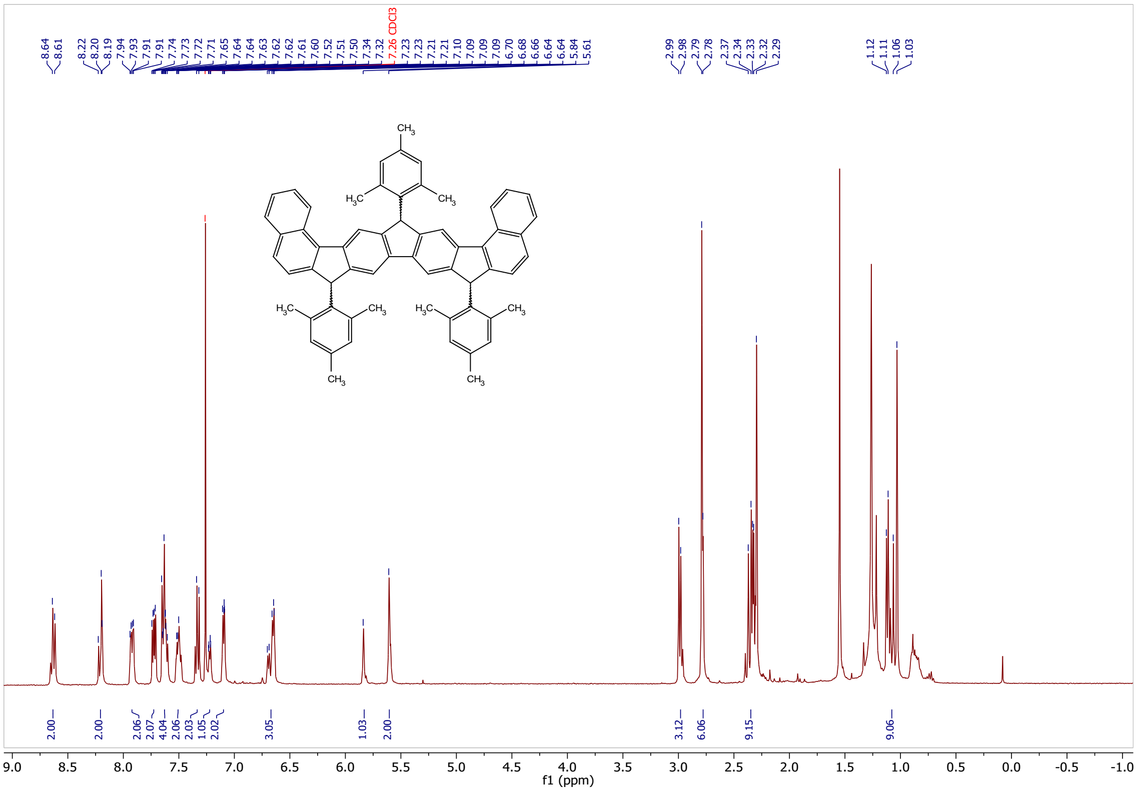

^{13}C NMR spectrum of 10,18-dihydro-7,10,18-trimesityl-7*H*-bisbenz[6,7]indeno[1,2-*b*:2′,1′-*h*]fluorene (**22**, 100 MHz, $CDCl_3$)

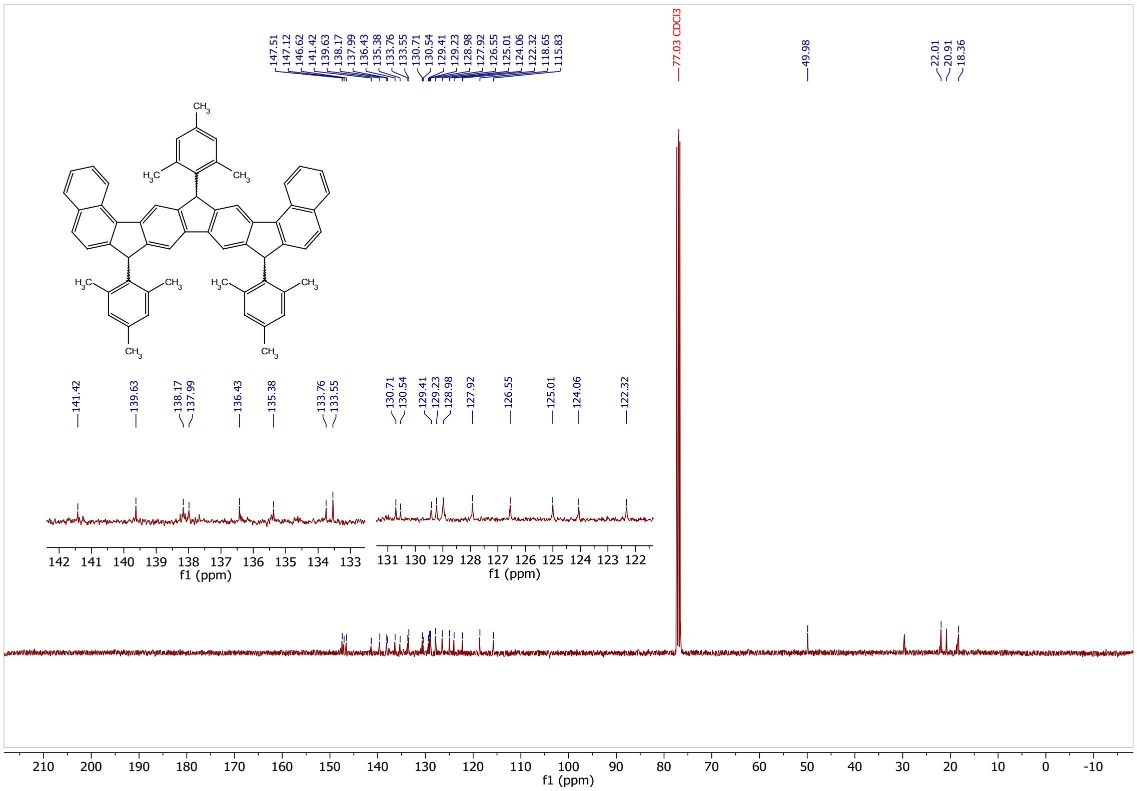

^{1}H NMR spectrum of 16,19-dihydro-8,16,19-trimesityl-8*H*-bisbenz[4,5]indeno[1,2-*b*:2′,1′-*h*]fluorene (**23**, 400 MHz, $CDCl_3$)

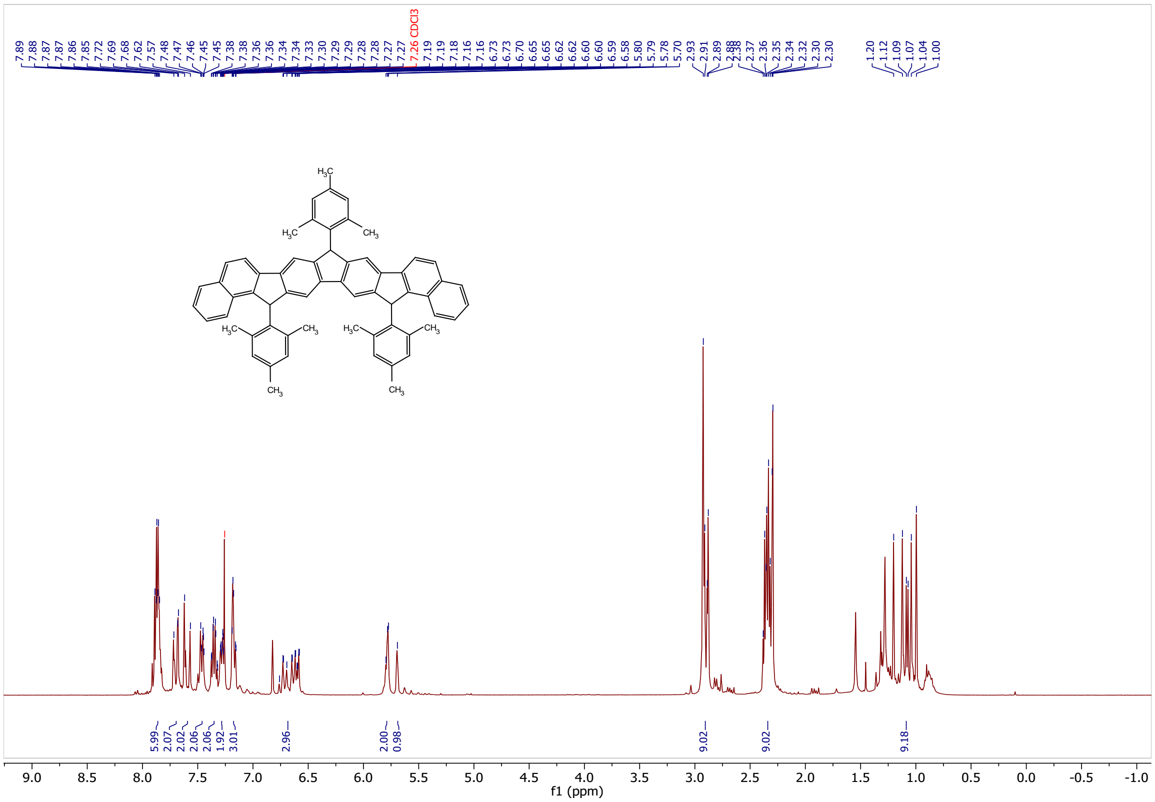

^{13}C NMR spectrum of 16,19-dihydro-8,16,19-trimesityl-8*H*-bisbenz[4,5]indeno[1,2-*b*:2′,1′-*h*]fluorene (**23**, 100 MHz, $CDCl_3$)

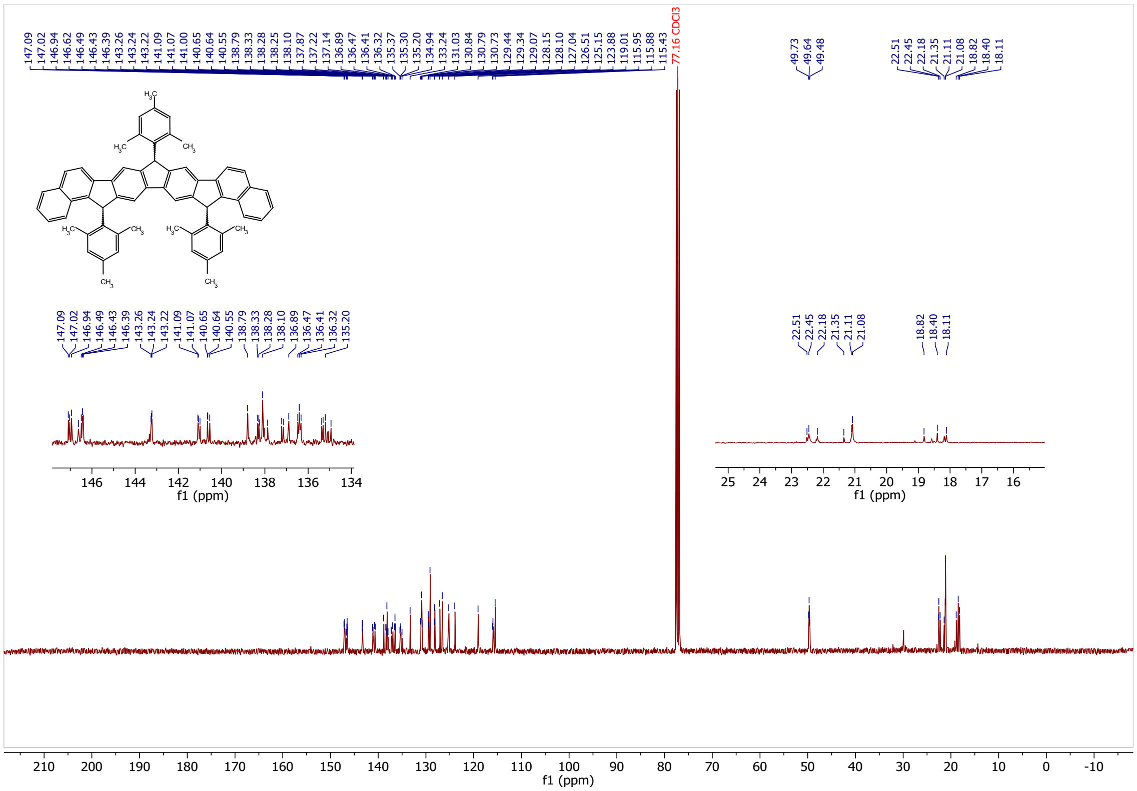

Mass spectrum of 2-(4,4,5,5-tetramethyl-1,3,2-dioxaborolan-2-yl)-1-naphthaldehyde (**15**, ESI)

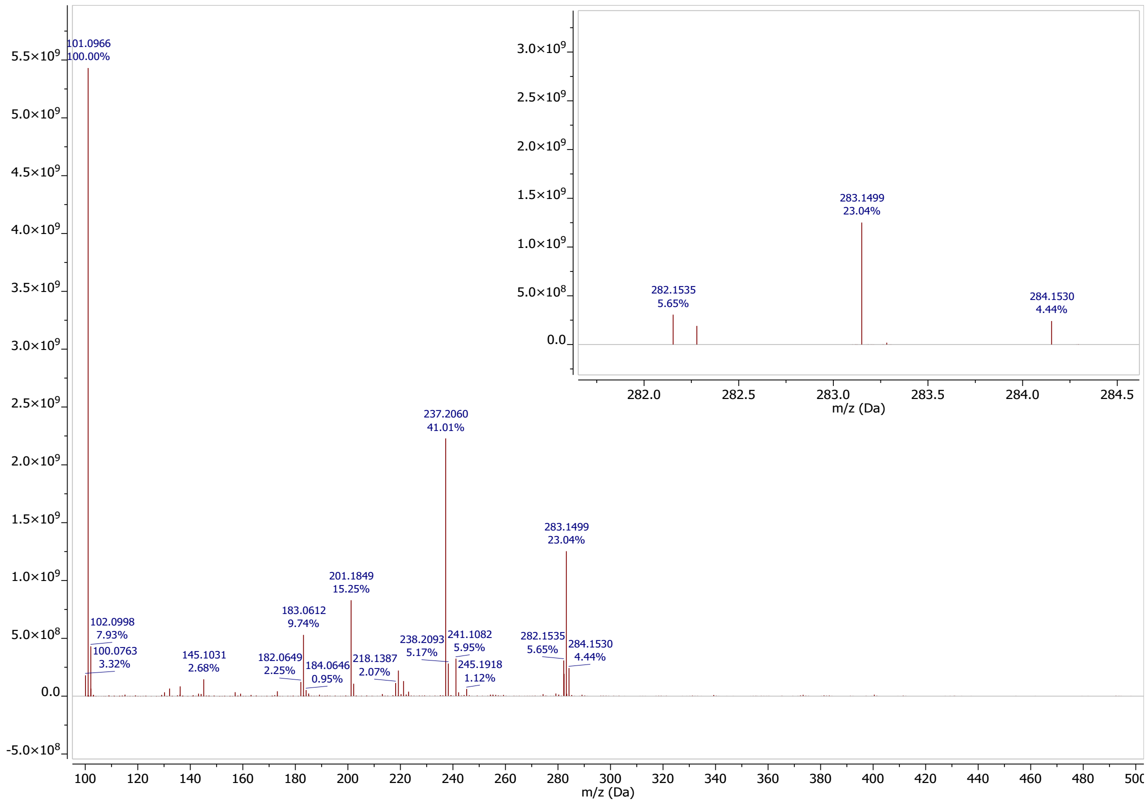

Mass spectrum of 2,7-dibromo-9-mesityl-9*H*-fluorene (**17**, FAB)

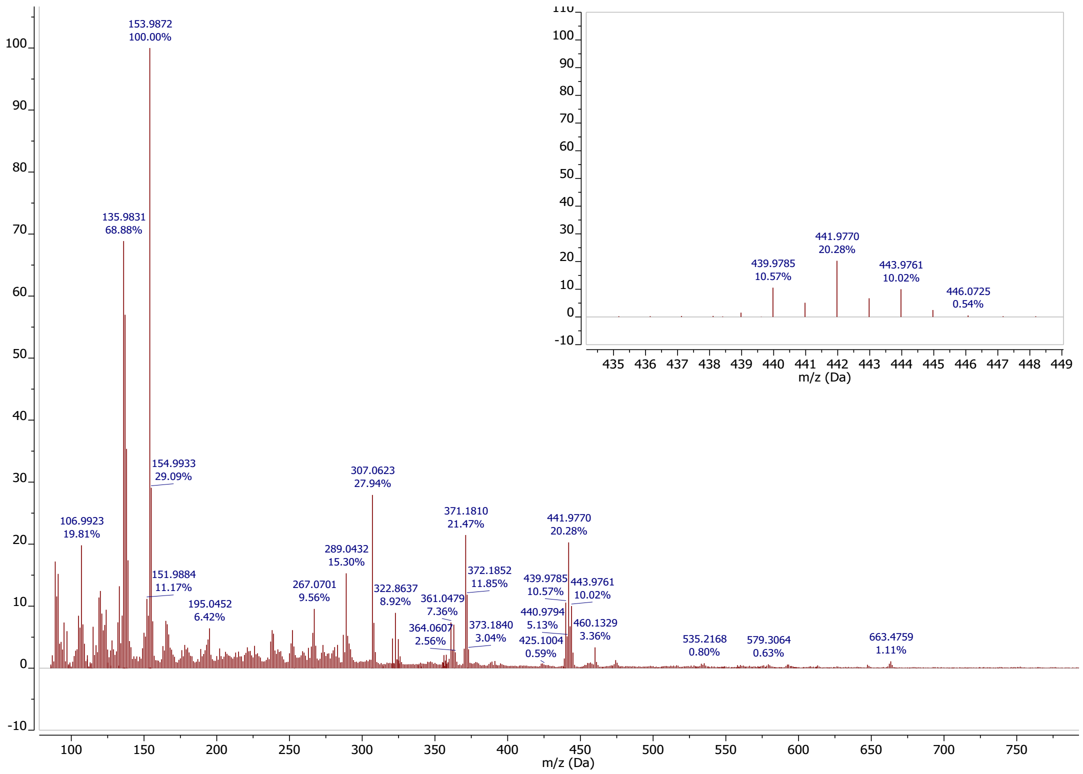

Mass spectrum of 1,1'-(9-mesityl-9*H*-fluorene-2,7-diyl)bis(2-naphthaldehyde) (**18**, ESI)

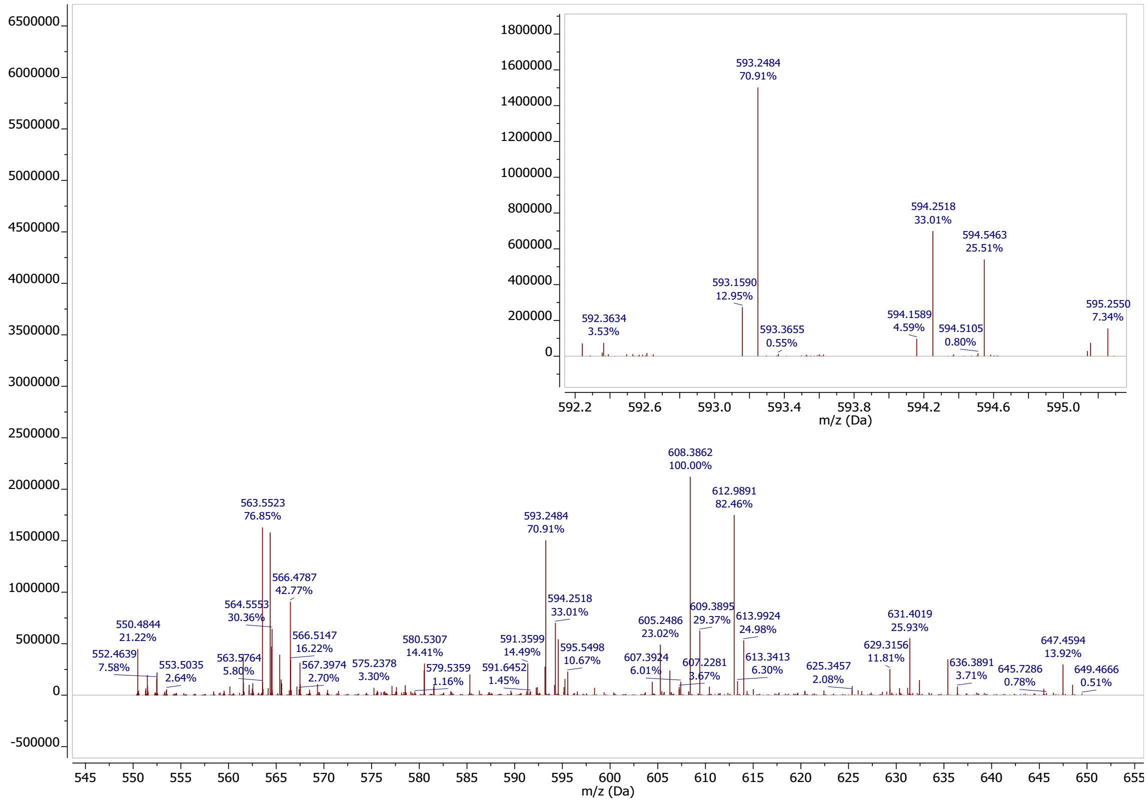

Mass spectrum of 2,2'-(9-mesityl-9*H*-fluorene-2,7-diyl)bis(1-naphthaldehyde) (**19**, ESI)

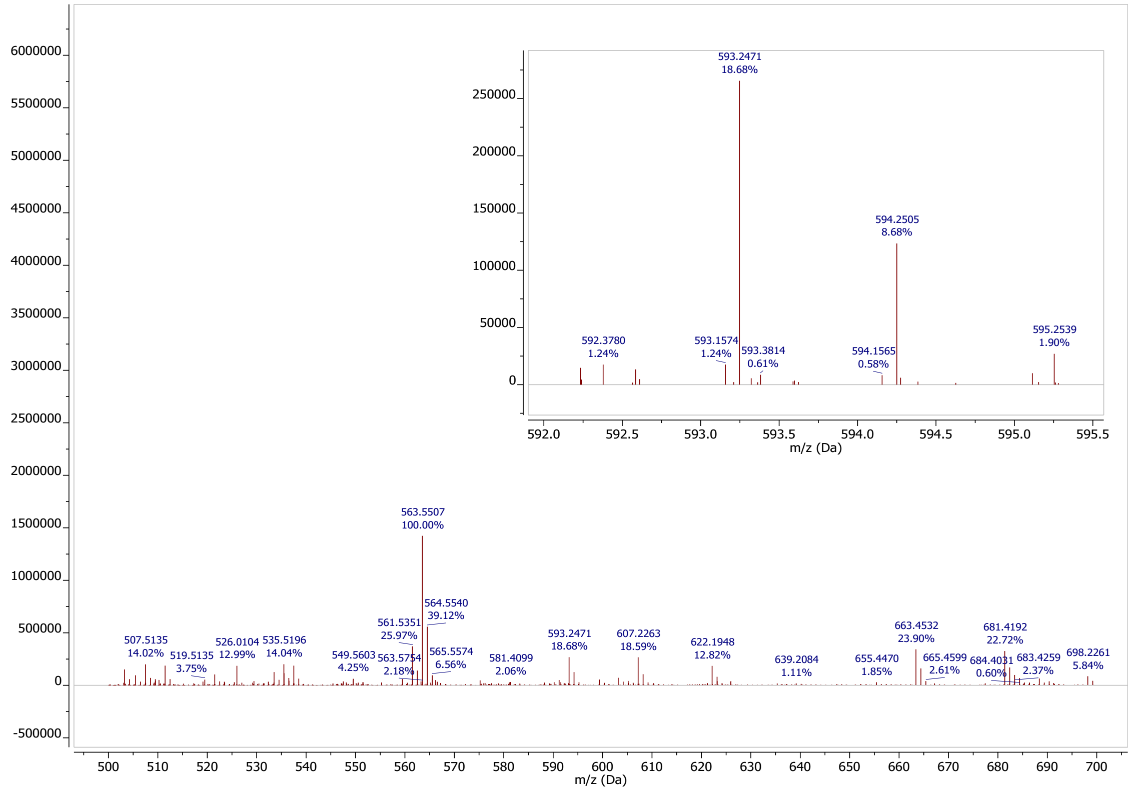

Mass spectrum of 10,18-dihydro-7,10,18-trimesityl-7*H*-bisbenz[6,7]indeno[1,2-*b*:2′,1′-*h*]fluorene (**22**, ESI)

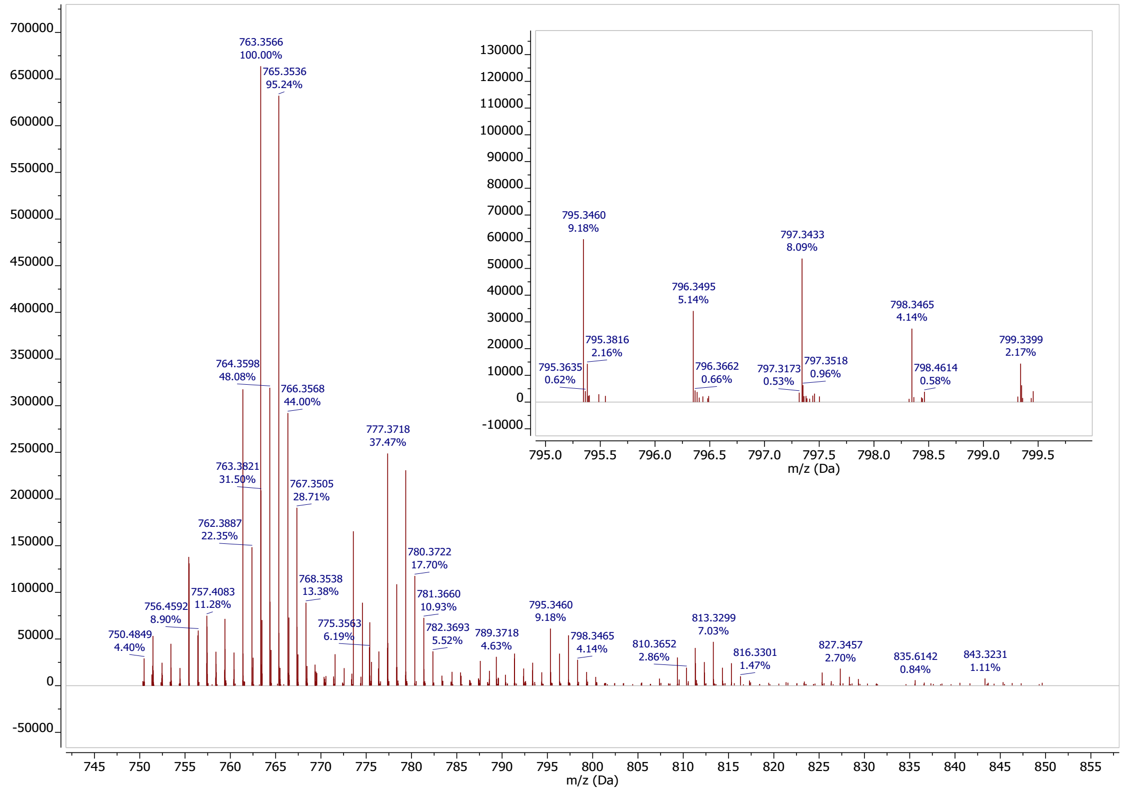

Mass spectrum of 16,19-dihydro-8,16,19-trimesityl-8*H*-bisbenz[4,5]indeno[1,2-*b*:2′,1′-*h*]fluorene (**23**, ESI)

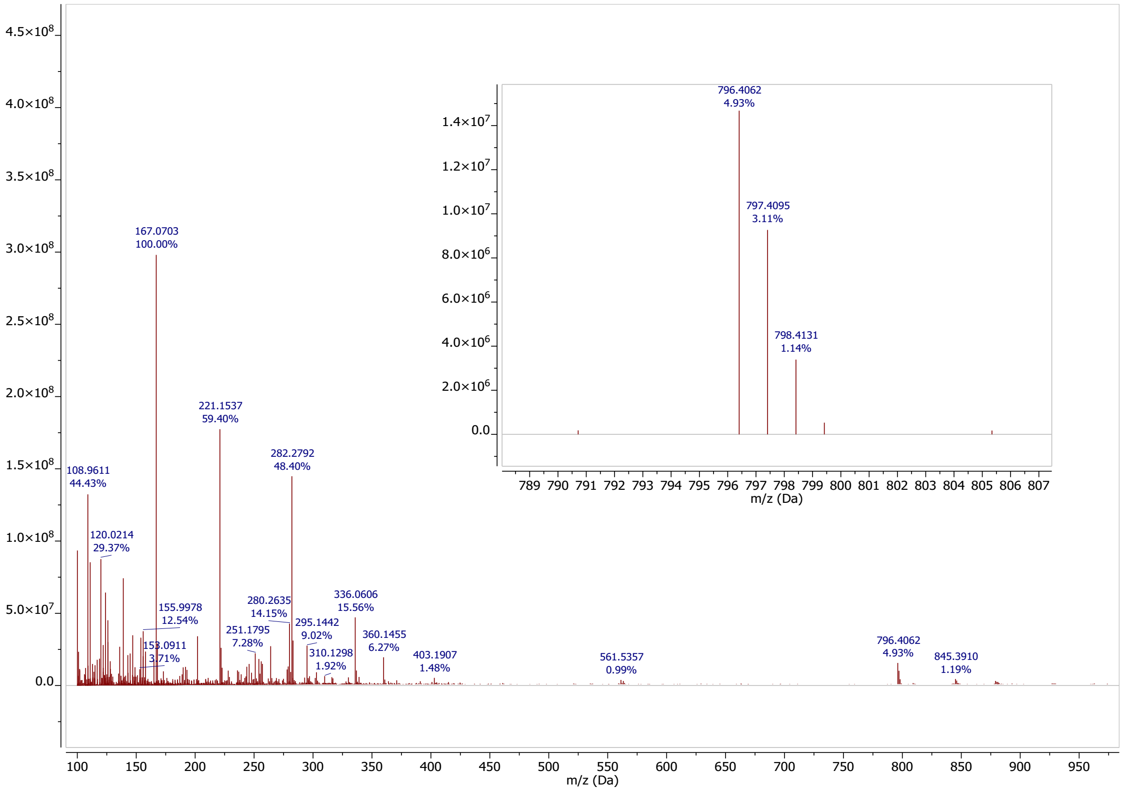

Mass spectrum of radical **24** (ESI)

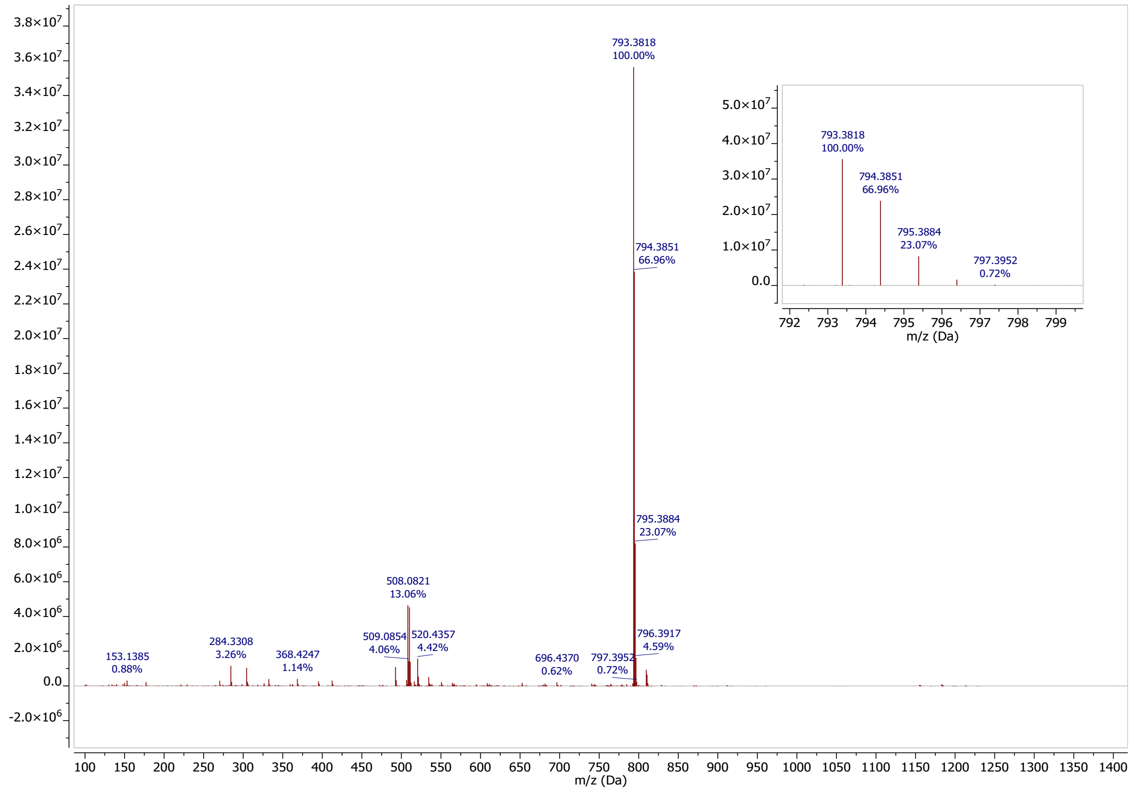

Mass spectrum of radical **25** (ESI)

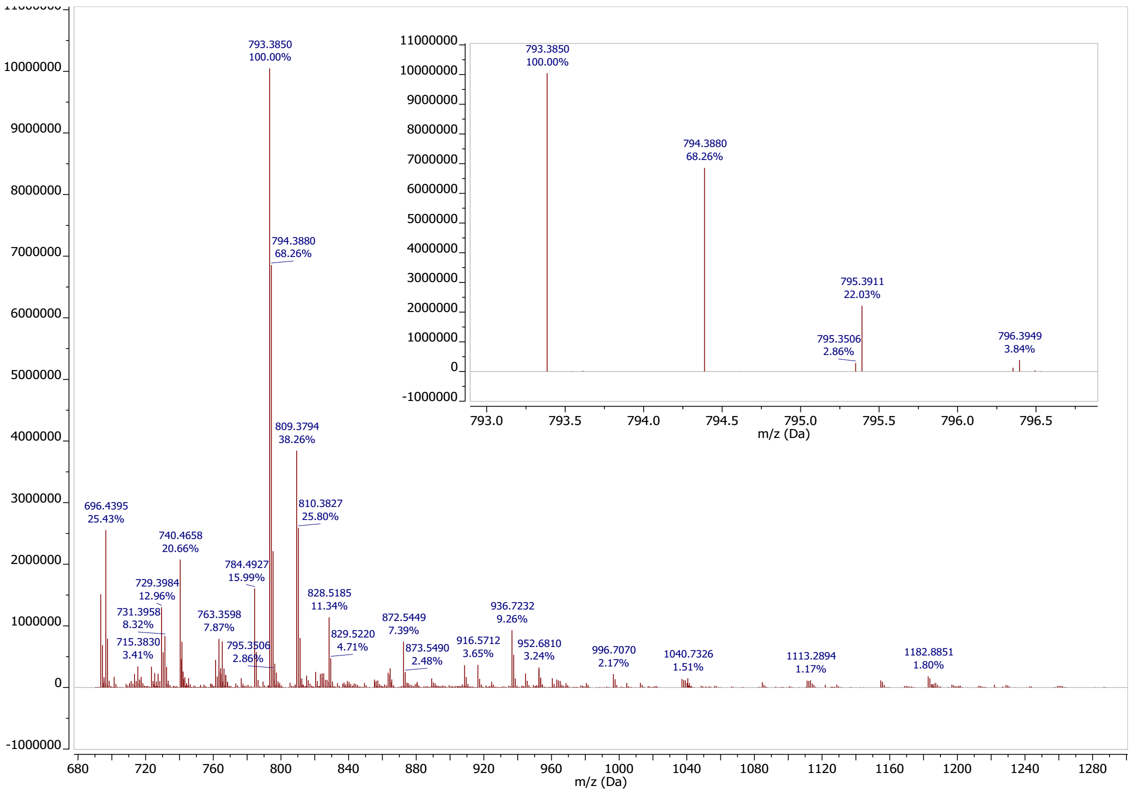

3. DSC/TGA Curves of Radical 24 (top) and Radical 25 (bottom)

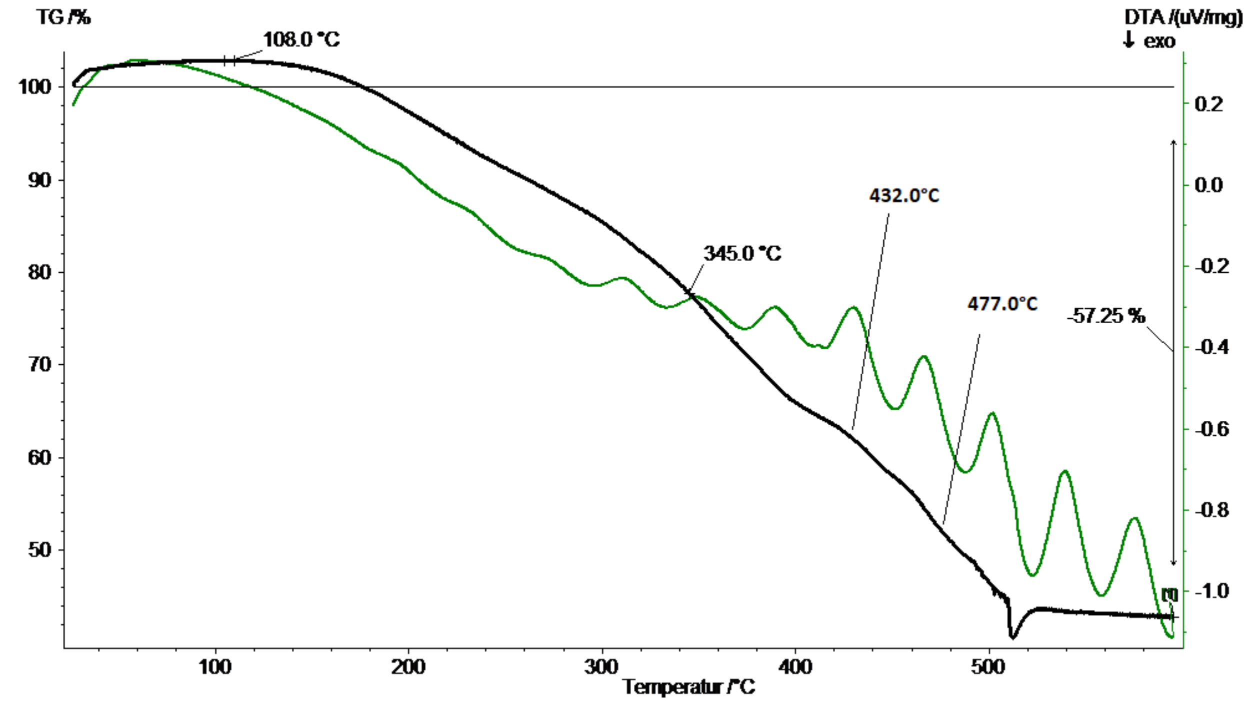

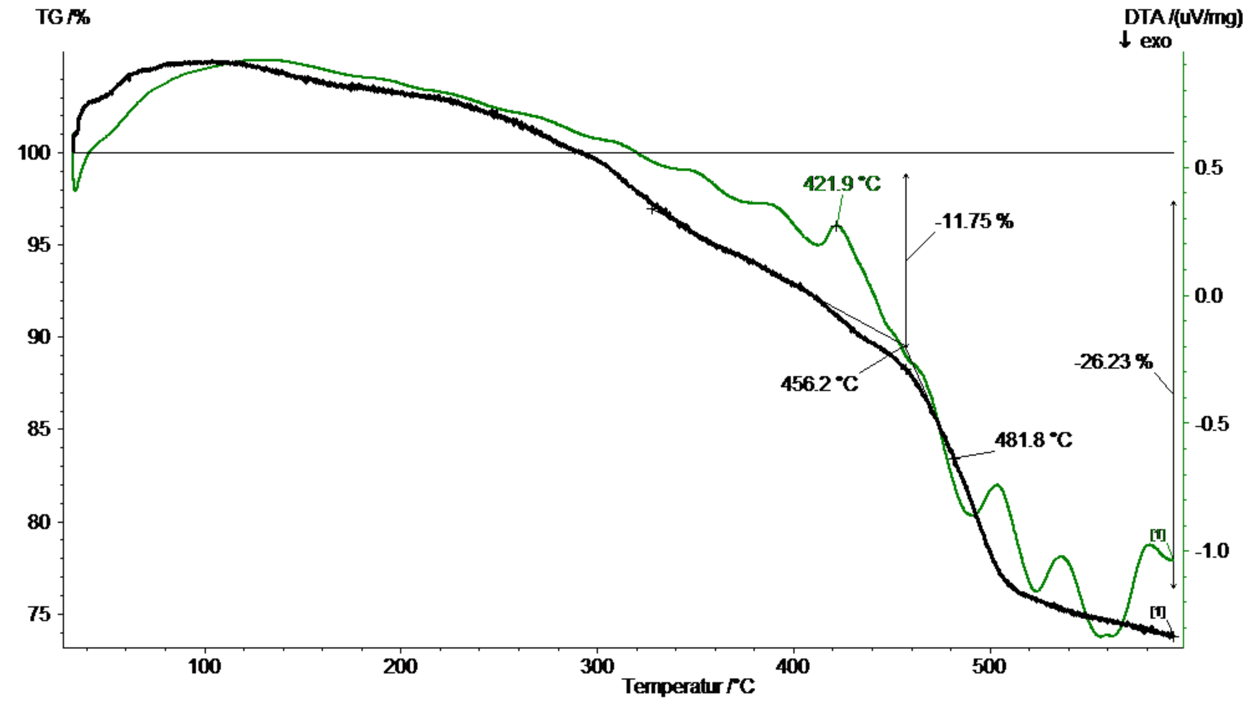

4. UV/Vis Spectra of Radical 24 (top) and Radical 25 (bottom) Recorded in CH_2Cl_2

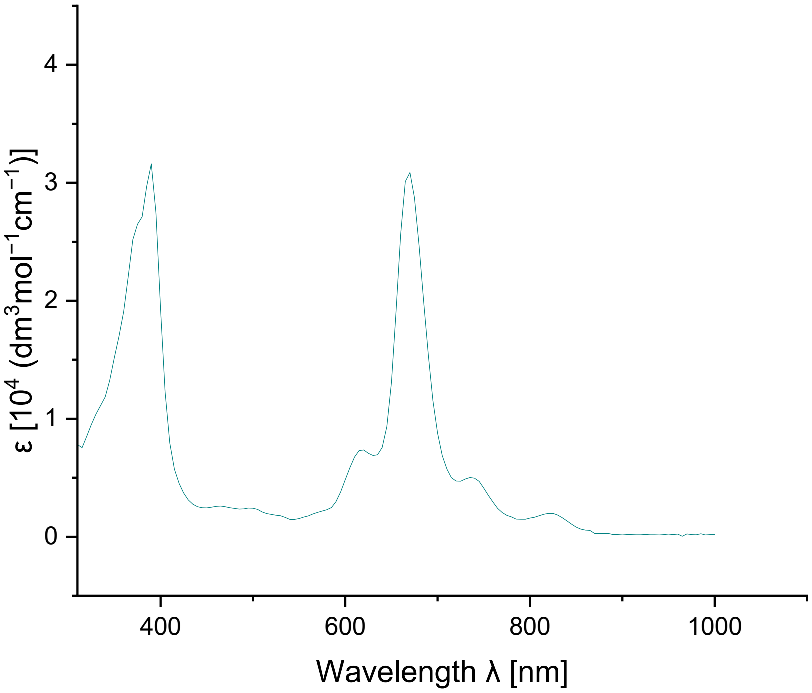

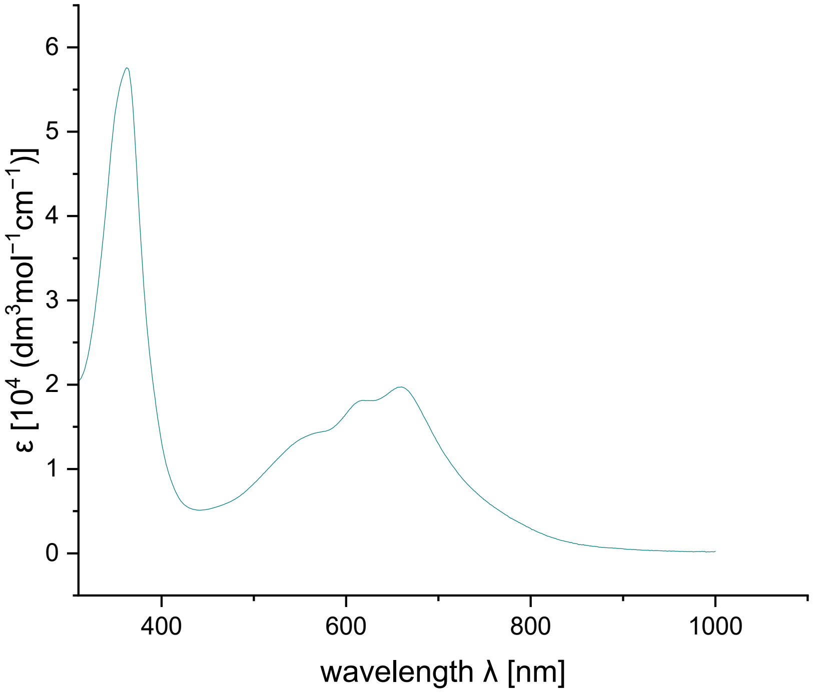

5. EPR Spectra of 24 and 25

Cw X-band [9.4280 GHz (**24**); 9.4279 GHz (**25**)] EPR spectra of radicals **24** and **25** were measured (black) in toluene (0.1 mM) at ambient temperature showing a single resonance signal centered at *g* = 2.0027 (**24**) and *g* = 2.0024 (**25**), respectively. Simulated spectra (red) were obtained for 24 (*S* = ½, no hyperfine couplings, lwpp = 0.95) and 25 (*S* = ½, no hyperfine couplings, lwpp = 0.6). Further details: 1.00 G modulation amplitude, 100 kHz modulation frequency, 1 scan, 2048 points per measurement, 2.00 mW microwave power, 40.96 ms conversion time, 10.48 ms time constant.

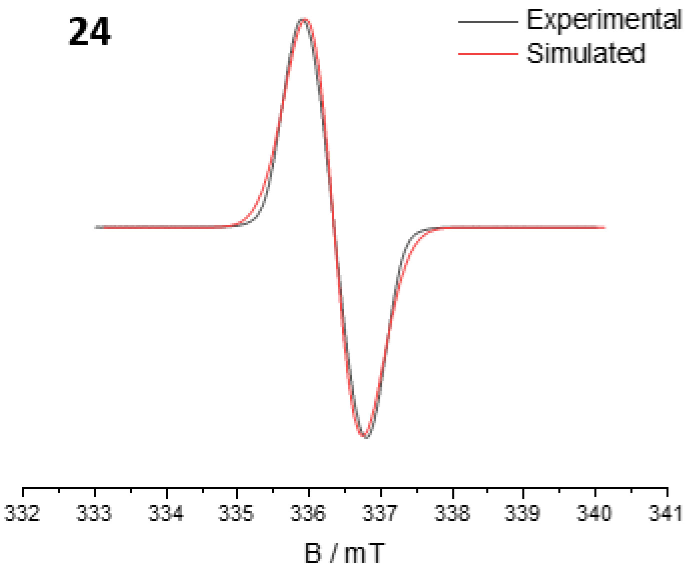

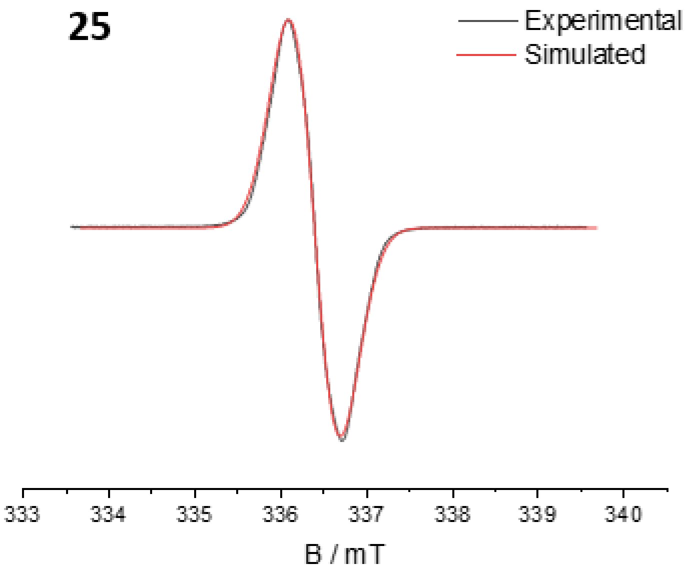

6. Cyclic Voltammograms (CV) of 24 and 25

Half-wave potentials of radicals **24** (top) and **25** (bottom) in CH_2Cl_2 vs. Fc/Fc^+ (internal standard; Fc*, decamethyl ferrocene); conditions: Pt/$[NBu_4][Al(OC_4F_9)_4]$/Ag, v = 100 mV/s.

24

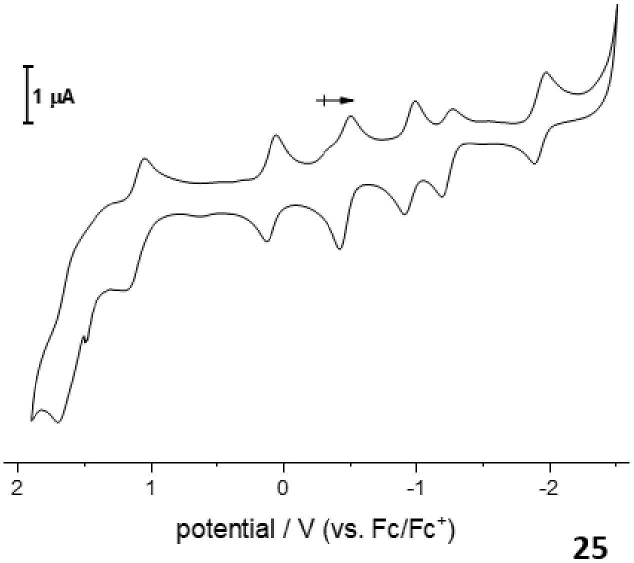

25

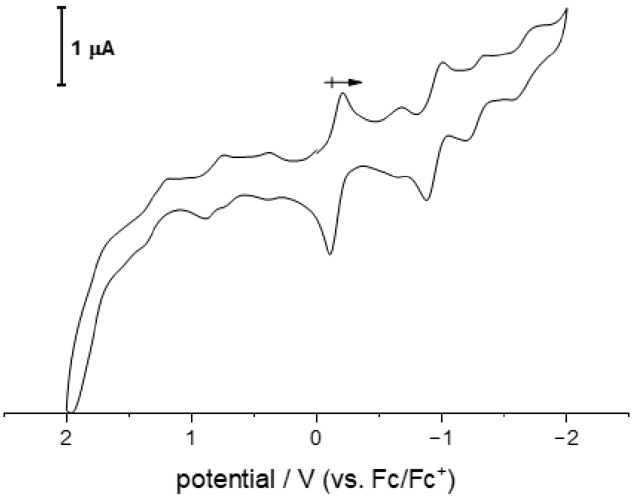

The obtained CV spectra do not allow for a conclusive interpretation. Several attempts were made on our part to clarify the issue, including an evaluation of the compound purity using LC-MS. However, these efforts did not lead to any significant or reliable results. Conducted reactions at different times consistently yielded the same outcome, indicating that the issue is reproducible and not limited to a specific batch. The spectra are here included for the purpose of completeness, but should be taken with caution to avoid misleading interpretations.

7. XRD Data of Precursors 22 and 23

**Compound**	**22**	**23**
CCDC deposition number	2454301	2454300
empirical formula	$C_{69}H_{60}$ (**22**·toluene)	$C_{76}H_{68}$ (**23**·toluene)
formula weight	889.17	981.3
temperature / K	150	150
crystal system	triclinic	monoclinic
space group	$P\bar{1}$	$P2_1/c$
a / Å	15.5622(3)	12.4754(7)
b / Å	19.2581(5)	27.6562(16)
c / Å	19.6678(4)	14.9989(10)
α / °	114	90
β / °	90	92
γ / °	106	90
volume / Å^3	5120.7(2)	5170.9(5)
Z	4	4
P_{calc} / g/cm^3	1.153	1.261
μ / mm^{-1}	0.27	0.27
F(000)	1896.0	2096.0
crystal size / mm^3	0.15 × 0.09 × 0.00	0.15 × 0.117 × 0.10
radiation	Ga-Kα (λ = 1.34143 Å)	Ga-Kα (λ = 1.34143 Å)
$2\Theta_{min}$ – $2\Theta_{max}$ / °	4.314 to 119.996	6.168 to 124.994
reflections collected	79575	43090
independent reflections	22795	12229
R_{int}	0.0405	0.0357
R_{sigma}	0.0386	0.0346
data/restraints/parameters	22795/24/1220	12229/0/568
GooF	1.071	1.075
R_1	0.0521	0.0484
wR_2	0.1421	0.1473
R_1(all)	0.0791	0.0726
wR_2(all)	0.1553	0.1606
ρ_{e-} max/ρ_{e-} min / e Å^{-3}	0.18/–0.23	0.28/–0.22

Solid-state structures of all-*cis*-22

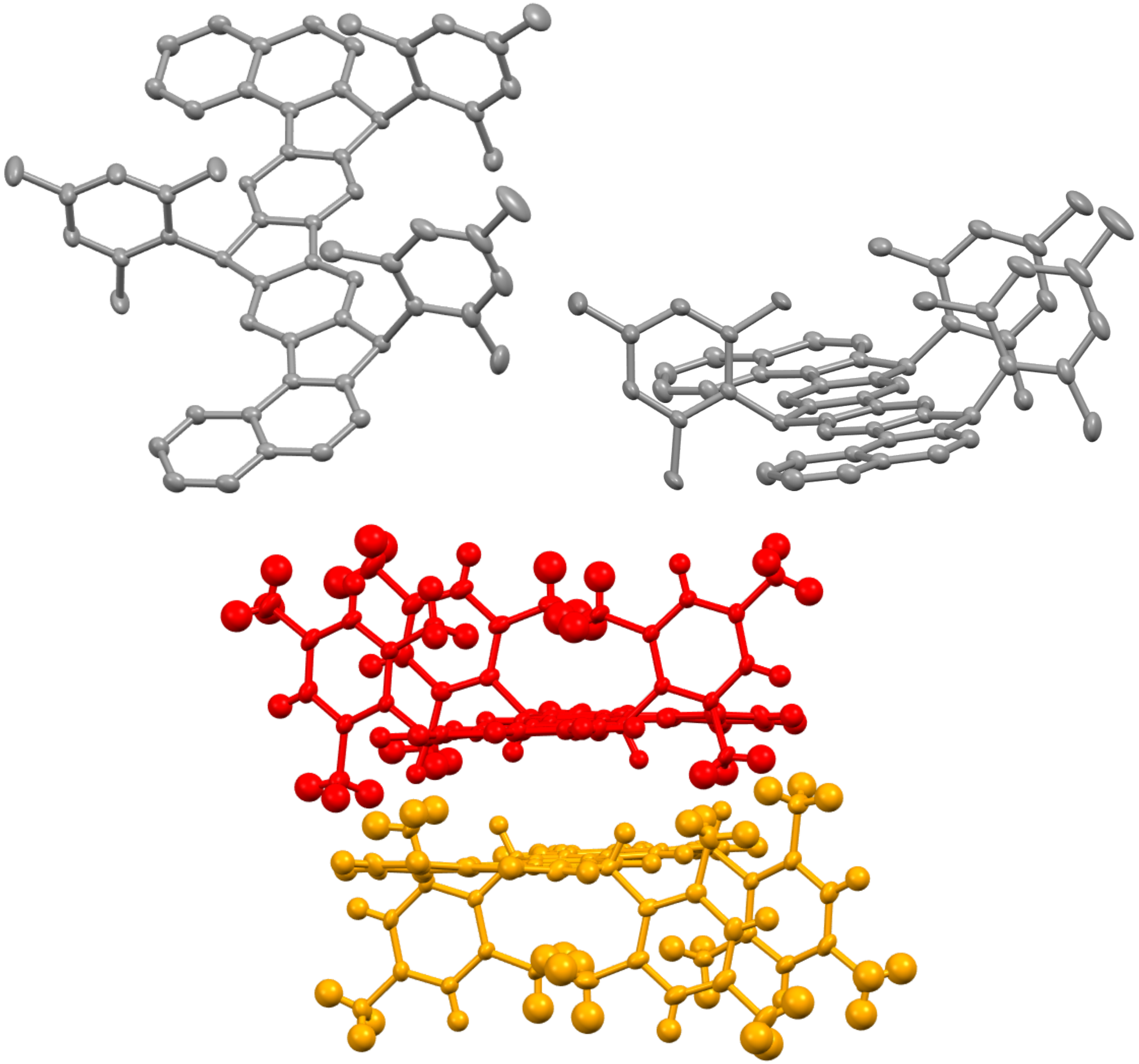

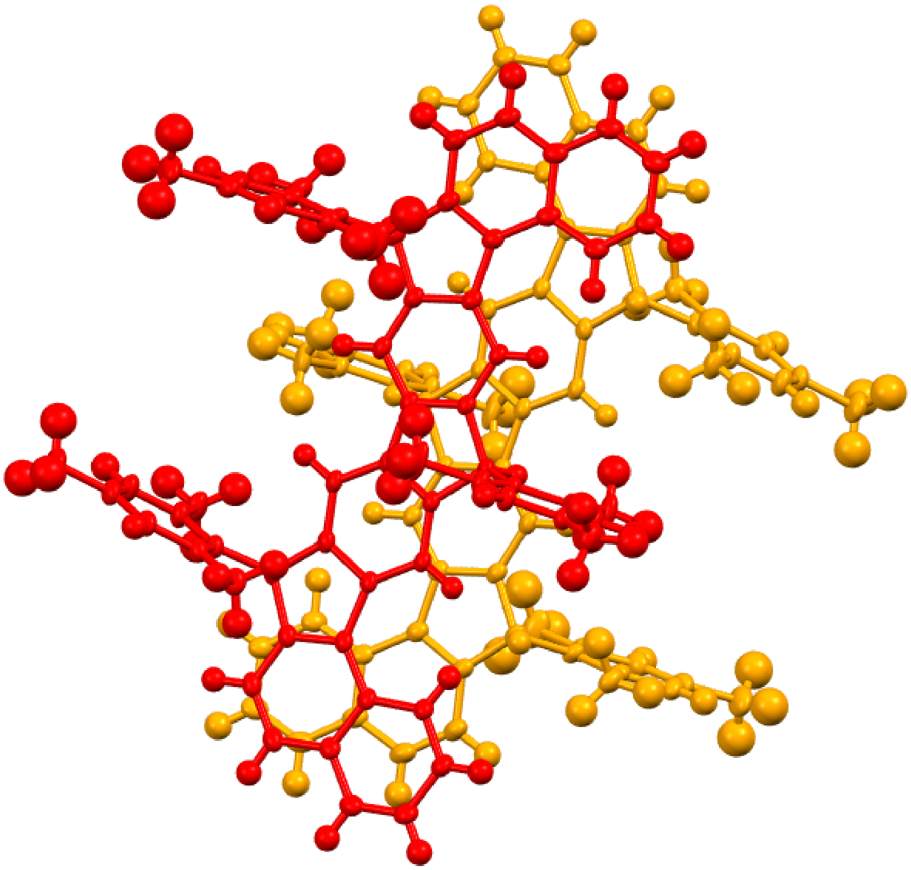

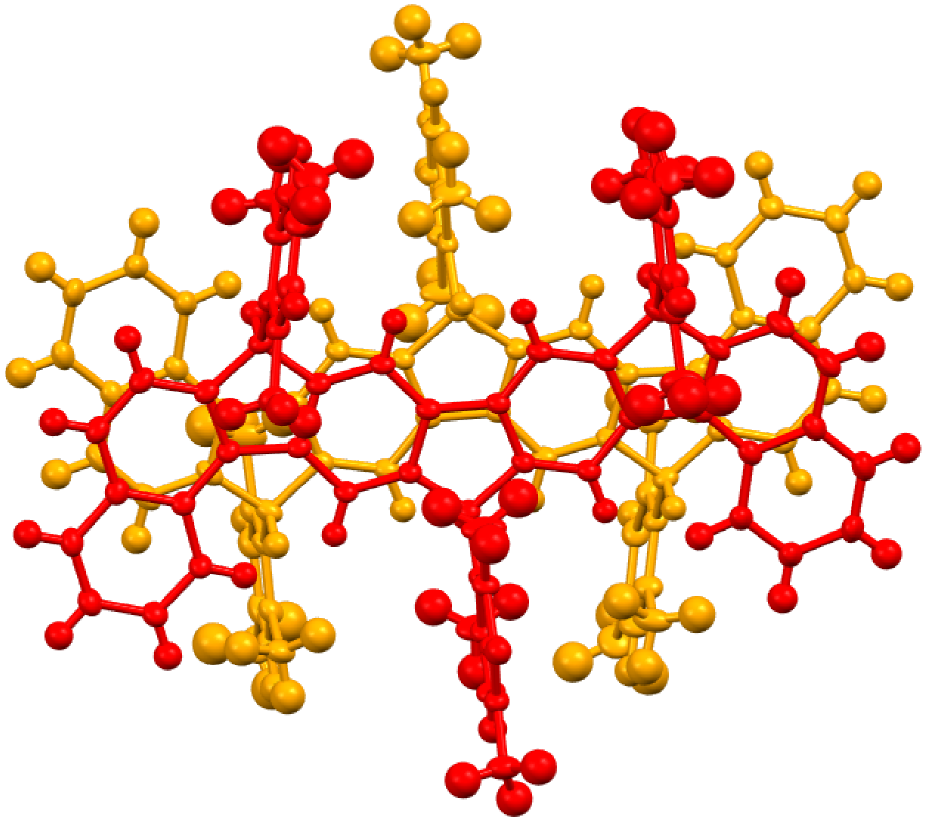

Solid-state structures of *cis*,*trans*-23

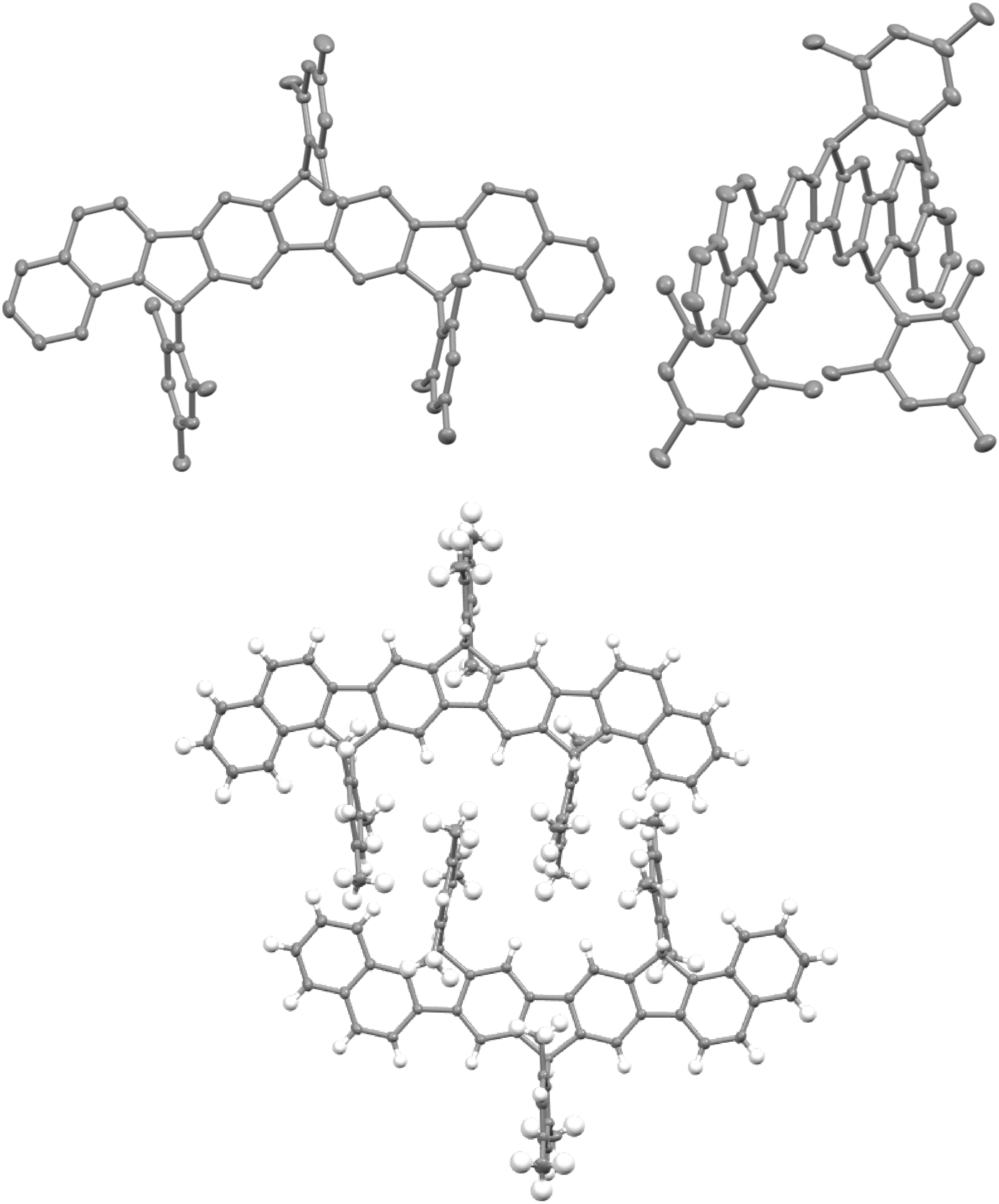

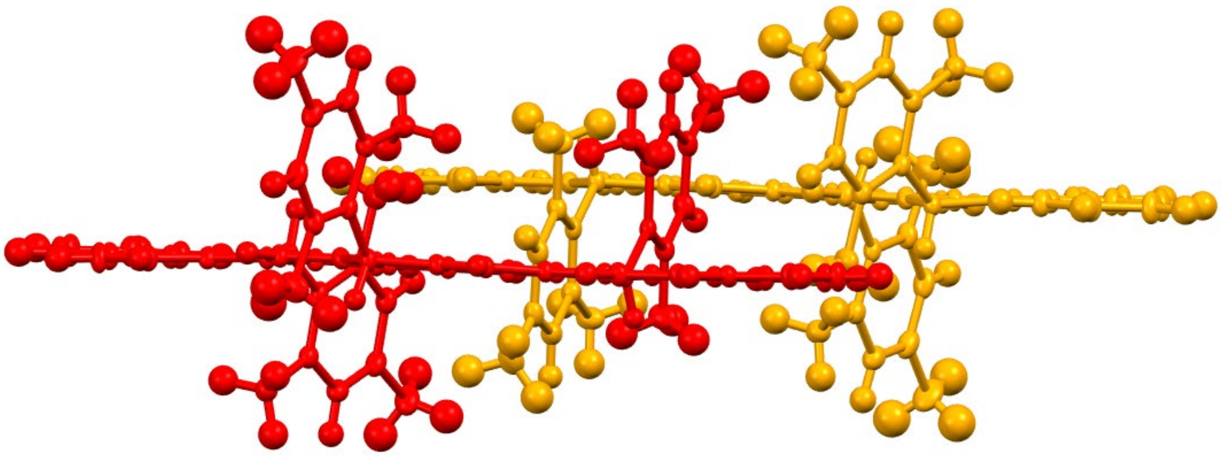

8. Computational Details

Radical **24** and **25** were optimized at the upbe0[83–85]/def2-TZVP[86–87] level with Grimme's dispersion correction at the D3 level[88] and with Becke-Johnson damping (GD3BJ)[89] by using the Gaussian16[90] software package. Except for the frequency analysis, all calculations were performed using with the thus obtained geometries; all calculations were performed at this level (unless otherwise noted). Optimization and frequency analysis[91–93] at the uM06[94]/6-311++g(d,p)[95–100] level verified that the structure is at a local minimum (no imaginary frequencies). IR spectra of the radicals were visualized with GaussSum[101] [full width at half maximum (FWHM) of 10 cm^{-1}] using a scaling factor of 0.9619.[102] UV/Vis/NIR spectra of the radicals were calculated with a time-dependent DFT calculation [td=(nstates=50)][103–105] applying a modelled solvent field of methylene chloride with the cpcm-scrf method[106–108] and visualized with GaussSum [full width at half maximum (FWHM) of 800 cm^{-1}]. $NICS(1)_{zz}$ and $NICS(1)_{\pi zz}$ values[66] of radical **24** and **25** were calculated at the uB3LYP[109–110]/6-311g(d,p) level with the keyword nmr=giao. For the calculation of $NICS(1)_{\pi zz}$ values the program py.Aroma was used.[111–112] Hyperfine coupling constants (EPR) and *g* values were calculated with the keywords nmr=giao[113–116] and prop=epr.[117–119] Frontier orbitals and spin densities were determined by single-point energy calculation and visualized with GaussView.[120] Output files for ACID calculations[71] were obtained using the keyword nmr=csgt, they were performed with AICD 3.04 at the uB3LYP/6-311g(d,p) level. ACID plots were visualized with the image rendering software POV-Ray.[121]

9. Archive Entries for Single-Point Energy Calculations on a Minimum Structure for Radicals 24 and 25

Archive entry for radical **24**


```
1\1\GINC-N1315\SP\UPBE1PBE\def2TZVP\C62H49(2)\KA_LM9425\11-Oct-2024\0\
\# sp upbe1pbe/def2tzvp geom=connectivity empiricaldispersion=gd3bj\\T
itle Card Required\\0,2\C\C,1,1.45652153\C,2,1.38401605,1,119.37328339
\C,3,1.41436747,2,118.69397981,1,0.6893401,0\C,4,1.44550561,3,121.5934
0609,2,-0.46956923,0\C,5,1.3810952,4,120.24046722,3,0.3323582,0\H,3,1.
08996999,2,122.87904884,1,-179.10813966,0\H,6,1.09308082,5,121.9916869
5,4,179.43686764,0\C,4,2.30249972,3,165.40901815,2,-179.04665741,0\C,9
,1.41436335,4,165.40264585,3,-2.70946629,0\C,10,1.38400186,9,118.69262
858,4,-178.89733215,0\C,11,1.45651663,10,119.37388051,9,0.67256282,0\C
,12,1.41394801,11,121.87390063,10,-0.77912968,0\C,13,1.38111212,12,118
.21882729,11,0.64815399,0\H,10,1.08996819,9,118.42762225,4,0.91112805,
0\H,13,1.09307877,12,119.78882362,11,-179.21088938,0\C,11,1.4653511,10
,133.80884757,9,-179.31752488,0\C,17,1.42295898,11,106.99998377,10,-17
9.99403811,0\C,12,1.41141606,11,108.98727063,10,179.93481811,0\C,2,1.4
6535324,1,106.81566395,6,179.20188881,0\C,1,1.41139166,6,129.13252,5,1
79.80012727,0\C,20,1.42295779,2,107.00026835,1,0.0358954,0\C,17,1.4210
9988,11,133.11375997,10,-0.24323313,0\C,18,1.41296764,17,121.63590292,
11,178.77529415,0\C,23,1.4458463,17,117.72436166,11,-179.30518428,0\C,
24,1.37606252,18,119.05022676,17,0.90586047,0\C,20,1.42110125,2,133.11
560542,1,179.79302576,0\C,22,1.41296271,20,121.63682533,2,178.7588964,
0\C,27,1.44584582,20,117.72589085,2,-179.28239575,0\C,28,1.37606525,22
,119.05074661,20,0.90494734,0\H,28,1.09200168,22,119.76209024,20,-178.
75412159,0\H,24,1.09200147,18,119.7625456,17,-178.75414243,0\C,4,1.422
35593,3,129.44847313,2,179.99486045,0\C,33,1.47502108,4,125.96359968,3
,-0.42208428,0\C,34,1.41255695,33,119.96477808,4,106.48289125,0\C,34,1
.41259644,33,119.94336733,4,-73.81376726,0\C,35,1.3985758,34,118.93556
94,33,179.93731019,0\C,36,1.39856254,34,118.93755304,33,-179.6579076,0
\C,37,1.39851511,35,121.99727552,34,-0.1862313,0\H,37,1.09367551,35,11
8.71657192,34,179.55902376,0\H,38,1.09366318,36,118.71761513,34,179.06
981032,0\C,19,1.47238196,12,126.50796708,11,-179.65687841,0\C,42,1.413
```

51146,19,119.90502125,12,-107.83747696,0\C,42,1.4132084,19,120.1194654
4,12,71.82590793,0\C,43,1.39806681,42,119.01955494,19,179.56488299,0\C
,44,1.39849189,42,118.96642376,19,-179.88803761,0\C,46,1.39842054,44,1
22.02954921,42,0.22412437,0\H,45,1.09372136,43,118.73041543,42,-178.96
523846,0\H,46,1.09371709,44,118.70948544,42,-179.4543969,0\C,21,1.4723
8886,1,126.50927508,6,1.12902896,0\C,50,1.4135039,21,119.90574913,1,-1
07.82402827,0\C,50,1.41320597,21,120.11899957,1,71.84102736,0\C,51,1.3
980679,50,119.02024224,21,179.56728345,0\C,52,1.3984916,50,118.9663902
8,21,-179.8884142,0\C,54,1.3984229,52,122.02980251,50,0.22200233,0\H,5
3,1.09372093,51,118.73010407,50,-178.96894029,0\H,54,1.09371724,52,118
.70910817,50,-179.45846915,0\C,36,1.50634309,34,120.88789446,33,-1.018
19097,0\H,58,1.10009835,36,111.30893594,34,-49.41983862,0\H,58,1.10073
701,36,111.6805578,34,69.41522535,0\H,58,1.09777889,36,110.80717718,34
,-170.19839531,0\C,35,1.50635745,34,120.90550456,33,-1.03366393,0\H,62
,1.10006438,35,111.28523507,34,-49.21186529,0\H,62,1.10075281,35,111.7
0843674,34,69.62724703,0\H,62,1.09778106,35,110.80609954,34,-169.96871
362,0\C,39,1.50530383,37,120.97110991,35,178.66925546,0\H,66,1.1021219
8,39,110.95222507,37,-89.40117866,0\H,66,1.09885956,39,111.44444596,37
,30.0342862,0\H,66,1.09886407,39,111.4259897,37,151.1652169,0\C,47,1.5
0522253,46,120.97694816,44,-178.6881969,0\H,70,1.09888578,47,111.43827
451,46,-30.16237636,0\H,70,1.10215834,47,110.95950052,46,89.28728585,0
\H,70,1.09889558,47,111.42158754,46,-151.28127237,0\C,43,1.50602633,42
,120.75203348,19,1.45469051,0\H,74,1.10061171,43,111.70608081,42,-67.0
8100558,0\H,74,1.10034638,43,111.2015735,42,51.66296836,0\H,74,1.09782
843,43,110.85151491,42,172.35880356,0\C,44,1.50649314,42,120.97093812,
19,1.23857647,0\H,78,1.10071494,44,111.72969013,42,-71.43721691,0\H,78
,1.09989204,44,111.32409288,42,47.42872481,0\H,78,1.09789328,44,110.76
223776,42,168.23430119,0\C,52,1.50648873,50,120.96879206,21,1.24596452
,0\H,82,1.10069543,52,111.73363342,50,-71.27087955,0\H,82,1.09991351,5
2,111.31897753,50,47.59223083,0\H,82,1.09788922,52,110.76278256,50,168
.38968144,0\C,51,1.50602664,50,120.74978027,21,1.46141534,0\H,86,1.100
59989,51,111.70817224,50,-66.95438454,0\H,86,1.10036065,51,111.1982981
1,50,51.78799945,0\H,86,1.09782628,51,110.85237167,50,172.47838487,0\C
,55,1.50522069,54,120.97718961,52,-178.68761639,0\H,90,1.10215878,55,1
10.95945783,54,89.31392624,0\H,90,1.09889556,55,111.42189738,54,-151.2
5489468,0\H,90,1.09888533,55,111.4380549,54,-30.13594579,0\H,26,1.0921
3617,24,120.50928612,18,179.93271318,0\H,30,1.09213578,28,120.50936382
,22,179.93946167,0\C,23,1.42158961,17,124.39460019,11,0.79205976,0\C,2
5,1.41634932,23,119.05009049,17,-179.96589399,0\C,29,1.41635216,27,119
.05054776,20,-179.97453564,0\C,27,1.42158741,20,124.39395453,2,0.82719
953,0\C,98,1.38036827,29,121.36810877,27,0.05834477,0\C,99,1.37985071,
27,121.42218022,20,179.89999335,0\C,96,1.3798492,23,121.42200879,17,17
9.89848786,0\C,97,1.3803713,25,121.36750965,23,0.04783414,0\H,98,1.092
56499,29,118.31666536,27,-179.95252596,0\H,100,1.09112873,98,120.26556
639,29,179.98249648,0\H,101,1.09133927,99,119.70733973,27,-179.9297817
5,0\H,99,1.08757847,27,119.54471889,20,0.20158789,0\H,96,1.0875753,23,
119.54967363,17,0.17594097,0\H,102,1.09133457,96,119.70499948,23,-179.
93896753,0\H,103,1.09112812,97,120.26504321,25,179.98659895,0\H,97,1.0
925649,25,118.31699196,23,-179.95681059,0\\Version=ES64L-G16RevC.01\St
ate=2-A\HF=-2390.3172054\S2=0.988654\S2-1=0.\S2A=0.773669\RMSD=4.866e-
09\Dipole=-0.0702768,-0.0033946,-0.1899021\Quadrupole=-1.803827,-15.28
1645,17.085472,-3.6213698,6.3249604,1.0092512\PG=C01 [X(C62H49)]\\@

Archive entry for radical **25**


```
1\1\GINC-N1105\SP\UPBE1PBE\def2TZVP\C62H49(2)\KA_LM9425\08-Oct-2024\0\
\# sp upbe1pbe/def2tzvp geom=connectivity empiricaldispersion=gd3bj\\T
itle Card Required\\0,2\C\C,1,1.4469752\C,2,1.38104493,1,120.91450676\
C,3,1.41461708,2,117.94019468,1,0.44080379,0\C,4,1.44786296,3,121.1793
4488,2,-0.96698557,0\C,5,1.38376819,4,120.78387908,3,0.5889163,0\H,3,1
.09319153,2,122.02923382,1,-179.36726986,0\H,6,1.09309039,5,121.903998
45,4,-179.57598582,0\C,4,2.3038037,3,165.82155508,2,-177.64223545,0\C,
9,1.41462327,4,165.81777777,3,-6.10221565,0\C,10,1.38103905,9,117.9388
6622,4,-177.50551947,0\C,11,1.44696953,10,120.91553118,9,0.44330669,0\
C,12,1.41269569,11,121.20592379,10,0.49290196,0\C,13,1.38377723,12,117
.96557971,11,-0.87168209,0\H,10,1.09319441,9,120.03267764,4,2.29638429
,0\H,13,1.09309096,12,120.13011471,11,179.0327935,0\C,11,1.45557739,10
,132.35545788,9,-179.79870767,0\C,17,1.42724923,11,108.04048521,10,-17
9.96939025,0\C,12,1.41886715,11,108.8865778,10,179.72549687,0\C,2,1.45
557415,1,106.72803824,6,-179.3341964,0\C,1,1.41884836,6,129.90147844,5
,-179.93201472,0\C,20,1.42725168,2,108.04060263,1,-0.18369577,0\C,17,1
.40190675,11,130.76766484,10,0.1104759,0\C,18,1.4312938,17,120.2714696
9,11,-178.91121635,0\C,23,1.38430753,17,119.20681414,11,179.86201029,0
\C,25,1.42325128,23,121.71015899,17,-0.59867864,0\C,20,1.40190949,2,13
0.76919368,1,179.896339,0\C,22,1.43129248,20,120.27215472,2,-178.91756
73,0\C,27,1.3843058,20,119.20829489,2,179.86312897,0\C,29,1.42325038,2
7,121.70977562,20,-0.59549235,0\C,4,1.42244861,3,129.91253021,2,-179.9
1205791,0\C,31,1.47513106,4,125.91623006,3,-0.96896011,0\C,32,1.412215
56,31,119.97112885,4,105.82256929,0\C,32,1.41240082,31,119.9420306,4,-
74.46436837,0\C,33,1.39864124,32,118.94697661,31,179.97304999,0\C,34,1
.39847561,32,118.95087341,31,-179.65346619,0\C,35,1.39844249,33,121.98
977679,32,-0.22679996,0\H,35,1.09369444,33,118.72743241,32,179.5711963
1,0\H,36,1.09370146,34,118.72549602,32,179.08367338,0\C,19,1.47638034,
12,123.58192675,11,-178.997315,0\C,40,1.41103795,19,119.95582777,12,-7
9.55036888,0\C,40,1.41188096,19,119.89908918,12,99.57005616,0\C,41,1.3
9897017,40,118.93794989,19,179.45541453,0\C,42,1.39781753,40,118.98139
363,19,-179.21129056,0\C,43,1.39844357,41,121.94799861,40,-0.49324021,
0\H,43,1.09367183,41,118.75314095,40,179.14835236,0\H,44,1.09372798,42
,118.75659002,40,179.99685927,0\C,21,1.47638974,1,123.58308785,6,0.153
42261,0\C,48,1.41099117,21,119.95565252,1,-79.57519599,0\C,48,1.411914
74,21,119.89866469,1,99.54545955,0\C,49,1.39901114,48,118.93725068,21,
179.45553392,0\C,50,1.39777952,48,118.98181976,21,-179.21184811,0\C,51
,1.39840259,49,121.9483649,48,-0.49232811,0\H,51,1.09366764,49,118.753
91014,48,179.1505002,0\H,52,1.09373263,50,118.75622434,48,179.99729536
,0\C,34,1.50630197,32,120.83368409,31,-0.98103761,0\H,56,1.10018229,34
,111.29994585,32,-50.23407843,0\H,56,1.10066572,34,111.64605744,32,68.
5428007,0\H,56,1.09781796,34,110.82828812,32,-171.01270963,0\C,33,1.50
631461,32,120.85507564,31,-0.96846728,0\H,60,1.1001497,33,111.27625455
,32,-50.04105972,0\H,60,1.10067995,33,111.67464092,32,68.74028805,0\H,
60,1.09782157,33,110.82763409,32,-170.79720704,0\C,37,1.50533896,35,12
0.98935851,33,178.68235393,0\H,64,1.10212792,37,110.95888611,35,-90.46
288224,0\H,64,1.09882016,37,111.44331838,35,29.00872065,0\H,64,1.09893
49,37,111.42609145,35,150.12734265,0\C,45,1.50537388,43,120.99433499,4
1,-178.36305443,0\H,68,1.10214637,45,110.94068875,43,91.5635763,0\H,68
,1.09900748,45,111.44726131,43,-149.04551261,0\H,68,1.09877767,45,111.
42901626,43,-27.92269457,0\C,41,1.50566742,40,120.5635622,19,-1.929805
62,0\H,72,1.10061869,41,111.17918017,40,-53.62522688,0\H,72,1.10064229
,41,111.48534865,40,65.04711089,0\H,72,1.09779587,41,110.94223452,40,-
174.32336558,0\C,42,1.50464726,40,120.37654104,19,1.47506152,0\H,76,1.
10021238,42,111.32407137,40,-53.22001181,0\H,76,1.10153684,42,111.0845
6156,40,65.46112631,0\H,76,1.09778076,42,111.03363957,40,-174.23498224
,0\C,50,1.5046472,48,120.37385118,21,1.47675819,0\H,80,1.10021218,50,1
11.3242716,48,-53.23418901,0\H,80,1.1015386,50,111.08353613,48,65.4459
5738,0\H,80,1.09778081,50,111.03421387,48,-174.24977343,0\C,49,1.50566
567,48,120.56415201,21,-1.92396596,0\H,84,1.10061514,49,111.18008095,4
8,-53.60685946,0\H,84,1.10064715,49,111.48352634,48,65.0654139,0\H,84,
1.09779569,49,110.94293463,48,-174.3067695,0\C,53,1.50537538,51,121.00
```

202347,49,-178.36898232,0\H,88,1.0990379,53,111.44773217,51,-148.47893
546,0\H,88,1.09875096,53,111.42759255,51,-27.3585513,0\H,88,1.1021417,
53,110.94160209,51,92.14174799,0\H,29,1.09226923,27,120.14108193,20,17
9.74428336,0\H,27,1.09196451,20,120.46012432,2,-0.27110415,0\H,23,1.09
196231,17,120.46103551,11,-0.27460936,0\H,25,1.09226853,23,120.1403004
8,17,179.74164625,0\C,30,1.41765774,29,120.83064273,27,-179.42761651,0
\C,28,1.41686948,22,124.19790449,20,178.36751266,0\C,24,1.41686558,18,
124.19824926,17,178.35894751,0\C,26,1.41765764,25,120.83027409,23,-179
.42397165,0\C,96,1.38053797,30,121.18112162,29,179.39752844,0\C,97,1.3
8329483,28,121.02352851,22,179.757312,0\C,98,1.38329593,24,121.0236018
6,18,179.75676735,0\C,99,1.3805378,26,121.18110857,25,179.39466273,0\H
,96,1.09260618,30,118.41777646,29,-0.44015735,0\H,100,1.09115434,96,12
0.24722577,30,-179.95331043,0\H,101,1.09146311,97,119.57553426,28,179.
95519219,0\H,97,1.08948251,28,119.21495776,22,-0.57058157,0\H,98,1.089
48396,24,119.2150797,18,-0.57143743,0\H,102,1.09146277,98,119.57556508
,24,179.95478546,0\H,103,1.09115508,99,120.2472059,26,-179.95332868,0\
H,99,1.09260581,26,118.41758069,25,-0.44237943,0\\Version=ES64L-G16Rev
C.01\State=2-A\HF=-2390.3233978\S2=1.020104\S2-1=0.\S2A=0.783049\RMSD=
4.897e-09\Dipole=-0.0420193,-0.0037711,-0.0980367\Quadrupole=-3.210491
5,-13.8639979,17.0744895,0.4612539,6.5817654,0.0562441\PG=C01 [X(C62H4
9)]\\@

10. Frequency Analyses and Visualized IR Spectra

Frequency analyses of radicals **24** and **25** were carried out at the uM06/6-311++g(d,p) level; no imaginary frequencies were detected; lowest frequencies at $\tilde{\upsilon}$ = 10.9, 9.0, 16.6, 21.3, 24.8. 26.0 cm^{-1}, etc. for radical **24** and $\tilde{\upsilon}$ = 10.5, 13.9, 14.5, 21.8, 24.5. 27.8 cm^{-1}, etc. for radical **25**. These are attributable to the torsion of the heptacycle along the molecule axis and various twisting and bending modes of the core and the mesityl groups, and to rotations of the methyl groups.

Extract from the frequency calculation output for radical **24**:

The imaginary frequencies can be attributed to vibrations caused by bending and twisting of the scaffold and are mainly induced by the mesityl moieties. The occurance of these imaginary frequencies could not be prevented by further optimization of the molecule; it should have virtually no influence of further calculations with the thus obtained structure.

```
Low frequencies ---  -12.4638  -10.9610   -8.9554   -5.8990   -0.0007   -0.0005
Low frequencies ---    0.0006    3.5803   16.5901
[…]
Harmonic frequencies (cm**-1), IR intensities (KM/Mole), reduced masses (AMU), force
constants (mDyne/A):
                      1                      2                      3
                      A                      A                      A
 Frequencies --    -10.9450               -9.0210                16.5532
 Red. masses --      4.8352                4.6231                 5.3312
 Frc consts  --      0.0003                0.0002                 0.0009
 IR Inten    --      0.0000                0.1059                 0.0553
 [...]
                      4                      5                      6
                      A                      A                      A
 Frequencies --     21.2954               24.8015                25.9896
 Red. masses --      4.7984                3.6122                 3.9953
 Frc consts  --      0.0013                0.0013                 0.0016
 IR Inten    --      0.0038                0.0000                 0.0564
 [...]
                      7                      8                      9
                      A                      A                      A
 Frequencies --     27.1055               27.1522                30.1901
 Red. masses --      4.1962                3.8115                 4.5646
 Frc consts  --      0.0018                0.0017                 0.0025
 IR Inten    --      0.0420                0.0169                 0.0773
[...]
                     10                     11                     12
                      A                      A                      A
 Frequencies --     33.3794               37.9052                45.7566
 Red. masses --      5.0920                4.6075                 1.0258
 Frc consts  --      0.0033                0.0039                 0.0013
 IR Inten    --      0.0001                0.0048                 0.1692
 [...]
                     13                     14                     15
                      A                      A                      A
 Frequencies --     46.8012               47.3040                49.9070
 Red. masses --      1.0224                1.0205                 5.2994
 Frc consts  --      0.0013                0.0013                 0.0078
 IR Inten    --      0.0009                0.3239                 0.0807
```

Extract from the frequency calculation output for radical **25**:

```
Low frequencies ---  -10.8138   -0.0005    0.0009    0.0009    2.2765    6.0891
Low frequencies ---   13.5882   14.3198   15.0443
[…]
Harmonic frequencies (cm**-1), IR intensities (KM/Mole), reduced masses (AMU), force
constants (mDyne/A):
                      1                      2                      3
                      A                      A                      A
 Frequencies --     10.5429                13.8528                14.5499
 Red. masses --      4.7588                 5.4508                 4.8101
 Frc consts  --      0.0003                 0.0006                 0.0006
 IR Inten    --      0.1203                 0.0037                 0.0002
[...]
                      4                      5                      6
                      A                      A                      A
 Frequencies --     21.7729                24.4931                27.7739
 Red. masses --      4.7448                 3.6451                 4.4455
 Frc consts  --      0.0013                 0.0013                 0.0020
 IR Inten    --      0.0002                 0.0004                 0.0656
[...]
                      7                      8                      9
                      A                      A                      A
 Frequencies --     33.2629                33.4418                36.5239
 Red. masses --      4.1479                 4.0869                 1.0481
 Frc consts  --      0.0027                 0.0027                 0.0008
 IR Inten    --      0.1121                 0.0020                 0.0412
[...]
                     10                     11                     12
                      A                      A                      A
 Frequencies --     38.2953                38.9200                40.3080
 Red. masses --      1.4729                 1.8005                 1.0384
 Frc consts  --      0.0013                 0.0016                 0.0010
 IR Inten    --      0.0288                 0.3141                 0.1825
[...]
                     13                     14                     15
                      A                      A                      A
 Frequencies --     46.0929                50.8119                51.5047
 Red. masses --      4.8413                 5.2179                 4.0618
 Frc consts  --      0.0061                 0.0079                 0.0063
 IR Inten    --      0.0017                 0.0976                 0.0493
```

Calculated IR Spectrum of radical **24** (top spectrum) visualized with GaussSum: Scaling factor of 0.9619; FWHM: 10 cm^{-1}. Measured and aligned IR spectra (bottom spectrum) for comparison.

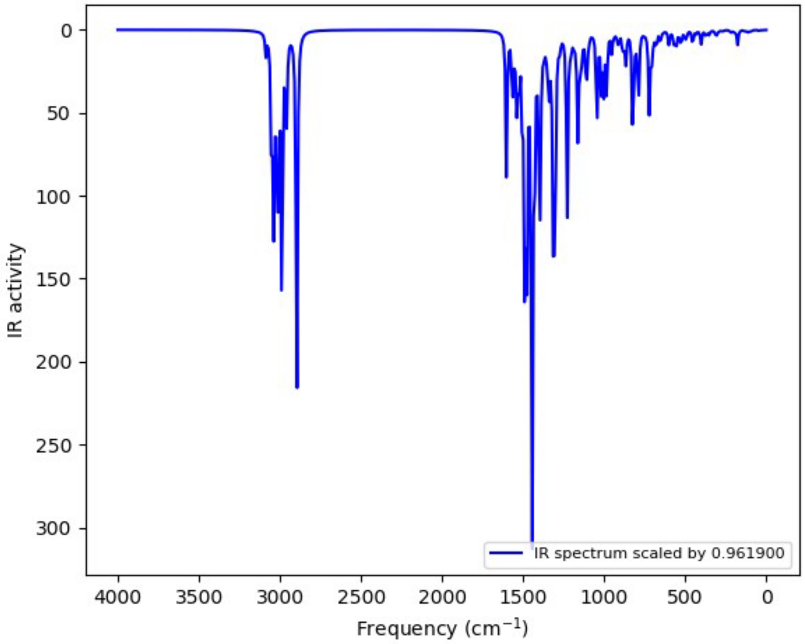

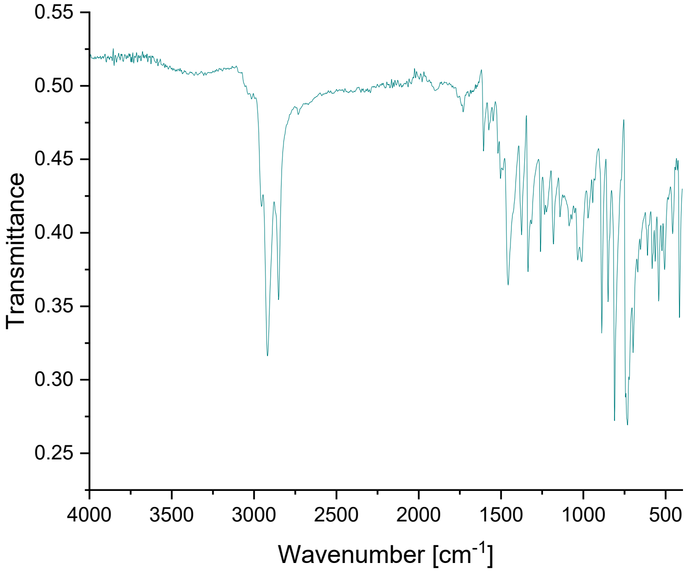

Calculated IR Spectrum of radical **25** (top spectrum) visualized with GaussSum: Scaling factor of 0.9619; FWHM: 10 cm^{-1}. Measured and aligned IR spectra (bottom spectrum) for comparison.

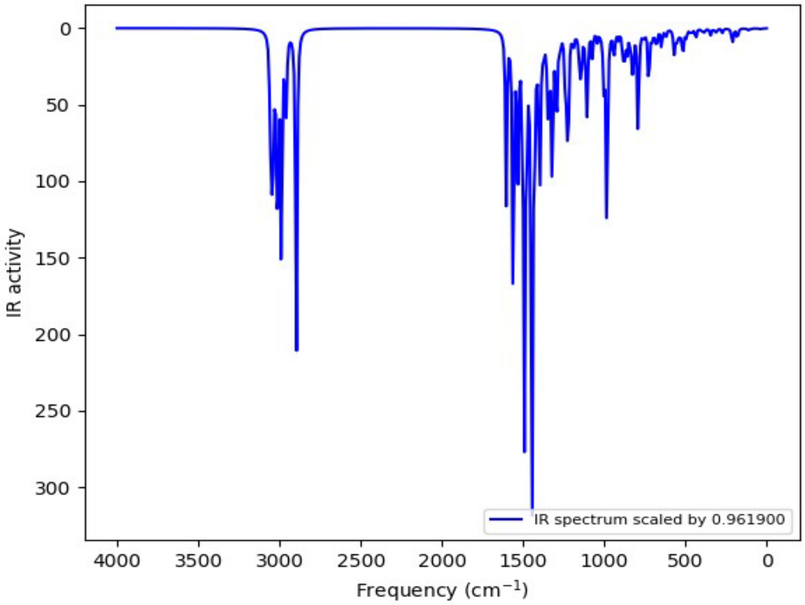

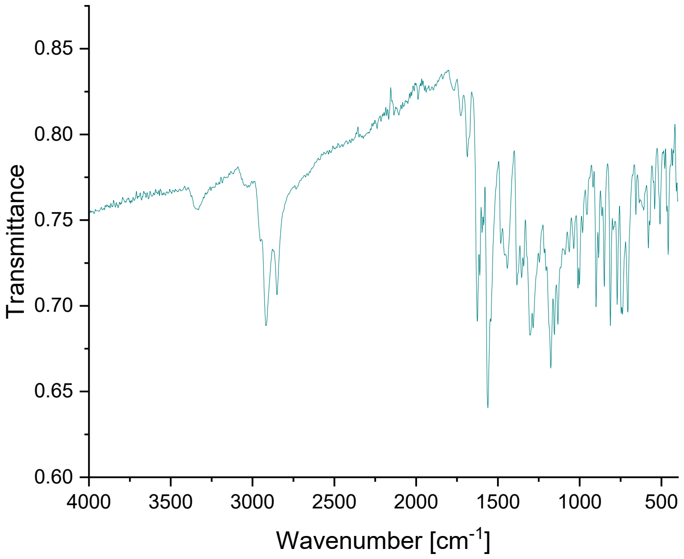

11. SOMOs of α and β Electrons (magnified) of Radical 24 (top) and Radical 25 (bottom)

upbe0/def2tzvp (GD3BJ) (Isovalue: 0.02 electrons$^{1/2}$ bohr$^{-3/2}$)

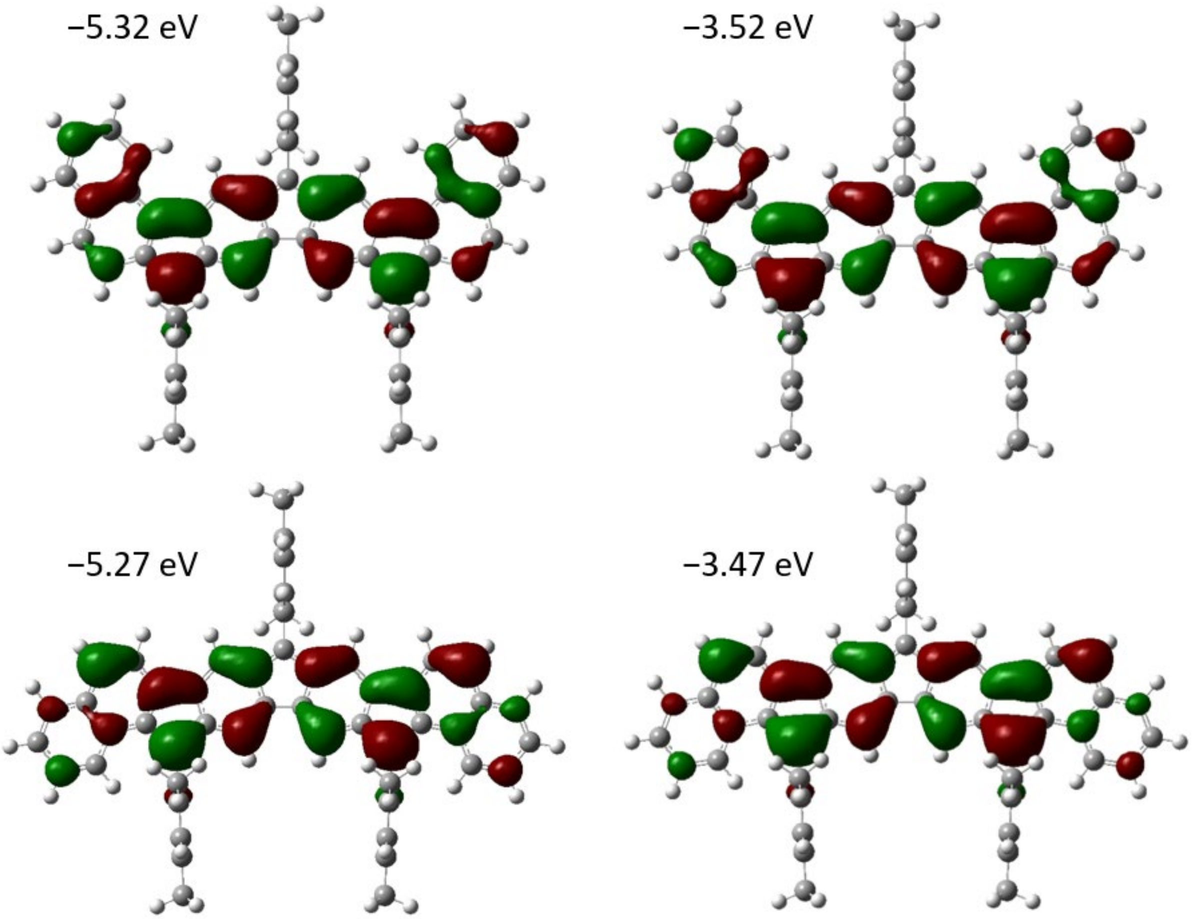

12. Calculated Spin Densities (magnified) of Radical 24 (top) and 25 (bottom)

blue: α spin, green: β spin (isovalue: 0.004 electrons·bohr^{-3}), calculated at the upbe0/def2-TZVP/GD3BJ level

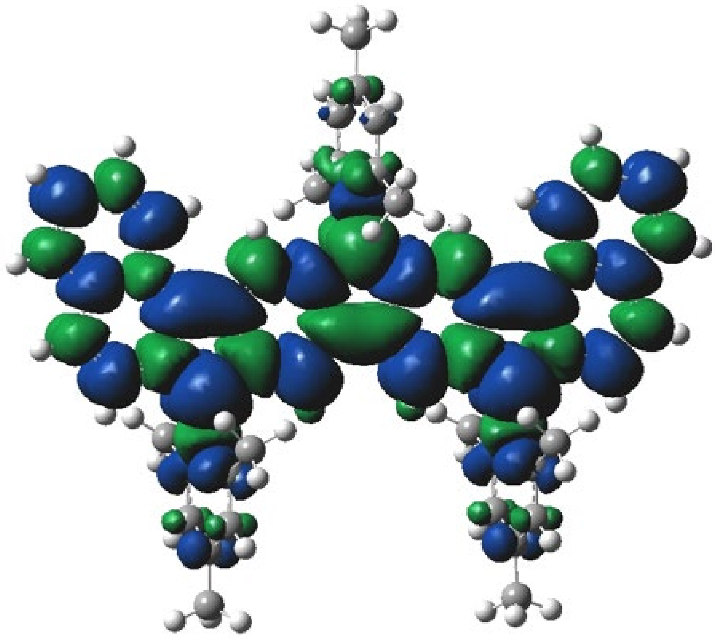

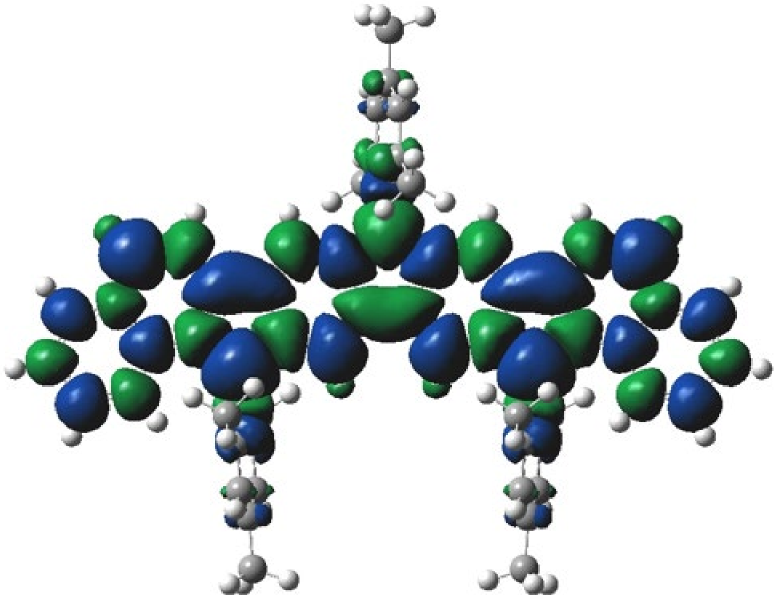

13. TD Calculations

upbe0/def2tzvp (GD3BJ, CH_2Cl_2)

Excerpt of output file of radical **24** and **25**, where only a selected number of transitions are given. Text after // was added as comment by the authors.

Radical **24**:

```
**********************************************************************

 Excited states from <AA,BB:AA,BB> singles matrix:

**********************************************************************

       state          X           Y           Z        Dip. S.      Osc.
         1        -0.0004      0.0800     -0.0002      0.0064      0.0002
         2         0.8010     -0.0002      0.0167      0.6418      0.0189
         3         0.8476     -0.0001      0.0174      0.7188      0.0265
         4         2.9426     -0.0005      0.0554      8.6621      0.3576
         5         0.0013      0.0545      0.0001      0.0030      0.0001
         6        -3.6067      0.0004     -0.0696     13.0129      0.5828
         7         0.1494     -0.0001      0.0042      0.0223      0.0012
         8         0.0000      0.3422      0.0001      0.1171      0.0065
         9         0.0003      0.4301      0.0010      0.1850      0.0110
        10        -0.0030      0.8877     -0.0003      0.7881      0.0483
[…]
Excitation energies and oscillator strengths:

Excited State   1:  2.305-A      1.0324 eV 1200.91 nm  f=0.0002  <S**2>=1.078
208B -> 212B      -0.17583 // HOMO-2 -> LUMO+1
209B -> 212B      -0.09679 // HOMO-1 -> LUMO+1

Excited State   2:  2.135-A      1.2014 eV 1031.97 nm  f=0.0189  <S**2>=0.890
211A -> 212A      -0.52011 // SOMO -> LUMO
209B -> 211B       0.78973 // HOMO-1 -> LUMO
210B -> 212B      -0.28300 // SOMO -> LUMO+1

Excited State   3:  3.095-A      1.5020 eV  825.45 nm  f=0.0265  <S**2>=2.146
210A -> 212A       0.65382 // HOMO-1 -> LUMO
211A -> 212A       0.35718 // SOMO -> LUMO
208B -> 211B       0.37693 // HOMO-2 -> LUMO
210B -> 212B      -0.50969 // SOMO -> LUMO+1

Excited State   4:  2.269-A      1.6848 eV  735.88 nm  f=0.3576  <S**2>=1.038
210A -> 212A      -0.57614 // HOMO-1 -> LUMO
211A -> 212A       0.64654 // SOMO -> LUMO
208B -> 211B       0.18072 // HOMO-2 -> LUMO
209B -> 211B       0.37853 // HOMO-1 -> LUMO
210B -> 212B      -0.18401 // SOMO -> LUMO+1

Excited State   5:  3.217-A      1.7240 eV  719.15 nm  f=0.0001  <S**2>=2.337
199A -> 212A       0.10414 // HOMO-12 -> LUMO
208A -> 212A       0.18078 // HOMO-3 -> LUMO
209A -> 212A      -0.61637 // HOMO-2 -> LUMO
209B -> 212B       0.71469 // HOMO-1 -> LUMO+1

Excited State   6:  2.185-A      1.8282 eV  678.18 nm  f=0.5828  <S**2>=0.943
201A -> 212A       0.09053 // HOMO-10 -> LUMO
210A -> 212A      -0.43571 // HOMO-1 -> LUMO
210A -> 213A       0.17124 // HOMO-1 -> LUMO+1
```

```
211A -> 212A      -0.39077 // SOMO -> LUMO
208B -> 211B       0.46174 // HOMO-2 -> LUMO
209B -> 211B      -0.43912 // HOMO-1 -> LUMO
210B -> 212B      -0.42056 // SOMO -> LUMO+1

Excited State   7:  2.479-A      2.1580 eV  574.54 nm  f=0.0012  <S**2>=1.287
210A -> 212A       0.10948 // HOMO-1 -> LUMO
211A -> 213A      -0.17716 // SOMO -> LUMO+1
201B -> 212B      -0.18462 // HOMO-9 -> LUMO+1
208B -> 211B       0.68740 // HOMO-2 -> LUMO
209B -> 211B       0.13075 // HOMO-1 -> LUMO
210B -> 212B       0.63111 // SUMO -> LUMO+1

Excited State   8:  3.241-A      2.2594 eV  548.75 nm  f=0.0065  <S**2>=2.377
208A -> 212A       0.52833 // HOMO-3 -> LUMO
208A -> 213A       0.15407 // HOMO-3 -> LUMO+1
209A -> 212A       0.24098 // HOMO-2 -> LUMO
209A -> 213A       0.11757 // HOMO-2 -> LUMO+1
210A -> 214A      -0.27179 // HOMO-1 -> LUMO+2
201B -> 211B       0.34686 // HOMO-9 -> LUMO
207B -> 211B       0.11019 // HOMO-3 -> LUMO
208B -> 212B      -0.46157 // HOMO-2 -> LUMO+1
208B -> 213B      -0.13345 // HOMO-2 -> LUMO+2
210B -> 211B      -0.12820 // SOMO -> LUMO
210B -> 214B       0.20332 // SOMO -> LUMO+3

Excited State   9:  2.198-A      2.4166 eV  513.06 nm  f=0.0110  <S**2>=0.958
209A -> 212A       0.70464 // HOMO-2 -> LUMO
200B -> 211B       0.10652 // HOMO-10 -> LUMO
201B -> 211B      -0.20633 // HOMO-9 -> LUMO
208B -> 212B       0.17487 // HOMO-2 -> LUMO+1
209B -> 212B       0.59242 // HOMO-1 -> LUMO+1
209B -> 213B       0.11941 // HOMO-1 -> LUMO+2
210B -> 214B      -0.11833 // SOMO -> LUMO+3

Excited State  10:  2.336-A      2.5018 eV  495.58 nm  f=0.0483  <S**2>=1.114
206B -> 212B      -0.27747 // HOMO-4 -> LUMO+1
207B -> 211B       0.93536 // HOMO-3 -> LUMO
```

Radical **25**:

```
**********************************************************************

Excited states from <AA,BB:AA,BB> singles matrix:

**********************************************************************


       state          X           Y           Z        Dip. S.      Osc.
         1        -0.0005     -0.0011      0.0001      0.0000      0.0000
         2         1.1739     -0.0003      0.0084      1.3782      0.0402
         3        -1.2929      0.0002      0.0016      1.6717      0.0641
         4        -3.7058      0.0007     -0.0020     13.7327      0.5531
         5        -0.0070     -0.0763      0.0001      0.0059      0.0002
         6        -3.3047      0.0004     -0.0047     10.9211      0.5090
         7        -0.1611      0.0006     -0.0015      0.0259      0.0015
         8        -0.0003     -0.8026      0.0006      0.6441      0.0371
         9         0.0005      0.1319     -0.0005      0.0174      0.0010
        10         0.0010     -0.4259      0.0001      0.1814      0.0112
[…]
Excitation energies and oscillator strengths:
Excited State   1:  2.312-A      1.1205 eV 1106.52 nm  f=0.0000  <S**2>=1.087
208B -> 212B      -0.16153 // HOMO-2 -> LUMO+1
209B -> 212B       0.10514 // HOMO-1 -> LUMO+1
210B -> 211B       0.97107 // SOMO -> LUMO

Excited State   2:  2.093-A      1.1894 eV 1042.44 nm  f=0.0402  <S**2>=0.845
211A -> 212A       0.51699 // SOMO -> LUMO
209B -> 211B       0.81177 // HOMO-1 -> LUMO
210B -> 212B       0.23613 // SOMO -> LUMO+1

Excited State   3:  3.170-A      1.5648 eV  792.33 nm  f=0.0641  <S**2>=2.262
210A -> 212A       0.78367 // HOMO-1 -> LUMO
211A -> 212A       0.24567 // SOMO -> LUMO
208B -> 211B       0.26376 // HOMO-2 -> LUMO
210B -> 212B      -0.47504 // SOMO -> LUMO+1

Excited State   4:  2.200-A      1.6439 eV  754.19 nm  f=0.5531  <S**2>=0.960
210A -> 212A      -0.41196 // HOMO-1 -> LUMO
211A -> 212A       0.75575 // SOMO -> LUMO
208B -> 211B       0.14053 // HOMO-2 -> LUMO
209B -> 211B      -0.40230 // HOMO-1 -> LUMO
210B -> 212B      -0.19469 // SOMO -> LUMO+1

Excited State   5:  3.195-A      1.6636 eV  745.28 nm  f=0.0002  <S**2>=2.303
200A -> 212A       0.10988 // HOMO-11 -> LUMO
208A -> 212A      -0.15019 // HOMO-3 -> LUMO
209A -> 212A      -0.62322 // HOMO-2 -> LUMO
208B -> 212B       0.10149 // HOMO-2 -> LUMO+1
209B -> 212B       0.71673 // HOMO-1 -> LUMO+1

Excited State   6:  2.243-A      1.9024 eV  651.71 nm  f=0.5090  <S**2>=1.008
210A -> 212A       0.42444 // HOMO-1 -> LUMO
210A -> 213A      -0.15162 // HOMO-1 -> LUMO+1
211A -> 212A       0.25700 // SOMO -> LUMO
208B -> 211B      -0.37813 // HOMO-2 -> LUMO
209B -> 211B      -0.38915 // HOMO-1 -> LUMO
210B -> 212B       0.62039 // SOMO -> LUMO+1

Excited State   7:  2.520-A      2.2950 eV  540.23 nm  f=0.0015  <S**2>=1.338
```

```
209A -> 214A      -0.11033 // HOMO-2 -> LUMO+2
211A -> 213A      -0.23097 // SOMO -> LUMO+1
211A -> 215A       0.10901 // SOMO -> LUMO+3
199B -> 212B       0.12943 // HOMO-11 -> LUMO+1
205B -> 212B       0.12805 // HOMO-5 -> LUMO+1
207B -> 212B       0.13233 // HOMO-3 -> LUMO+1
208B -> 211B       0.74254 // HOMO-2 -> LUMO
210B -> 212B       0.48833 // SOMO -> LUMO+1

Excited State   8:  2.430-A      2.3509 eV  527.39 nm  f=0.0371  <S**2>=1.226
205A -> 212A       0.11185 // HOMO-6 -> LUMO
208A -> 212A      -0.29111 // HOMO-3 -> LUMO
209A -> 212A       0.68249 // HOMO-2 -> LUMO
205B -> 211B       0.10493 // HOMO-5 -> LUMO
207B -> 211B       0.15688 // HOMO-3 -> LUMO
208B -> 212B       0.17030 // HOMO-2 -> LUMO+1
209B -> 212B       0.53832 // HOMO-1 -> LUMO+1

Excited State   9:  2.696-A      2.3599 eV  525.38 nm  f=0.0010  <S**2>=1.567
208A -> 212A      -0.27133 // HOMO-3 -> LUMO
209A -> 212A      -0.29989 // HOMO-2 -> LUMO
209A -> 215A      -0.17270 // HOMO-2 -> LUMO+3
210A -> 214A      -0.10156 // HOMO-1 -> LUMO+2
211A -> 214A       0.20837 // SOMO -> LUMO+2
199B -> 211B       0.10176 // HOMO-11 -> LUMO
200B -> 212B      -0.15151 // HOMO-10 -> LUMO+1
205B -> 211B       0.48223 // HOMO-5 -> LUMO
207B -> 211B       0.41559 // HOMO-3 -> LUMO
208B -> 212B       0.34630 // HOMO-2 -> LUMO+1
209B -> 212B      -0.26634 // HOMO-1 -> LUMO+1
210B -> 214B      -0.10961 // SOMO -> LUMO+3

Excited State  10:  2.351-A      2.5244 eV  491.15 nm  f=0.0112  <S**2>=1.132
204B -> 212B       0.10864 // HOMO-6 -> LUMO+1
205B -> 211B      -0.53527 // HOMO-5 -> LUMO
206B -> 212B      -0.26206 // HOMO-4 -> LUMO+1
207B -> 211B       0.77251 // HOMO-3 -> LUMO
```

Table S1. Photophysical data of radicals **24** (top) and **25** (bottom) calculated at the upbe0/def2tzvp/cpcm (CH_2Cl_2) (GD3BJ) level.

Calculated transition		Oscillator	Transition α spin	Transition β spin
Energy [eV]	Wavelength [nm]	strength, *f*		
1.03	1201	0.0002	–	SOMO → LUMO (94%)<br>H−2 → L+1 (3%)
1.50	825	0.027	H−1 → LUMO (33%)	SOMO → L+1 (26%)
1.68	736	0.36	H−1 → LUMO (33%)<br>SOMO → LUMO (42%)	H−1 → LUMO (14%)<br>SOMO → L+1 (3%)
1.83	678	0.58	H−1 → LUMO (19%)<br>SOMO → LUMO (15%)<br>H−1 → L+1 (3%)	H−2 → LUMO (21%)<br>H−1 → LUMO (19%)<br>SOMO → L+1 (18%)
3.08	403	0.12	H−2 → L+1 (23%)<br>SOMO → L+2 (27%)	H−1 → L+2 (17%)<br>H−2 → L+1 (9%)
3.21	387	0.88	H−1 → L+1 (18%)	SOMO → L+2 (30%)
3.26	380	0.95	H−1 → L+1 (19%)	SOMO → L+2 (21%)

Calculated transition		Oscillator	Transition α spin	Transition β spin
Energy [eV]	Wavelength [nm]	strength, *f*		
1.12	1107	0.0000	–	SOMO → LUMO (94%)<br>H–2 → L+1 (3%)
1.56	792	0.06	H–1 → LUMO (61%)	SOMO → L+1 (23%)
1.64	754	0.55	H–1 → LUMO (17%)<br>SOMO → LUMO (57%)	H–1 → LUMO (16%)
1.90	652	0.51	H–1 → LUMO (18%)	H–2 → LUMO (14%)<br>H–1 → LUMO (15%)<br>SOMO → L+1 (38%)
3.27	378	0.14	H–1 → L+1 (3%)<br>SOMO → L+1 (3%)<br>SOMO → L+3 (5%)	SOMO → L+2 (6%)
3.38	366	1.34	H–1 → L+1 (22%)	H–3 → L+1 (10%)<br>SOMO → L+2 (26%)
3.40	365	1.00	H–1 → L+1 (16%)	SOMO → L+2 (18%)

14. Calculated UV/Vis/NIR Spectra of Radical 24 (top) and 25 (bottom)

upbedef2tzvp (GD3BJ, CH_2Cl_2), visualized with GaussSum: FWHM = 800 cm^{-1}

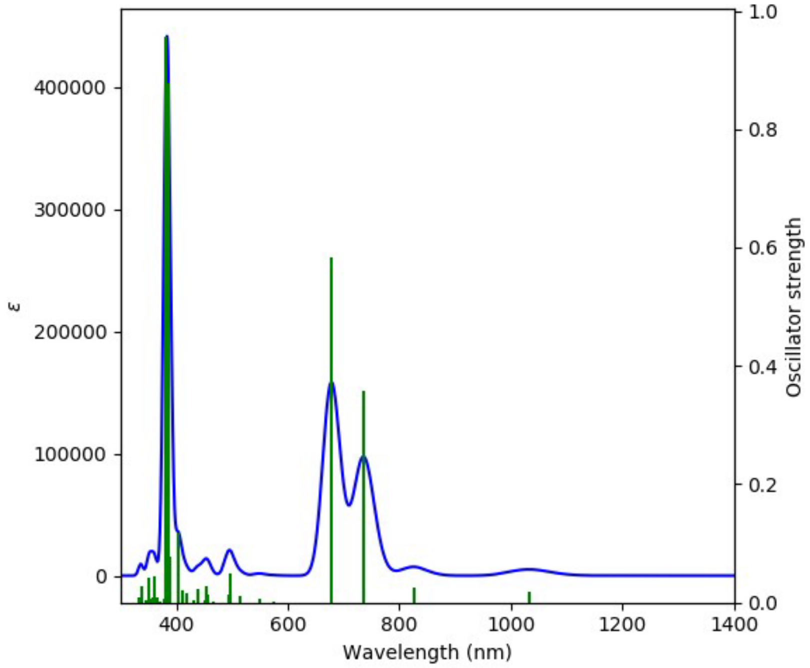

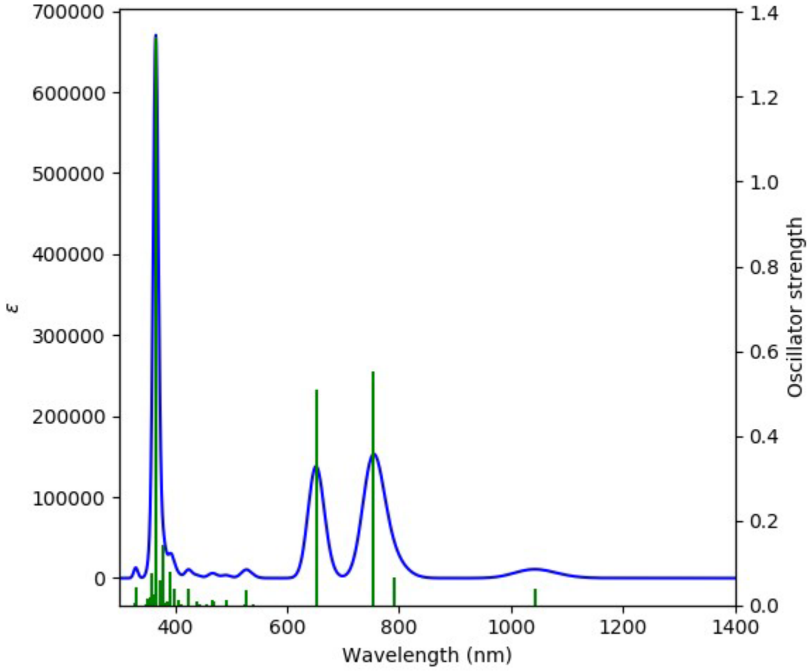

UV/Vis spectra of radicals **24** and **25** with identical section and scaling allowing for comparison: measured (top) and calculated (bottom) spectra:

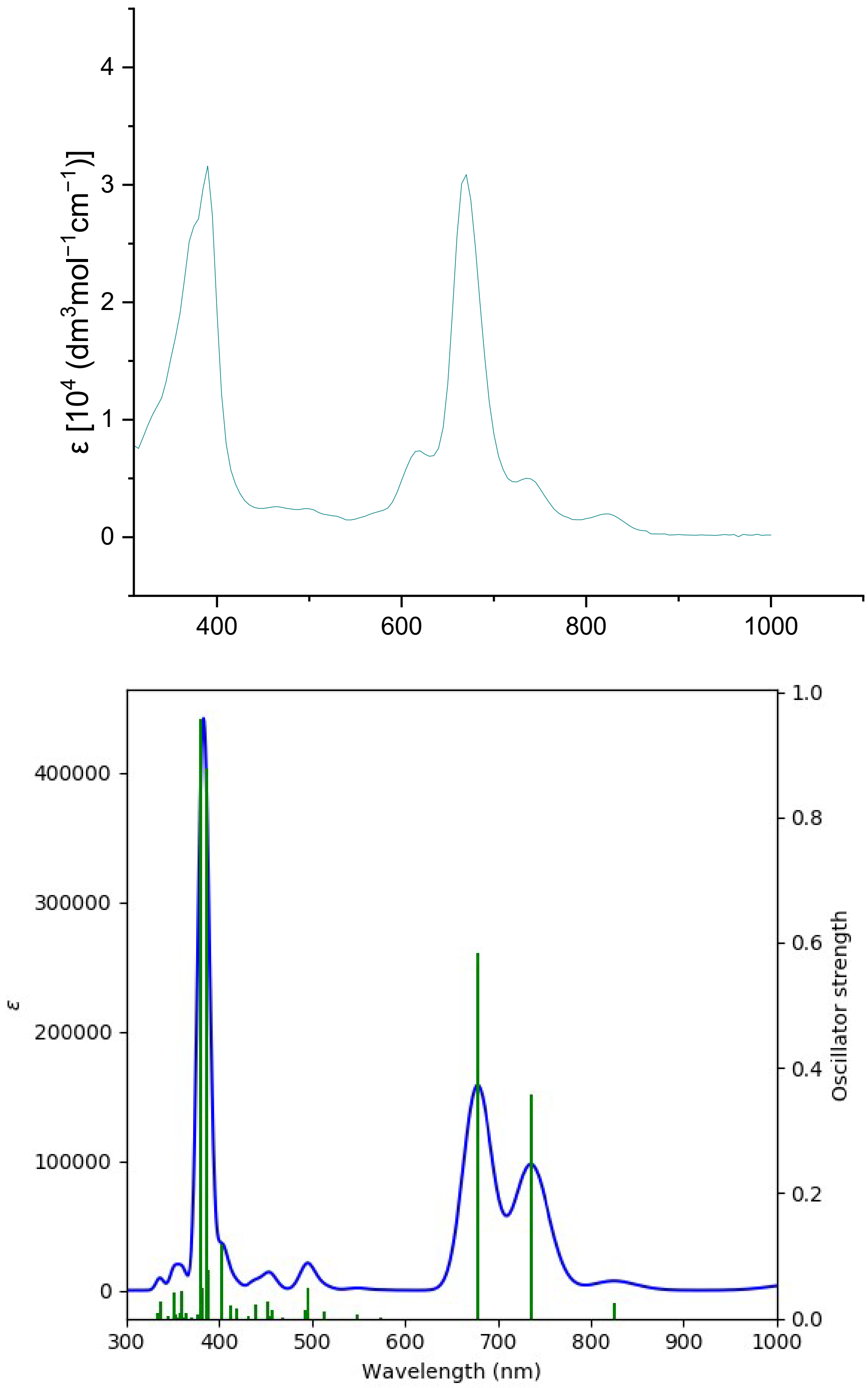

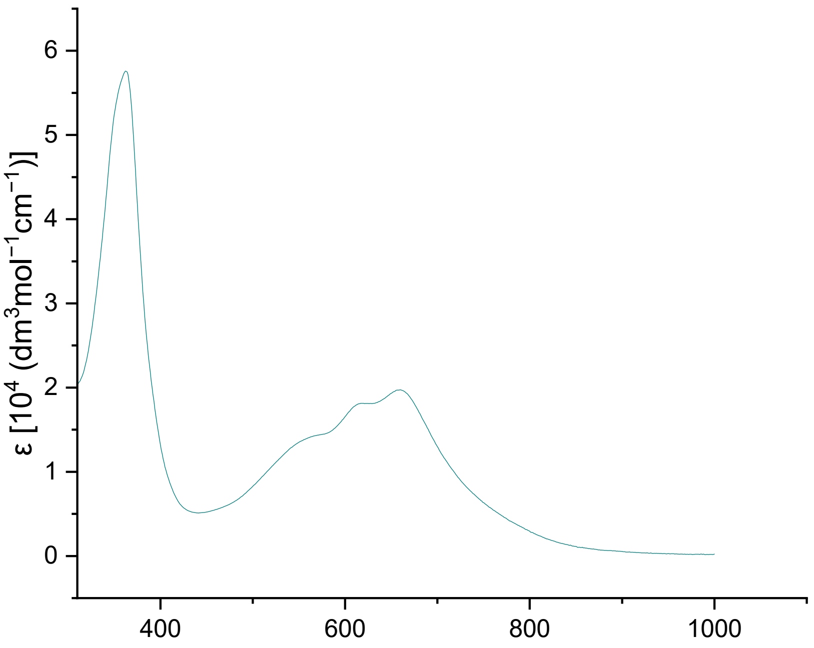

ε [10^4 (dm^3mol^-1cm^-1)]
0
1
2
3
4
5
6
400
600
800
1000

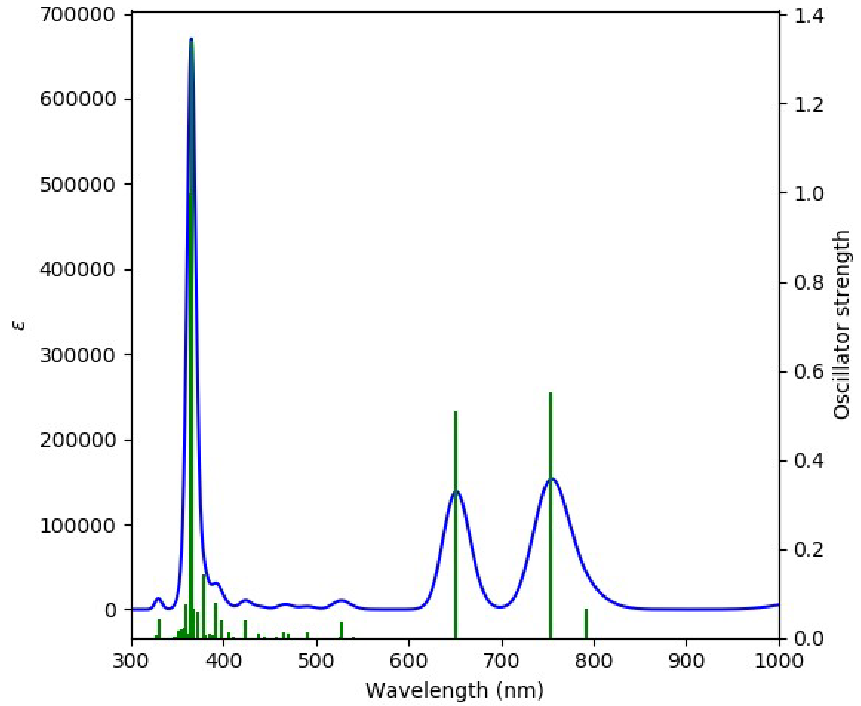

700000
600000
500000
400000
300000
200000
100000
0
ε
1.4
1.2
1.0
0.8
0.6
0.4
0.2
0.0
Oscillator strength
300
400
500
600
700
800
900
1000
Wavelength (nm)

15. Calculated EPR Data for Radicals 24 (top) and 25 (bottom)

upbe0/def2tzvp (GD3BJ)

g value:
$g_{xx} = 2.0023193 + 100.3 \cdot 10^{-6}$
$g_{yy} = 2.0023193 + 290.0 \cdot 10^{-6}$
$g_{zz} = 2.0023193 + 682.5 \cdot 10^{-6}$
$g_{iso} = (g_{xx} + g_{yy} + g_{zz}) / 3 = 2.0026$

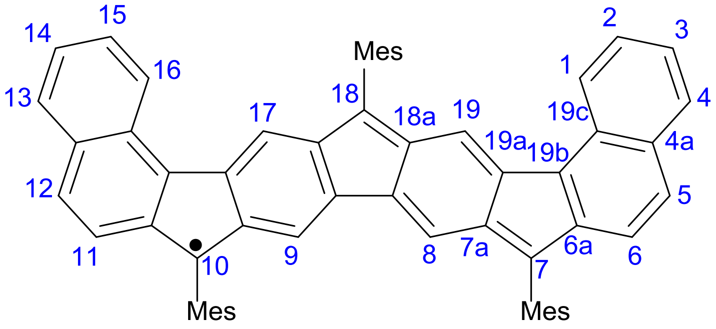

Output (excerpt; boldfacing and IUPAC numbering was given by the authors):

Isotropic Fermi Contact Couplings

	Atom	a.u.	MegaHertz	Gauss	10(-4) cm-1	IUPAC numbering
1	C(13)	-0.02312	-25.99340	-9.27509	-8.67047	
2	C(13)	0.00687	7.71951	2.75451	2.57495	
3	C(13)	-0.01059	-11.90385	-4.24759	-3.97070	C-17
4	C(13)	0.01821	20.47704	7.30672	6.83041	
5	C(13)	-0.00962	-10.81862	-3.86035	-3.60870	
6	C(13)	0.01258	14.13856	5.04499	4.71612	C-9
7	H(1)	0.00116	5.17761	1.84750	1.72706	
8	H(1)	-0.00204	-9.11038	-3.25081	-3.03889	
9	C(13)	0.01821	20.47713	7.30675	6.83043	C-18a
10	C(13)	-0.01059	-11.90488	-4.24796	-3.97104	C-19
11	C(13)	0.00686	7.71735	2.75374	2.57423	C-19a
12	C(13)	-0.02312	-25.99368	-9.27519	-8.67056	C-17a
13	C(13)	0.01257	14.13605	5.04409	4.71528	C-8
14	C(13)	-0.00962	-10.81581	-3.85935	-3.60777	
15	H(1)	0.00116	5.17969	1.84824	1.72776	
16	H(1)	-0.00204	-9.10891	-3.25028	-3.03840	
17	C(13)	0.00852	9.58284	3.41940	3.19649	C-19b
18	C(13)	-0.01940	-21.80379	-7.78014	-7.27296	C-6a
19	C(13)	0.03076	**34.58400**	12.34043	11.53598	C-7
20	C(13)	0.00852	9.57792	3.41764	3.19485	
21	C(13)	0.03076	**34.57842**	12.33844	11.53412	C-10
22	C(13)	-0.01939	-21.79662	-7.77758	-7.27057	
23	C(13)	-0.00900	-10.12194	-3.61176	-3.37632	C-19c
24	C(13)	0.00599	6.72989	2.40139	2.24485	C-6
25	C(13)	0.00673	7.56071	2.69785	2.52198	C-4a
26	C(13)	-0.00641	-7.20482	-2.57086	-2.40327	C-5
27	C(13	-0.00900	-10.11867	-3.61059	-3.37522	
28	C(13)	0.00598	6.72476	2.39956	2.24314	C-11
29	C(13)	0.00672	7.55599	2.69617	2.52041	
30	C(13)	-0.00640	-7.19971	-2.56903	-2.40156	C-12
31	H(1)	-0.00107	-4.77529	-1.70394	-1.59286	

32	H(1)	-0.00107	-4.77787	-1.70486	-1.59373	
33	C(13)	-0.02412	**-27.11569**	-9.67555	-9.04482	C-18
34	C(13)	0.00906	10.18336	3.63368	3.39680	
35	C(13)	-0.00994	-11.17782	-3.98853	-3.72852	
36	C(13)	-0.00993	-11.16156	-3.98272	-3.72309	
37	C(13)	-0.00015	-0.17135	-0.06114	-0.05716	
38	C(13)	-0.00016	-0.18416	-0.06571	-0.06143	
39	C(13)	-0.00059	-0.65928	-0.23525	-0.21991	
40	H(1)	-0.00029	-1.30139	-0.46437	-0.43410	
41	H(1)	-0.00029	-1.29456	-0.46193	-0.43182	
42	C(13)	-0.01428	-16.05530	-5.72893	-5.35547	
43	C(13)	0.01551	17.43143	6.21997	5.81450	
44	C(13)	0.01551	17.43343	6.22068	5.81517	
45	C(13)	0.00011	0.12409	0.04428	0.04139	
46	C(13)	0.00006	0.06301	0.02248	0.02102	
47	C(13)	0.00108	1.21917	0.43503	0.40667	
48	H(1)	0.00048	2.14794	0.76644	0.71647	
49	H(1)	0.00048	2.12550	0.75843	0.70899	
50	C(13)	-0.01428	-16.05254	-5.72794	-5.35455	
51	C(13)	0.01551	17.43278	6.22045	5.81495	
52	C(13)	0.01551	17.43290	6.22049	5.81499	
53	C(13)	0.00011	0.12798	0.04567	0.04269	
54	C(13)	0.00006	0.06867	0.02450	0.02290	
55	C(13)	0.00108	1.21647	0.43407	0.40577	
56	H(1)	0.00048	2.14686	0.76605	0.71611	
57	H(1)	0.00048	2.12428	0.75800	0.70858	
58	C(13)	0.00039	0.43435	0.15499	0.14488	
59	H(1)	-0.00007	-0.31410	-0.11208	-0.10477	
60	H(1)	-0.00015	-0.69048	-0.24638	-0.23032	
61	H(1)	-0.00005	-0.20398	-0.07278	-0.06804	
62	C(13)	0.00040	0.45250	0.16146	0.15094	
63	H(1)	-0.00007	-0.30813	-0.10995	-0.10278	
64	H(1)	-0.00015	-0.68755	-0.24533	-0.22934	
65	H(1)	-0.00004	-0.19668	-0.07018	-0.06560	
66	C(13)	0.00028	0.31349	0.11186	0.10457	
67	H(1)	-0.00022	-0.98470	-0.35136	-0.32846	
68	H(1)	-0.00008	-0.37452	-0.13364	-0.12493	
69	H(1)	-0.00004	-0.17307	-0.06176	-0.05773	
70	C(13)	-0.00051	-0.57355	-0.20466	-0.19132	
71	H(1)	0.00015	0.69226	0.24702	0.23091	
72	H(1)	0.00041	1.84949	0.65994	0.61692	
73	H(1)	0.00007	0.32412	0.11565	0.10811	
74	C(13)	-0.00036	-0.40279	-0.14373	-0.13436	
75	H(1)	0.00046	2.06748	0.73773	0.68964	
76	H(1)	0.00010	0.44397	0.15842	0.14809	
77	H(1)	0.00015	0.66228	0.23632	0.22091	
78	C(13)	-0.00065	-0.73077	-0.26076	-0.24376	
79	H(1)	0.00032	1.42280	0.50769	0.47459	
80	H(1)	0.00008	0.35991	0.12843	0.12005	
81	H(1)	0.00008	0.36923	0.13175	0.12316	
82	C(13)	-0.00065	-0.73366	-0.26179	-0.24472	
83	H(1)	0.00032	1.42529	0.50858	0.47543	
84	H(1)	0.00008	0.36291	0.12950	0.12105	
85	H(1)	0.00008	0.36596	0.13058	0.12207	
86	C(13)	-0.00036	-0.40376	-0.14407	-0.13468	
87	H(1)	0.00046	2.07011	0.73867	0.69051	
88	H(1)	0.00010	0.44530	0.15889	0.14854	
89	H(1)	0.00015	0.65950	0.23533	0.21999	
90	C(13)	-0.00051	-0.57248	-0.20428	-0.19096	
91	H(1)	0.00041	1.84602	0.65870	0.61577	

92	H(1)	0.00007	0.32382	0.11555	0.10802	
93	H(1)	0.00015	0.69043	0.24636	0.23030	
94	H(1)	0.00066	2.93356	1.04677	0.97853	
95	H(1)	0.00066	2.93058	1.04570	0.97754	
96	C(13)	0.00463	5.19977	1.85541	1.73446	C-1
97	C(13)	-0.00586	-6.58865	-2.35099	-2.19774	C-4
98	C(13)	-0.00586	-6.58429	-2.34944	-2.19628	C-13
99	C(13)	0.00462	5.19735	1.85454	1.73365	C-16
100	C(13)	0.00550	6.18165	2.20577	2.06198	C-14
101	C(13)	-0.00492	-5.52558	-1.97166	-1.84314	C-15
102	C(13)	-0.00492	-5.52868	-1.97277	-1.84417	C-2
103	C(13)	0.00550	6.18586	2.20727	2.06338	C-3
104	H(1)	0.00060	2.67508	0.95453	0.89231	
105	H(1)	-0.00104	-4.63031	-1.65221	-1.54450	
106	H(1)	0.00048	2.12893	0.75966	0.71014	
107	H(1)	-0.00084	-3.73699	-1.33345	-1.24652	
108	H(1)	-0.00084	-3.73892	-1.33414	-1.24717	
109	H(1)	0.00048	2.13067	0.76028	0.71072	
110	H(1)	-0.00104	-4.63325	-1.65326	-1.54549	
111	H(1)	0.00060	2.67710	0.95526	0.89299	

g value:

$g_{xx} = 2.0023193 + 80.9 \cdot 10^{-6}$
$g_{yy} = 2.0023193 + 292.8 \cdot 10^{-6}$
$g_{zz} = 2.0023193 + 671.8 \cdot 10^{-6}$
$g_{iso} = (g_{xx} + g_{yy} + g_{zz}) / 3 = 2.0026$

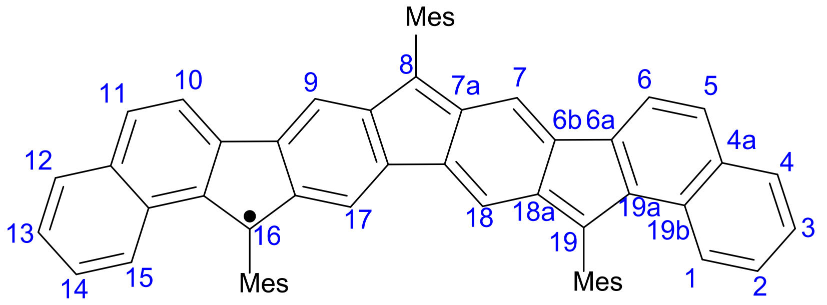

Isotropic Fermi Contact Couplings

	Atom	a.u.	MegaHertz	Gauss	10(-4) cm-1	IUPAC numbering
1	C(13)	-0.02223	-24.99093	-8.91739	-8.33608	
2	C(13)	0.00535	6.01602	2.14666	2.00673	C-6b
3	C(13)	-0.00945	-10.62737	-3.79211	-3.54491	C-7
4	C(13)	0.01755	19.73190	7.04083	6.58185	C-7a
5	C(13)	-0.00926	-10.40665	-3.71335	-3.47129	
6	C(13)	0.01227	13.79528	4.92250	4.60161	C-18
7	H(1)	0.00109	4.86006	1.73419	1.62114	
8	H(1)	-0.00197	-8.79646	-3.13880	-2.93418	
9	C(13)	0.01755	19.73313	7.04127	6.58226	
10	C(13)	-0.00945	-10.62922	-3.79277	-3.54553	C-9
11	C(13)	0.00535	6.01564	2.14653	2.00660	
12	C(13)	-0.02223	-24.99051	-8.91723	-8.33594	
13	C(13)	0.01227	13.79287	4.92164	4.60081	C-17
14	C(13)	-0.00926	-10.40501	-3.71277	-3.47074	
15	H(1)	0.00109	4.86264	1.73511	1.62200	
16	H(1)	-0.00197	-8.79509	-3.13831	-2.93373	
17	C(13)	0.01065	11.97254	4.27210	3.99361	

18	C(13)	-0.02139	-24.04439	-8.57964	-8.02034	
19	C(13)	0.03152	**35.43531**	12.64420	11.81995	C-16
20	C(13)	0.01065	11.96977	4.27111	3.99269	C-6a
21	C(13)	0.03152	**35.43283**	12.64332	11.81912	C-19
22	C(13)	-0.02139	-24.04085	-8.57837	-8.01916	C-19a
23	C(13)	-0.01221	-13.73023	-4.89929	-4.57991	C-10
24	C(13)	0.00824	9.26058	3.30441	3.08900	
25	C(13)	0.01222	13.73986	4.90272	4.58312	C-11
26	C(13)	-0.00992	-11.15592	-3.98071	-3.72121	
27	C(13)	-0.01221	-13.72822	-4.89857	-4.57924	C-6
28	C(13)	0.00824	9.25804	3.30350	3.08815	C-19b
29	C(13)	0.01222	13.73608	4.90138	4.58186	C-5
30	C(13)	-0.00992	-11.15255	-3.97951	-3.72009	
31	C(13)	-0.02399	**-26.97046**	-9.62373	-8.99638	C-8
32	C(13)	0.00933	10.48919	3.74280	3.49882	
33	C(13)	-0.01030	-11.58388	-4.13342	-3.86397	
34	C(13)	-0.01029	-11.56590	-4.12700	-3.85797	
35	C(13)	-0.00020	-0.22957	-0.08192	-0.07658	
36	C(13)	-0.00022	-0.24323	-0.08679	-0.08113	
37	C(13)	-0.00057	-0.64004	-0.22838	-0.21349	
38	H(1)	-0.00030	-1.33574	-0.47663	-0.44556	
39	H(1)	-0.00030	-1.32993	-0.47455	-0.44362	
40	C(13)	-0.01355	-15.23812	-5.43734	-5.08289	
41	C(13)	0.01536	17.26740	6.16144	5.75978	
42	C(13)	0.01561	17.54520	6.26056	5.85245	
43	C(13)	0.00071	0.80321	0.28660	0.26792	
44	C(13)	0.00069	0.77152	0.27530	0.25735	
45	C(13)	0.00045	0.51047	0.18215	0.17027	
46	H(1)	0.00043	1.92164	0.68569	0.64099	
47	H(1)	0.00044	1.95788	0.69862	0.65308	
48	C(13)	-0.01355	-15.23562	-5.43645	-5.08205	
49	C(13)	0.01536	17.26764	6.16153	5.75987	
50	C(13)	0.01560	17.54282	6.25972	5.85166	
51	C(13)	0.00072	0.80507	0.28727	0.26854	
52	C(13)	0.00069	0.77356	0.27602	0.25803	
53	C(13)	0.00045	0.50842	0.18142	0.16959	
54	H(1)	0.00043	1.92095	0.68544	0.64076	
55	H(1)	0.00044	1.95746	0.69847	0.65294	
56	C(13)	0.00037	0.41173	0.14692	0.13734	
57	H(1)	-0.00007	-0.32263	-0.11512	-0.10762	
58	H(1)	-0.00017	-0.76768	-0.27393	-0.25607	
59	H(1)	-0.00006	-0.24789	-0.08845	-0.08269	
60	C(13)	0.00038	0.43006	0.15346	0.14345	
61	H(1)	-0.00007	-0.31730	-0.11322	-0.10584	
62	H(1)	-0.00017	-0.76788	-0.27400	-0.25614	
63	H(1)	-0.00005	-0.24181	-0.08629	-0.08066	
64	C(13)	0.00027	0.30757	0.10975	0.10259	
65	H(1)	-0.00022	-0.96446	-0.34415	-0.32171	
66	H(1)	-0.00008	-0.35342	-0.12611	-0.11789	
67	H(1)	-0.00004	-0.17814	-0.06357	-0.05942	
68	C(13)	-0.00025	-0.27985	-0.09986	-0.09335	
69	H(1)	0.00018	0.79020	0.28196	0.26358	
70	H(1)	0.00008	0.37380	0.13338	0.12469	
71	H(1)	0.00002	0.11049	0.03943	0.03686	
72	C(13	-0.00023	-0.26100	-0.09313	-0.08706	
73	H(1)	0.00012	0.54885	0.19584	0.18308	
74	H(1)	0.00025	1.13851	0.40625	0.37976	
75	H(1)	0.00012	0.53861	0.19219	0.17966	
76	C(13)	0.00010	0.11323	0.04040	0.03777	

77	H(1)	0.00010	0.42792	0.15269	0.14274	
78	H(1)	0.00030	1.35028	0.48181	0.45041	
79	H(1)	0.00015	0.69160	0.24678	0.23069	
80	C(13)	0.00010	0.11446	0.04084	0.03818	
81	H(1)	0.00010	0.42865	0.15295	0.14298	
82	H(1)	0.00030	1.34778	0.48092	0.44957	
83	H(1)	0.00015	0.69177	0.24684	0.23075	
84	C(1	-0.00023	-0.25935	-0.09254	-0.08651	
85	H(1)	0.00012	0.54937	0.19603	0.18325	
86	H(1)	0.00025	1.13617	0.40541	0.37899	
87	H(1)	0.00012	0.53898	0.19232	0.17979	
88	C(13)	-0.00025	-0.27901	-0.09956	-0.09307	
89	H(1)	0.00009	0.38055	0.13579	0.12694	
90	H(1)	0.00002	0.10568	0.03771	0.03525	
91	H(1)	0.00018	0.78370	0.27965	0.26142	
92	H(1)	-0.00260	-11.63115	-4.15029	-3.87974	
93	H(1)	0.00095	4.22798	1.50865	1.41030	
94	H(1)	0.00095	4.22899	1.50901	1.41064	
95	H(1)	-0.00260	-11.63448	-4.15147	-3.88084	
96	C(13)	0.00473	5.31417	1.89623	1.77262	C-4
97	C(13)	-0.00594	-6.67823	-2.38296	-2.22762	C-1
98	C(13)	-0.00594	-6.68000	-2.38359	-2.22821	C-15
99	C(13)	0.00473	5.31605	1.89690	1.77324	C-12
100	C(13)	-0.00491	-5.52529	-1.97156	-1.84304	C-3
101	C(13)	0.00527	5.92300	2.11347	1.97570	C-2
102	C(13)	0.00527	5.92471	2.11408	1.97627	C-14
103	C(13)	-0.00492	-5.52712	-1.97221	-1.84365	C-13
104	H(1)	-0.00077	-3.45656	-1.23339	-1.15298	
105	H(1)	0.00058	2.57611	0.91922	0.85930	
106	H(1)	-0.00090	-4.03855	-1.44105	-1.34711	
107	H(1)	0.00065	2.91437	1.03992	0.97213	
108	H(1)	0.00065	2.91506	1.04017	0.97236	
109	H(1)	-0.00090	-4.03969	-1.44146	-1.34750	
110	H(1)	0.00058	2.57705	0.91956	0.85961	
111	H(1)	-0.00077	-3.45771	-1.23380	-1.15337	

16. Triradical Character

Structure optimized at the uM06/6-31g(d,p) level:

Input:

```
%chk=naph2_uhfmix.chk
%mem=600MW
%nprocshared=16
#p uhf/def2tzvp guess=mix scf=(qc,tight) pop=no stable

Title

0 2
// Coordinates for the uPBE0/def2tzvp-optimized structure
```

Output (excerpt):

```
Natural Orbital Coefficients:
                        206       207       208       209       210
     Eigenvalues --    1.83921   1.78209   1.73120   1.70912   1.36210
                       211       212       213       214       215
     Eigenvalues --    1.00000   0.63790   0.29088   0.26880   0.21791
```

Triradical character *y* was calculated using *Yamaguchi's* scheme.

n_{HONO} = 1.36210; n_{LUNO} = 0.63790

$$T = \frac{n_{HONO} - n_{LUNO}}{2}$$

$$y = 1 - \frac{2T}{1 + T^2}$$

$$y = 0.36$$

Input:

```
%chk=naph3_uhfmix.chk
%mem=600MW
%nprocshared=16
#p uhf/def2tzvp guess=mix scf=(qc,tight) pop=no stable

Title

0 2
// Coordinates for the uPBE0/def2tzvp-optimized structure
```

Output (excerpt):

```
Natural Orbital Coefficients:
                        206       207       208       209       210
     Eigenvalues --    1.84151   1.76885   1.76283   1.70061   1.33994
                       211       212       213       214       215
     Eigenvalues --    1.00000   0.66006   0.29939   0.23717   0.23115
```

n_{HONO} = 1.33994; n_{LUNO} = 0.66006

$$T = \frac{n_{HONO} - n_{LUNO}}{2}$$

$$y = 1 - \frac{2T}{1 + T^2}$$

$$y = 0.39$$

17. NICS Values of Radical 24 (top) and Radical 25 (bottom)

uB3LYP/6-311(d,p)

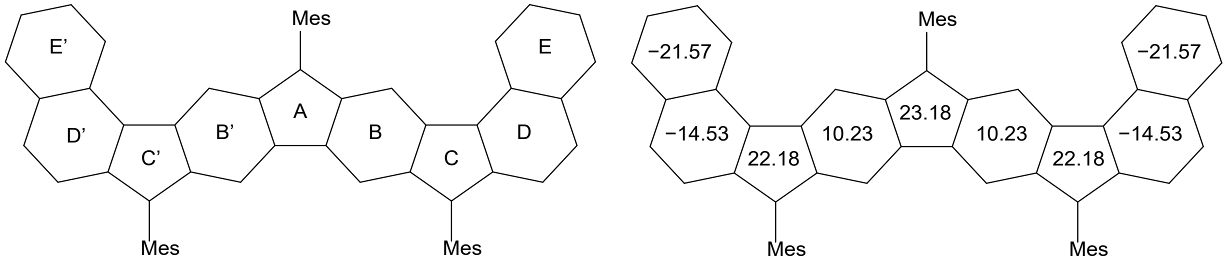

Rings E' & E

NICS@	isotropic	XX	YY	ZZ
0.0	-6.177200	-7.778000	-5.818000	-4.935700
0.1	-6.277300	-7.700500	-5.729100	-5.402200
0.2	-6.545400	-7.461000	-5.474400	-6.700900
0.3	-6.934800	-7.069400	-5.077000	-8.658000
0.4	-7.380900	-6.544900	-4.570500	-11.027300
0.5	-7.815900	-5.915800	-3.994100	-13.537800
0.6	-8.181300	-5.217800	-3.386400	-15.939700
0.7	-8.435900	-4.489600	-2.781100	-18.036900
0.8	-8.558200	-3.767800	-2.204000	-19.702900
0.9	-8.545100	-3.082700	-1.672600	-20.879900
**1.0**	**-8.406700**	**-2.455700**	**-1.196400**	**-21.568100**
1.1	-8.162300	-1.899300	-0.779200	-21.808500
1.2	-7.834900	-1.417900	-0.420500	-21.666400
1.3	-7.448400	-1.010400	-0.117400	-21.217400
1.4	-7.024600	-0.671800	0.134400	-20.536200
1.5	-6.582200	-0.394900	0.339500	-19.691200
1.6	-6.136400	-0.171600	0.503000	-18.740600
1.7	-5.698500	0.006300	0.629900	-17.731600
1.8	-5.276500	0.146600	0.725000	-16.701100
1.9	-4.875800	0.256100	0.793000	-15.676600
2.0	-4.499500	0.341000	0.838300	-14.677900
2.1	-4.149000	0.406400	0.865000	-13.718500
2.2	-3.824600	0.456200	0.876700	-12.806900
2.3	-3.525800	0.493900	0.876600	-11.947900
2.4	-3.251500	0.522000	0.867400	-11.143800
2.5	-3.000300	0.542200	0.851500	-10.394700
2.6	-2.770800	0.556100	0.830800	-9.699200
2.7	-2.561300	0.564900	0.806700	-9.055400
2.8	-2.370200	0.569200	0.780500	-8.460500
2.9	-2.196100	0.570000	0.753200	-7.911500
3.0	-2.037400	0.567800	0.725300	-7.405300
3.1	-1.892700	0.563000	0.697500	-6.938700
3.2	-1.760800	0.556300	0.670100	-6.508800
3.3	-1.640400	0.548000	0.643500	-6.112600
3.4	-1.530400	0.538400	0.617800	-5.747400
3.5	-1.429800	0.528000	0.593200	-5.410500
3.6	-1.337600	0.517100	0.569800	-5.099600
3.7	-1.253100	0.505800	0.547500	-4.812600
3.8	-1.175500	0.494400	0.526500	-4.547300
3.9	-1.104100	0.483000	0.506700	-4.302000
4.0	-1.038300	0.471900	0.488100	-4.074800
4.1	-0.977600	0.461000	0.470700	-3.864400
4.2	-0.921500	0.450500	0.454300	-3.669300

4.3	-0.869600	0.440500	0.439000	-3.488200
4.4	-0.821400	0.430800	0.424800	-3.319900
4.5	-0.776800	0.421700	0.411400	-3.163400
4.6	-0.735200	0.413000	0.399000	-3.017700
4.7	-0.696600	0.404800	0.387300	-2.881900
4.8	-0.660600	0.397000	0.376400	-2.755200
4.9	-0.627000	0.389700	0.366300	-2.636900
5.0	-0.595600	0.382700	0.356700	-2.526300
5.1	-0.595600	-0.711310	-0.434185	-0.000000

Rings D’ & D

NICS@	isotropic	XX	YY	ZZ
0.0	-3.927100	-8.363000	-6.861700	3.443400
0.1	-4.011500	-8.281800	-6.783300	3.030400
0.2	-4.259300	-8.011200	-6.545200	1.778700
0.3	-4.627800	-7.572300	-6.157200	-0.153900
0.4	-5.058200	-6.997600	-5.637300	-2.539800
0.5	-5.487900	-6.327700	-5.010700	-5.125400
0.6	-5.862000	-5.605700	-4.308600	-7.671800
0.7	-6.141100	-4.872700	-3.564600	-9.986100
0.8	-6.303600	-4.163000	-2.811300	-11.936600
0.9	-6.345000	-3.502300	-2.077900	-13.454800
**1.0**	**-6.273700**	**-2.906900**	**-1.387800**	**-14.526400**
1.1	-6.106700	-2.385000	-0.757900	-15.177200
1.2	-5.865400	-1.938500	-0.199100	-15.458600
1.3	-5.571600	-1.564500	0.283400	-15.433600
1.4	-5.245700	-1.257500	0.688400	-15.168000
1.5	-4.905100	-1.010400	1.018200	-14.722900
1.6	-4.563500	-0.815600	1.277300	-14.152200
1.7	-4.231200	-0.664900	1.472000	-13.500600
1.8	-3.915100	-0.550800	1.609400	-12.803800
1.9	-3.619300	-0.466100	1.697600	-12.089400
2.0	-3.346000	-0.404200	1.744400	-11.378100
2.1	-3.095500	-0.359700	1.757800	-10.684700
2.2	-2.867300	-0.327700	1.745300	-10.019500
2.3	-2.660000	-0.304200	1.713400	-9.389200
2.4	-2.471900	-0.286300	1.668100	-8.797600
2.5	-2.301400	-0.271500	1.614100	-8.246600
2.6	-2.146500	-0.258200	1.555200	-7.736500
2.7	-2.005700	-0.245000	1.494500	-7.266500
2.8	-1.877400	-0.231100	1.433900	-6.835000
2.9	-1.760300	-0.216100	1.375100	-6.439900
3.0	-1.653300	-0.199700	1.318700	-6.078800
3.1	-1.555200	-0.181600	1.265300	-5.749100
3.2	-1.465100	-0.162100	1.215100	-5.448300
3.3	-1.382300	-0.141200	1.167900	-5.173600
3.4	-1.306000	-0.119000	1.123700	-4.922600
3.5	-1.235500	-0.095800	1.082100	-4.692900
3.6	-1.170300	-0.071800	1.043000	-4.482300
3.7	-1.110000	-0.047300	1.006200	-4.288700
3.8	-1.053900	-0.022600	0.971200	-4.110400
3.9	-1.001800	0.002100	0.938100	-3.945500
4.0	-0.953200	0.026700	0.906500	-3.792700
4.1	-0.907800	0.050700	0.876400	-3.650500
4.2	-0.865300	0.074200	0.847600	-3.517900
4.3	-0.825600	0.096900	0.820100	-3.393600
4.4	-0.788200	0.118600	0.793800	-3.277000
4.5	-0.753100	0.139400	0.768500	-3.167100

4.6	-0.720000	0.159000	0.744400	-3.063200
4.7	-0.688700	0.177400	0.721200	-2.964800
4.8	-0.659300	0.194600	0.699100	-2.871400
4.9	-0.631400	0.210600	0.677800	-2.782500
5.0	-0.605000	0.225300	0.657500	-2.697700
5.1	-0.605000	-0.741813	0.657500	-0.000000

Rings C’ & C

NICS@	isotropic	XX	YY	ZZ
0.0	10.171300	-10.570000	-10.569600	51.653500
0.1	10.155800	-10.494800	-10.394800	51.357000
0.2	10.095600	-10.194000	-9.984700	50.465500
0.3	9.956700	-9.669600	-9.353800	48.893400
0.4	9.698700	-8.935700	-8.531400	46.563300
0.5	9.292500	-8.025700	-7.565000	43.468000
0.6	8.731500	-6.991600	-6.515900	39.702000
0.7	8.035100	-5.896700	-5.450500	35.452500
0.8	7.242600	-4.803100	-4.428600	30.959400
0.9	6.402800	-3.762900	-3.495300	26.466700
**1.0**	**5.563600**	**-2.812700**	**-2.678200**	**22.181800**
1.1	4.763700	-1.973300	-1.988700	18.253200
1.2	4.029300	-1.252600	-1.426100	14.766500
1.3	3.374000	-0.648800	-0.982000	11.752600
1.4	2.801100	-0.154600	-0.644300	9.202100
1.5	2.307100	0.240400	-0.399300	7.080100
1.6	1.884600	0.548200	-0.233300	5.338900
1.7	1.524600	0.780900	-0.133200	3.926200
1.8	1.218400	0.950600	-0.086500	2.791000
1.9	0.957900	1.068800	-0.081300	1.886200
2.0	0.736300	1.145900	-0.106800	1.170000
2.1	0.548200	1.191800	-0.152900	0.605900
2.2	0.389000	1.214800	-0.210500	0.162500
2.3	0.254700	1.222300	-0.271500	-0.186500
2.4	0.142300	1.219900	-0.329300	-0.463700
2.5	0.048800	1.212200	-0.378600	-0.687200
2.6	-0.028300	1.202400	-0.415500	-0.871800
2.7	-0.091400	1.192500	-0.437500	-1.029100
2.8	-0.142500	1.183700	-0.443500	-1.167800
2.9	-0.183600	1.176500	-0.433500	-1.293800
3.0	-0.216300	1.170700	-0.408400	-1.411300
3.1	-0.242200	1.166000	-0.370100	-1.522400
3.2	-0.262300	1.161800	-0.320700	-1.628100
3.3	-0.278000	1.157400	-0.262800	-1.728400
3.4	-0.289900	1.152300	-0.199000	-1.822800
3.5	-0.298800	1.146000	-0.131800	-1.910500
3.6	-0.305300	1.138100	-0.063300	-1.990600
3.7	-0.309800	1.128500	0.004500	-2.062300
3.8	-0.312600	1.117100	0.070200	-2.125100
3.9	-0.314100	1.103900	0.132400	-2.178700
4.0	-0.314400	1.089100	0.190500	-2.222900
4.1	-0.313800	1.072800	0.243800	-2.257800
4.2	-0.312200	1.055200	0.292100	-2.283900
4.3	-0.309900	1.036700	0.335300	-2.301600
4.4	-0.306800	1.017400	0.373500	-2.311400
4.5	-0.303200	0.997600	0.406900	-2.314100
4.6	-0.299100	0.977400	0.435700	-2.310300
4.7	-0.294500	0.957100	0.460200	-2.300700
4.8	-0.289500	0.936800	0.480900	-2.286100

4.9	-0.284100	0.916600	0.498000	-2.267000
5.0	-0.278500	0.896600	0.511900	-2.244000
5.1	-0.278500	-0.753186	-0.441637	-0.000000

Rings B’ & B

NICS@	isotropic	XX	YY	ZZ
0.0	5.188500	-9.661000	-6.595300	31.821900
0.1	5.144800	-9.546200	-6.523900	31.504600
0.2	5.013900	-9.214600	-6.284900	30.541200
0.3	4.797100	-8.693400	-5.887100	28.971800
0.4	4.499200	-8.023300	-5.348400	26.869200
0.5	4.130600	-7.252700	-4.696600	24.340900
0.6	3.708500	-6.430500	-3.967000	21.523000
0.7	3.255400	-5.601400	-3.199500	18.567000
0.8	2.795600	-4.801300	-2.432200	15.620500
0.9	2.352000	-4.056500	-1.698100	12.810600
**1.0**	**1.942500**	**-3.383300**	**-1.021500**	**10.232300**
1.1	1.578800	-2.790000	-0.417900	7.944100
1.2	1.266000	-2.279000	0.105600	5.971500
1.3	1.004100	-1.848400	0.547800	4.312800
1.4	0.789000	-1.493700	0.912500	2.948400
1.5	0.614800	-1.208900	1.205900	1.847500
1.6	0.474500	-0.987100	1.435900	0.974800
1.7	0.361700	-0.820700	1.611100	0.294700
1.8	0.270500	-0.702100	1.740000	-0.226300
1.9	0.196400	-0.623500	1.830700	-0.618100
2.0	0.135500	-0.577200	1.890400	-0.906700
2.1	0.085200	-0.555900	1.925800	-1.114100
2.2	0.043400	-0.553100	1.942200	-1.259000
2.3	0.008400	-0.562600	1.944200	-1.356500
2.4	-0.020900	-0.579200	1.935600	-1.419200
2.5	-0.045600	-0.598900	1.919100	-1.457000
2.6	-0.066400	-0.618000	1.896800	-1.477900
2.7	-0.084000	-0.634200	1.870300	-1.488000
2.8	-0.098900	-0.645700	1.840900	-1.492000
2.9	-0.111800	-0.651300	1.809200	-1.493300
3.0	-0.122900	-0.650400	1.776000	-1.494400
3.1	-0.132700	-0.642900	1.741600	-1.496700
3.2	-0.141200	-0.628900	1.706300	-1.501100
3.3	-0.148900	-0.608900	1.670400	-1.508200
3.4	-0.155800	-0.583500	1.634100	-1.517900
3.5	-0.161900	-0.553300	1.597500	-1.530100
3.6	-0.167500	-0.519000	1.560800	-1.544300
3.7	-0.172500	-0.481400	1.524100	-1.560300
3.8	-0.177100	-0.441300	1.487400	-1.577300
3.9	-0.181100	-0.399300	1.451000	-1.595000
4.0	-0.184600	-0.356000	1.414800	-1.612700
4.1	-0.187800	-0.312200	1.379100	-1.630200
4.2	-0.190400	-0.268200	1.343700	-1.646800
4.3	-0.192600	-0.224500	1.308900	-1.662300
4.4	-0.194400	-0.181600	1.274800	-1.676500
4.5	-0.195800	-0.139700	1.241200	-1.688900
4.6	-0.196800	-0.099100	1.208400	-1.699600
4.7	-0.197400	-0.060100	1.176300	-1.708300
4.8	-0.197600	-0.022700	1.145000	-1.715000
4.9	-0.197500	0.012800	1.114500	-1.719700
5.0	-0.197000	0.046400	1.084800	-1.722200
5.1	-0.197000	-0.558070	-0.625598	-0.308472

Ring A

NICS@	isotropic	XX	YY	ZZ
0.0	10.695800	-10.562300	-10.378100	53.027800
0.1	10.661100	-10.393600	-10.338200	52.715200
0.2	10.570400	-9.990400	-10.082400	51.783900
0.3	10.393600	-9.360700	-9.608300	50.149800
0.4	10.096200	-8.527800	-8.926200	47.742700
0.5	9.655100	-7.534200	-8.067200	44.566800
0.6	9.068900	-6.439400	-7.083600	40.729800
0.7	8.359700	-5.311200	-6.039900	36.430200
0.8	7.567200	-4.213200	-5.000500	31.915100
0.9	6.738400	-3.196100	-4.018900	27.430300
**1.0**	**5.918200**	**-2.293300**	**-3.132100**	**23.180100**
1.1	5.141900	-1.520900	-2.360100	19.306600
1.2	4.432300	-0.881500	-1.709000	15.887400
1.3	3.800500	-0.368700	-1.175300	12.945600
1.4	3.248200	0.029100	-0.749700	10.465200
1.5	2.770900	0.326200	-0.420100	8.406600
1.6	2.361000	0.537300	-0.173400	6.719000
1.7	2.009700	0.676900	0.003400	5.348700
1.8	1.708700	0.758800	0.123100	4.244100
1.9	1.450600	0.795600	0.197400	3.358700
2.0	1.229300	0.799000	0.237200	2.651500
2.1	1.039500	0.779200	0.252100	2.087200
2.2	0.877100	0.745100	0.250600	1.635700
2.3	0.738300	0.703900	0.239700	1.271300
2.4	0.619900	0.661600	0.225200	0.973000
2.5	0.519000	0.622800	0.211300	0.723000
2.6	0.433000	0.590400	0.200900	0.507500
2.7	0.359300	0.566500	0.196100	0.315400
2.8	0.296100	0.552000	0.197600	0.138700
2.9	0.241300	0.546600	0.205600	-0.028200
3.0	0.193600	0.549800	0.219600	-0.188800
3.1	0.151500	0.560400	0.238800	-0.344700
3.2	0.114200	0.576800	0.262200	-0.496500
3.3	0.080800	0.597500	0.288500	-0.643600
3.4	0.050800	0.620800	0.316900	-0.785100
3.5	0.023800	0.645200	0.346100	-0.919900
3.6	-0.000500	0.669600	0.375500	-1.046700
3.7	-0.022400	0.692900	0.404300	-1.164400
3.8	-0.042000	0.714300	0.431900	-1.272200
3.9	-0.059500	0.733300	0.458000	-1.369800
4.0	-0.074900	0.749700	0.482300	-1.456700
4.1	-0.088400	0.763200	0.504700	-1.533200
4.2	-0.100200	0.773800	0.525000	-1.599400
4.3	-0.110300	0.781700	0.543300	-1.655800
4.4	-0.118900	0.786900	0.559600	-1.703100
4.5	-0.126000	0.789800	0.573800	-1.741700
4.6	-0.132000	0.790500	0.586200	-1.772600
4.7	-0.136800	0.789100	0.596800	-1.796300
4.8	-0.140600	0.786100	0.605700	-1.813600
4.9	-0.143500	0.781500	0.613000	-1.825100
5.0	-0.145700	0.775600	0.618800	-1.831600
5.1	-0.145700	-0.523323	-0.650054	-0.310848

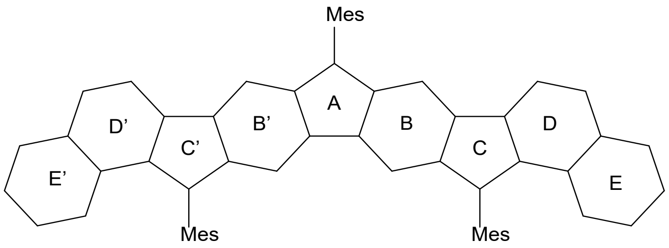

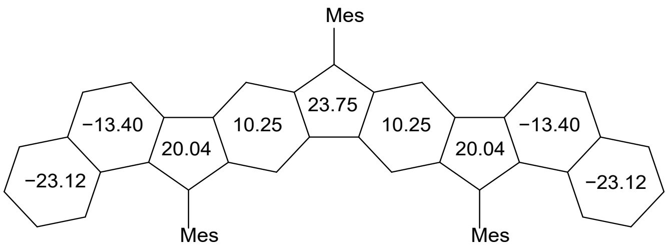

Rings E' & E

NICS@	isotropic	XX	YY	ZZ
0.0	-6.726500	-8.250100	-5.329100	-6.600300
0.1	-6.836600	-8.178700	-5.241900	-7.089200
0.2	-7.124900	-7.958300	-4.998300	-8.418200
0.3	-7.542000	-7.597200	-4.622100	-10.406600
0.4	-8.019200	-7.111700	-4.146600	-12.799400
0.5	-8.484300	-6.525800	-3.609200	-15.318000
0.6	-8.874800	-5.870400	-3.045700	-17.708300
0.7	-9.146500	-5.179000	-2.487000	-19.773400
0.8	-9.276300	-4.484200	-1.955900	-21.388900
0.9	-9.260600	-3.813400	-1.467300	-22.501200
**1.0**	**-9.110400**	**-3.187000**	**-1.029100**	**-23.115100**
1.1	-8.846000	-2.618200	-0.643700	-23.276200
1.2	-8.492300	-2.113400	-0.310300	-23.053300
1.3	-8.074800	-1.674100	-0.026000	-22.524200
1.4	-7.616900	-1.298100	0.212900	-21.765400
1.5	-7.139000	-0.981200	0.410400	-20.846100
1.6	-6.657300	-0.717700	0.570600	-19.824900
1.7	-6.184300	-0.501400	0.697600	-18.749200
1.8	-5.728800	-0.325900	0.795300	-17.655800
1.9	-5.296500	-0.185200	0.867700	-16.572200
2.0	-4.891100	-0.073500	0.918500	-15.518200
2.1	-4.513900	0.014300	0.951100	-14.507200
2.2	-4.165500	0.082400	0.968700	-13.547700
2.3	-3.845200	0.134700	0.974300	-12.644600
2.4	-3.551800	0.174300	0.970200	-11.799900
2.5	-3.283800	0.203700	0.958700	-11.013800
2.6	-3.039400	0.224900	0.941500	-10.284700
2.7	-2.816900	0.239500	0.920200	-9.610400
2.8	-2.614400	0.249000	0.895900	-8.988100
2.9	-2.430100	0.254400	0.869800	-8.414500
3.0	-2.262400	0.256700	0.842400	-7.886200
3.1	-2.109600	0.256500	0.814600	-7.399800
3.2	-1.970300	0.254600	0.786600	-6.952200
3.3	-1.843200	0.251400	0.759000	-6.540000
3.4	-1.727000	0.247400	0.732100	-6.160400
3.5	-1.620600	0.242900	0.705900	-5.810600
3.6	-1.523000	0.238300	0.680700	-5.488000
3.7	-1.433300	0.233700	0.656500	-5.190200
3.8	-1.350800	0.229300	0.633400	-4.915000
3.9	-1.274600	0.225300	0.611400	-4.660600
4.0	-1.204300	0.221600	0.590500	-4.424900
4.1	-1.139100	0.218400	0.570700	-4.206500
4.2	-1.078700	0.215600	0.551900	-4.003800
4.3	-1.022700	0.213300	0.534100	-3.815400
4.4	-0.970500	0.211500	0.517300	-3.640200
4.5	-0.921900	0.210000	0.501300	-3.476900
4.6	-0.876600	0.208800	0.486100	-3.324600
4.7	-0.834200	0.208000	0.471700	-3.182400

4.8	-0.794600	0.207500	0.458000	-3.049400
4.9	-0.757500	0.207100	0.445000	-2.924800
5.0	-0.722800	0.207000	0.432600	-2.808000
5.1	-0.722800	-0.646181	0.432600	-0.000000
5.2	-0.722800	-0.200277	0.432600	-0.000000

Rings D’ & D

NICS@	isotropic	XX	YY	ZZ
0.0	-3.378000	-6.754300	-8.574800	5.195200
0.1	-3.468100	-6.676100	-8.460400	4.732000
0.2	-3.721800	-6.425700	-8.176900	3.437100
0.3	-4.098900	-6.022400	-7.739700	1.465500
0.4	-4.542900	-5.495800	-7.172500	-0.960300
0.5	-4.993300	-4.881400	-6.506700	-3.591900
0.6	-5.396700	-4.216500	-5.778900	-6.194600
0.7	-5.713500	-3.536200	-5.026500	-8.577900
0.8	-5.921300	-2.869300	-4.283600	-10.610800
0.9	-6.013200	-2.238100	-3.577600	-12.223800
**1.0**	**-5.995100**	**-1.657100**	**-2.927900**	**-13.400400**
1.1	-5.881400	-1.134700	-2.345800	-14.163700
1.2	-5.690800	-0.674500	-1.836000	-14.561900
1.3	-5.443300	-0.276300	-1.398200	-14.655200
1.4	-5.157800	0.062200	-1.029000	-14.506700
1.5	-4.851100	0.345000	-0.723000	-14.175300
1.6	-4.536700	0.577000	-0.473900	-13.713100
1.7	-4.224900	0.763300	-0.274800	-13.163400
1.8	-3.923400	0.909600	-0.119000	-12.560800
1.9	-3.637000	1.021200	0.000100	-11.932200
2.0	-3.368500	1.103400	0.088800	-11.297700
2.1	-3.119500	1.160900	0.152600	-10.671900
2.2	-2.890000	1.198300	0.196600	-10.064900
2.3	-2.679600	1.219400	0.225200	-9.483500
2.4	-2.487400	1.227500	0.242100	-8.931700
2.5	-2.311900	1.225400	0.250500	-8.411800
2.6	-2.152000	1.215700	0.252800	-7.924500
2.7	-2.006300	1.200000	0.250900	-7.469700
2.8	-1.873300	1.180100	0.246400	-7.046600
2.9	-1.752000	1.157200	0.240500	-6.653700
3.0	-1.641200	1.132200	0.233900	-6.289600
3.1	-1.539700	1.105900	0.227400	-5.952500
3.2	-1.446800	1.078900	0.221300	-5.640500
3.3	-1.361400	1.051800	0.215900	-5.351900
3.4	-1.282900	1.024700	0.211400	-5.084900
3.5	-1.210600	0.998100	0.207900	-4.837800
3.6	-1.143800	0.972100	0.205400	-4.608900
3.7	-1.082100	0.946800	0.203900	-4.396800
3.8	-1.024800	0.922300	0.203300	-4.200000
3.9	-0.971700	0.898600	0.203500	-4.017200
4.0	-0.922300	0.875800	0.204400	-3.847100
4.1	-0.876300	0.853900	0.205900	-3.688600
4.2	-0.833300	0.832800	0.208000	-3.540800
4.3	-0.793200	0.812500	0.210400	-3.402600
4.4	-0.755700	0.793000	0.213200	-3.273200
4.5	-0.720500	0.774300	0.216100	-3.151900
4.6	-0.687500	0.756300	0.219200	-3.037800
4.7	-0.656400	0.739000	0.222300	-2.930500
4.8	-0.627200	0.722200	0.225400	-2.829300
4.9	-0.599700	0.706100	0.228400	-2.733700

5.0	-0.573800	0.690600	0.231300	-2.643300
5.1	-0.573800	-0.348269	-0.759087	-0.000000

Rings C’ & C

NICS@	isotropic	XX	YY	ZZ
0.0	9.542600	-10.579400	-10.110000	49.317300
0.1	9.523000	-10.484300	-9.969800	49.023200
0.2	9.437800	-10.185500	-9.586600	48.085500
0.3	9.260500	-9.676500	-8.980300	46.438200
0.4	8.961000	-8.960800	-8.187100	44.031000
0.5	8.520300	-8.062000	-7.260400	40.883300
0.6	7.939600	-7.025400	-6.265100	37.109300
0.7	7.242400	-5.910300	-5.267400	32.904800
0.8	6.468400	-4.778900	-4.324500	28.508600
0.9	5.664300	-3.685800	-3.477400	24.156100
**1.0**	**4.873900**	**-2.671500**	**-2.749000**	**20.042100**
1.1	4.131400	-1.761500	-2.146500	16.302100
1.2	3.458500	-0.967900	-1.666500	13.009900
1.3	2.865300	-0.292500	-1.298800	10.187100
1.4	2.352200	0.269300	-1.030700	7.817900
1.5	1.913800	0.726200	-0.848300	5.863500
1.6	1.541600	1.089000	-0.738200	4.274000
1.7	1.226200	1.369500	-0.687500	2.996600
1.8	0.958600	1.579400	-0.684200	1.980600
1.9	0.731300	1.730300	-0.716700	1.180100
2.0	0.537700	1.833000	-0.774000	0.554300
2.1	0.373000	1.897200	-0.846400	0.068100
2.2	0.232900	1.931700	-0.924500	-0.308300
2.3	0.114200	1.943700	-1.000700	-0.600300
2.4	0.014000	1.939300	-1.068300	-0.828900
2.5	-0.070200	1.923200	-1.122400	-1.011400
2.6	-0.140700	1.899000	-1.159300	-1.161600
2.7	-0.199300	1.869300	-1.177000	-1.290200
2.8	-0.248000	1.835900	-1.174800	-1.405000
2.9	-0.288100	1.800100	-1.153000	-1.511600
3.0	-0.321200	1.762800	-1.113100	-1.613300
3.1	-0.348200	1.724400	-1.057300	-1.711800
3.2	-0.370100	1.685300	-0.988200	-1.807600
3.3	-0.387700	1.645900	-0.908600	-1.900300
3.4	-0.401500	1.606200	-0.821500	-1.989100
3.5	-0.412000	1.566400	-0.729600	-2.072700
3.6	-0.419600	1.526700	-0.635500	-2.149900
3.7	-0.424600	1.487200	-0.541400	-2.219600
3.8	-0.427300	1.448000	-0.449000	-2.281000
3.9	-0.428100	1.409300	-0.359900	-2.333600
4.0	-0.427000	1.371100	-0.275200	-2.376900
4.1	-0.424400	1.333500	-0.195500	-2.411100
4.2	-0.420400	1.296600	-0.121400	-2.436200
4.3	-0.415200	1.260400	-0.053100	-2.452800
4.4	-0.409000	1.225000	0.009200	-2.461200
4.5	-0.401900	1.190500	0.065800	-2.462100
4.6	-0.394200	1.156800	0.116700	-2.456200
4.7	-0.386000	1.124100	0.162200	-2.444200
4.8	-0.377300	1.092200	0.202600	-2.426800
4.9	-0.368300	1.061300	0.238400	-2.404700
5.0	-0.359200	1.031400	0.269700	-2.378600
5.1	-0.359200	-0.109136	0.269700	-0.000000

Rings B’ & B

NICS@	isotropic	XX	YY	ZZ
0.0	5.396600	-9.618500	-6.276100	32.084300
0.1	5.358700	-9.529700	-6.193100	31.798900
0.2	5.226900	-9.230100	-5.942000	30.852900
0.3	5.004200	-8.743600	-5.530600	29.286800
0.4	4.696600	-8.107200	-4.976400	27.173400
0.5	4.316000	-7.365800	-4.306600	24.620200
0.6	3.880400	-6.566600	-3.556800	21.764600
0.7	3.413000	-5.753500	-2.767200	18.759600
0.8	2.938600	-4.963000	-1.977400	15.756200
0.9	2.480400	-4.222400	-1.221800	12.885600
**1.0**	**2.056700**	**-3.549800**	**-0.526800**	**10.246800**
1.1	1.679400	-2.955200	0.090300	7.902900
1.2	1.353600	-2.442300	0.620700	5.882400
1.3	1.079400	-2.010400	1.062300	4.186200
1.4	0.852700	-1.655900	1.418500	2.795600
1.5	0.667600	-1.373100	1.695900	1.680100
1.6	0.517300	-1.155500	1.903400	0.803900
1.7	0.395200	-0.995300	2.050800	0.130100
1.8	0.295700	-0.884600	2.147900	-0.376300
1.9	0.214100	-0.815100	2.204500	-0.747100
2.0	0.146800	-0.778700	2.229400	-1.010300
2.1	0.091100	-0.767300	2.230300	-1.189700
2.2	0.044800	-0.773700	2.213900	-1.305600
2.3	0.006400	-0.791400	2.185400	-1.374800
2.4	-0.025500	-0.814600	2.149100	-1.410900
2.5	-0.051900	-0.838800	2.108100	-1.425000
2.6	-0.073800	-0.860400	2.064600	-1.425700
2.7	-0.092000	-0.876600	2.020100	-1.419500
2.8	-0.107200	-0.885900	1.975500	-1.411200
2.9	-0.120000	-0.887000	1.931300	-1.404300
3.0	-0.130900	-0.879600	1.887800	-1.400800
3.1	-0.140300	-0.863800	1.845000	-1.402100
3.2	-0.148500	-0.839900	1.802800	-1.408600
3.3	-0.155900	-0.808600	1.761300	-1.420200
3.4	-0.162500	-0.770900	1.720200	-1.436700
3.5	-0.168400	-0.727600	1.679400	-1.457200
3.6	-0.173900	-0.679600	1.639000	-1.481000
3.7	-0.178900	-0.628100	1.598700	-1.507200
3.8	-0.183400	-0.573900	1.558700	-1.534900
3.9	-0.187400	-0.517800	1.518900	-1.563300
4.0	-0.191000	-0.460700	1.479200	-1.591600
4.1	-0.194200	-0.403400	1.439900	-1.619000
4.2	-0.196900	-0.346400	1.400800	-1.645100
4.3	-0.199100	-0.290200	1.362200	-1.669400
4.4	-0.200900	-0.235400	1.324000	-1.691400
4.5	-0.202300	-0.182300	1.286400	-1.711000
4.6	-0.203200	-0.131100	1.249400	-1.727900
4.7	-0.203700	-0.082200	1.213100	-1.741900
4.8	-0.203700	-0.035700	1.177600	-1.753100
4.9	-0.203400	0.008200	1.142900	-1.761400
5.0	-0.202700	0.049600	1.109100	-1.766900
5.1	-0.202700	-0.120185	1.109100	-0.000000

Ring A

```
NICS@ isotropic      XX       YY       ZZ
0.0 10.852000 -10.650600 -10.466800 53.673400
0.1 10.832000 -10.488100 -10.396700 53.380800
0.2 10.765500 -10.085100 -10.100800 52.482500
0.3 10.617400 -9.453400 -9.581100 50.886800
0.4 10.346700 -8.620100 -8.853200 48.513500
0.5 9.924500 -7.630500 -7.953200 45.357100
0.6 9.345600 -6.545000 -6.937100 41.518700
0.7 8.630800 -5.430400 -5.871300 37.194100
0.8 7.820900 -4.348600 -4.820400 32.631600
0.9 6.965700 -3.347800 -3.836400 28.081400
1.0 6.113300 -2.459300 -2.954500 23.753700
1.1 5.302300 -1.697800 -2.192100 19.796900
1.2 4.558400 -1.065300 -1.553500 16.294100
1.3 3.894700 -0.555500 -1.033100 13.272800
1.4 3.314200 -0.157000 -0.620600 10.720200
1.5 2.813000 0.143500 -0.302800 8.598300
1.6 2.383700 0.360000 -0.066000 6.857100
1.7 2.017400 0.506200 0.103100 5.442800
1.8 1.705300 0.595300 0.217200 4.303500
1.9 1.439700 0.639400 0.288000 3.391500
2.0 1.213600 0.649600 0.326200 2.665000
2.1 1.021500 0.636000 0.341100 2.087500
2.2 0.858500 0.607100 0.340700 1.627800
2.3 0.720500 0.570500 0.331800 1.259300
2.4 0.603800 0.532000 0.319700 0.959800
2.5 0.505200 0.496200 0.308300 0.711100
2.6 0.421700 0.466500 0.300300 0.498400
2.7 0.350800 0.444900 0.297300 0.310300
2.8 0.290300 0.432500 0.300000 0.138300
2.9 0.238100 0.429400 0.308500 -0.023600
3.0 0.192700 0.434900 0.322300 -0.179000
3.1 0.152900 0.448000 0.340500 -0.329800
3.2 0.117500 0.467100 0.362100 -0.476700
3.3 0.085900 0.490800 0.386000 -0.619200
3.4 0.057500 0.517400 0.411300 -0.756300
3.5 0.031800 0.545400 0.437100 -0.887000
3.6 0.008700 0.573600 0.462500 -1.009900
3.7 -0.012100 0.600800 0.486900 -1.124100
3.8 -0.030700 0.626400 0.510000 -1.228600
3.9 -0.047300 0.649600 0.531400 -1.323000
4.0 -0.062000 0.670300 0.550800 -1.407000
4.1 -0.074800 0.688100 0.568200 -1.480700
4.2 -0.085900 0.703100 0.583600 -1.544300
4.3 -0.095400 0.715300 0.596900 -1.598200
4.4 -0.103400 0.724800 0.608200 -1.643000
4.5 -0.110000 0.731900 0.617500 -1.679400
4.6 -0.115400 0.736700 0.625100 -1.708100
4.7 -0.119800 0.739400 0.631100 -1.729800
4.8 -0.123200 0.740300 0.635400 -1.745200
4.9 -0.125700 0.739500 0.638300 -1.754900
5.0 -0.127500 0.737300 0.639900 -1.759700
5.1 -0.127500 -0.965785 -0.102506 -0.000000
```

18. NICS-XY-scans of Radical 24 (top) and Radical 25 (bottom)

uB3LYP/6-311(d,p) nmr=giao integral=(grid=ultrafine) cphf=(grid=fine)

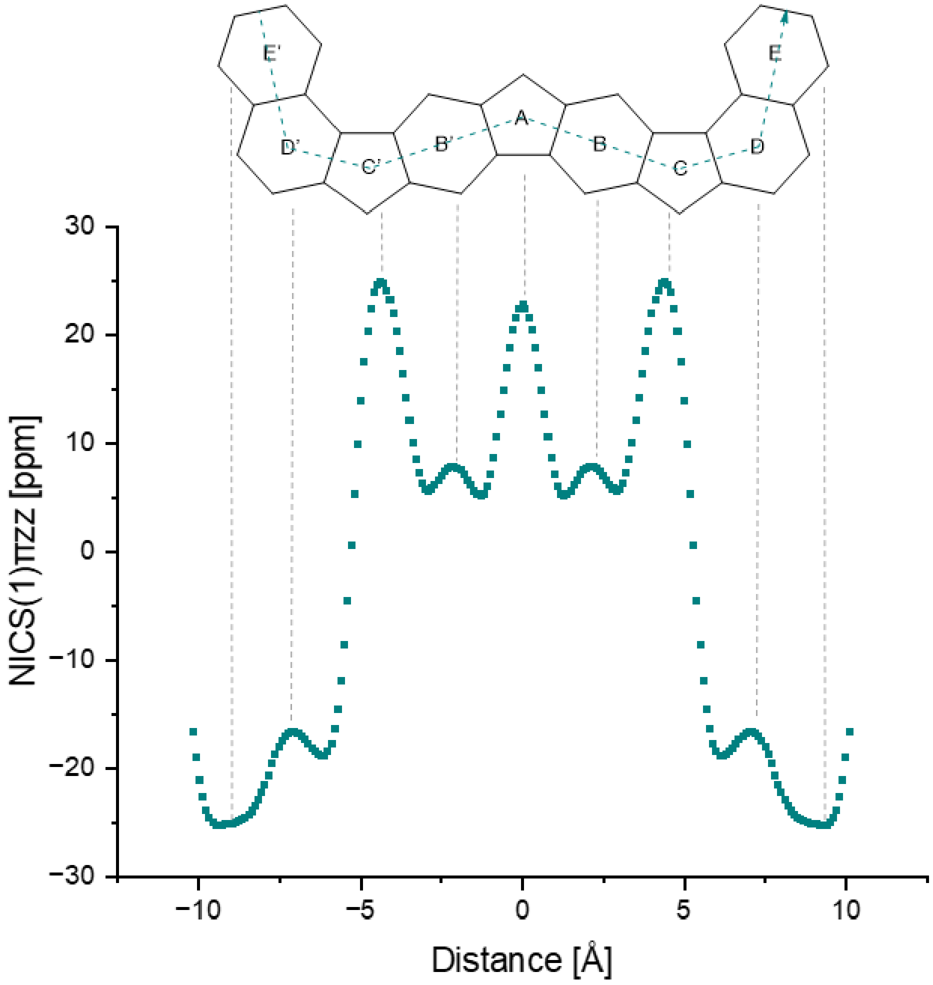

r	ZZ	Sigma-ZZ	Del-ZZ
r	ZZ	Sigma-ZZ	Del-ZZ
0.00	-27.8985	-11.2907	-16.6078
0.10	-29.6851	-10.7061	-18.9790
0.20	-30.5060	-9.5088	-20.9972
0.30	-30.4438	-7.8577	-22.5861
0.40	-29.6855	-5.9517	-23.7338
0.50	-28.4713	-3.9863	-24.4850
0.60	-27.0403	-2.1239	-24.9164
0.70	-25.5938	-0.4763	-25.1175
0.80	-24.2773	0.8948	-25.1721
0.90	-23.1810	1.9671	-25.1481
1.00	-22.3513	2.7437	-25.0950
1.10	-21.8055	3.2368	-25.0423
1.22	-21.5278	3.4675	-24.9953
1.32	-21.6033	3.3193	-24.9226
1.42	-21.9788	2.8434	-24.8222
1.52	-22.6596	2.0231	-24.6827
1.62	-23.6446	0.8436	-24.4882
1.72	-24.9155	-0.6946	-24.2209
1.82	-26.4241	-2.5589	-23.8652

1.92	-28.0790	-4.6702	-23.4088
2.02	-29.7380	-6.8927	-22.8453
2.12	-31.2145	-9.0376	-22.1769
2.22	-32.2983	-10.8812	-21.4171
2.32	-32.7945	-12.2020	-20.5925
2.45	-32.3626	-12.8620	-19.5006
2.55	-31.1762	-12.4878	-18.6884
2.65	-29.3510	-11.3847	-17.9663
2.75	-27.0821	-9.7047	-17.3774
2.85	-24.6082	-7.6598	-16.9484
2.95	-22.1619	-5.4751	-16.6868
3.05	-19.9339	-3.3503	-16.5836
3.15	-18.0531	-1.4348	-16.6183
3.25	-16.5879	0.1782	-16.7661
3.35	-15.5567	1.4456	-17.0023
3.45	-14.9457	2.3602	-17.3059
3.55	-14.7243	2.9339	-17.6582
3.68	-14.9625	3.1981	-18.1606
3.78	-15.4259	3.0341	-18.4600
3.88	-16.1841	2.5111	-18.6952
3.98	-17.1964	1.6183	-18.8147
4.08	-18.3976	0.3478	-18.7454
4.18	-19.6842	-1.2928	-18.3914
4.28	-20.9041	-3.2661	-17.6380
4.38	-21.8511	-5.4891	-16.3620
4.48	-22.2752	-7.8258	-14.4494
4.58	-21.9123	-10.0909	-11.8214
4.68	-20.5333	-12.0667	-8.4666
4.78	-18.0043	-13.5392	-4.4651
4.89	-13.7238	-14.3924	0.6686
4.99	-8.9755	-14.3230	5.3475
5.09	-3.6245	-13.5213	9.8968
5.19	1.9300	-12.1140	14.0440
5.29	7.2928	-10.2943	17.5871
5.39	12.1313	-8.2858	20.4171
5.49	16.2052	-6.3067	22.5119
5.59	19.3651	-4.5482	23.9133
5.69	21.5367	-3.1615	24.6982
5.79	22.6954	-2.2571	24.9525
5.89	22.8591	-1.9065	24.7656
5.99	22.0513	-2.1268	24.1781
6.09	20.3373	-2.9009	23.2382
6.19	17.8056	-4.1600	21.9656
6.29	14.5877	-5.7956	20.3833
6.39	10.8698	-7.6584	18.5282
6.49	6.9008	-9.5628	16.4636
6.59	2.9882	-11.2968	14.2850
6.69	-0.5252	-12.6447	12.1195
6.79	-3.3080	-13.4192	10.1112
6.88	-4.9111	-13.5304	8.6193
6.98	-5.7417	-12.9879	7.2462
7.08	-5.4540	-11.7674	6.3134
7.18	-4.1926	-10.0068	5.8142
7.28	-2.2085	-7.8987	5.6902
7.38	0.1990	-5.6520	5.8510
7.48	2.7384	-3.4566	6.1950
7.58	5.1657	-1.4609	6.6266
7.68	7.3009	0.2349	7.0660
7.78	9.0267	1.5750	7.4517
7.88	10.2764	2.5364	7.7400

7.98	11.0171	3.1150	7.9021
8.08	11.2363	3.3146	7.9217
8.18	10.9246	3.1306	7.7940
8.28	10.0998	2.5720	7.5278
8.38	8.7813	1.6389	7.1424
8.48	7.0093	0.3382	6.6711
8.58	4.8588	-1.3061	6.1649
8.68	2.4541	-3.2391	5.6932
8.78	-0.0187	-5.3640	5.3453
8.88	-2.3130	-7.5370	5.2240
8.98	-4.1401	-9.5731	5.4330
9.08	-5.2096	-11.2682	6.0586
9.18	-5.2865	-12.4315	7.1450
9.28	-4.2449	-12.9258	8.6809
9.38	-2.1172	-12.6979	10.5807
9.48	0.9190	-11.7901	12.7091
9.58	4.5713	-10.3333	14.9046
9.68	8.4939	-8.5164	17.0103
9.78	12.3456	-6.5496	18.8952
9.88	15.8331	-4.6332	20.4663
9.98	18.7299	-2.9362	21.6661
10.08	20.8757	-1.5873	22.4630
10.18	22.1646	-0.6729	22.8375
10.27	22.5414	-0.2644	22.8058
10.37	22.0674	-0.1711	22.2385
10.47	20.6829	-0.4849	21.1678
10.57	18.4533	-1.1225	19.5758
10.67	15.4895	-1.9689	17.4584
10.77	11.9584	-2.8822	14.8406
10.87	8.0933	-3.6990	11.7923
10.97	4.1927	-4.2470	8.4397
11.07	0.5990	-4.3629	4.9619
11.17	-2.3455	-3.9184	1.5729
11.27	-4.3591	-2.8448	-1.5143

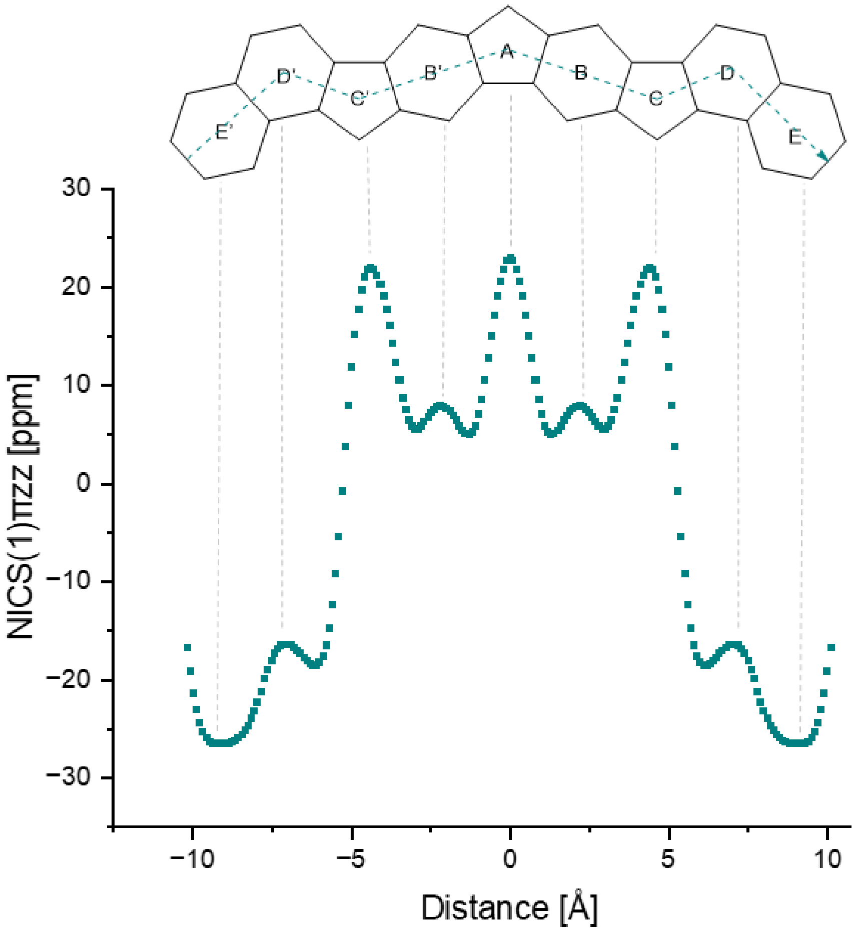

r	ZZ	Sigma-ZZ	Del-ZZ
0.00	-28.9980	-12.3546	-16.6434
0.10	-30.9467	-11.8064	-19.1403
0.20	-31.9026	-10.5893	-21.3133
0.30	-31.9437	-8.8693	-23.0744
0.40	-31.2572	-6.8565	-24.4007
0.50	-30.0873	-4.7623	-25.3250
0.60	-28.6797	-2.7639	-25.9158
0.70	-27.2433	-0.9869	-26.2564
0.80	-25.9312	0.4964	-26.4276
0.90	-24.8405	1.6567	-26.4972
1.00	-24.0225	2.4928	-26.5153
1.10	-23.4985	3.0159	-26.5144
1.22	-23.2638	3.2452	-26.5090
1.32	-23.3367	3.0838	-26.4205
1.42	-23.7118	2.5877	-26.2995
1.52	-24.3936	1.7391	-26.1327
1.62	-25.3784	0.5230	-25.9014
1.72	-26.6436	-1.0589	-25.5847
1.82	-28.1345	-2.9720	-25.1625
1.92	-29.7514	-5.1327	-24.6187
2.02	-31.3434	-7.4004	-23.9430
2.12	-32.7159	-9.5793	-23.1366
2.22	-33.6542	-11.4392	-22.2150
2.32	-33.9643	-12.7534	-21.2109
2.44	-33.2868	-13.3673	-19.9195
2.54	-31.8833	-12.9533	-18.9300

2.64	-29.8351	-11.7852	-18.0499
2.74	-27.3500	-10.0196	-17.3304
2.84	-24.6765	-7.8746	-16.8019
2.94	-22.0533	-5.5831	-16.4702
3.04	-19.6730	-3.3530	-16.3200
3.14	-17.6628	-1.3399	-16.3229
3.24	-16.0873	0.3585	-16.4458
3.34	-14.9602	1.6959	-16.6561
3.44	-14.2630	2.6640	-16.9270
3.54	-13.9618	3.2752	-17.2370
3.68	-14.1258	3.5665	-17.6923
3.78	-14.5909	3.4288	-18.0197
3.88	-15.3526	2.9449	-18.2975
3.98	-16.3719	2.1047	-18.4766
4.08	-17.5862	0.8997	-18.4859
4.18	-18.8963	-0.6643	-18.2320
4.28	-20.1553	-2.5528	-17.6025
4.38	-21.1634	-4.6870	-16.4764
4.48	-21.6774	-6.9359	-14.7415
4.58	-21.4388	-9.1181	-12.3207
4.68	-20.2222	-11.0204	-9.2018
4.78	-17.8933	-12.4323	-5.4610
4.89	-13.9334	-13.2308	-0.7026
4.99	-9.4512	-13.1438	3.6926
5.09	-4.3749	-12.3400	7.9651
5.19	0.9111	-10.9446	11.8557
5.29	6.0227	-9.1498	15.1725
5.39	10.6344	-7.1784	17.8128
5.49	14.5100	-5.2485	19.7585
5.59	17.5006	-3.5513	21.0519
5.69	19.5301	-2.2387	21.7688
5.79	20.5715	-1.4212	21.9927
5.89	20.6550	-1.1668	21.8218
5.99	19.8143	-1.4258	21.2401
6.09	18.0965	-2.2536	20.3501
6.19	15.5880	-3.5845	19.1725
6.29	12.4204	-5.3122	17.7326
6.39	8.7787	-7.2893	16.0680
6.49	4.9116	-9.3282	14.2398
6.59	1.1258	-11.2123	12.3381
6.69	-2.2380	-12.7181	10.4801
6.79	-4.8525	-13.6478	8.7953
6.87	-6.3184	-13.8811	7.5627
6.97	-6.9478	-13.4452	6.4974
7.07	-6.4629	-12.3016	5.8387
7.17	-5.0177	-10.5855	5.5678
7.27	-2.8713	-8.4906	5.6193
7.37	-0.3274	-6.2292	5.9018
7.47	2.3204	-3.9967	6.3171
7.57	4.8291	-1.9486	6.7777
7.67	7.0208	-0.1917	7.2125
7.77	8.7813	1.2116	7.5697
7.87	10.0467	2.2327	7.8140
7.97	10.7883	2.8638	7.9245
8.07	10.9981	3.1056	7.8925
8.17	10.6876	2.9717	7.7159
8.27	9.8569	2.4535	7.4034
8.37	8.5279	1.5517	6.9762
8.47	6.7433	0.2731	6.4702
8.57	4.5814	-1.3578	5.9392

8.67	2.1700	-3.2857	5.4557
8.77	-0.3011	-5.4123	5.1112
8.87	-2.5823	-7.5910	5.0087
8.97	-4.3818	-9.6341	5.2523
9.07	-5.4091	-11.3335	5.9244
9.17	-5.4308	-12.4965	7.0657
9.27	-4.3575	-12.9829	8.6254
9.37	-2.1833	-12.7595	10.5762
9.47	0.9012	-11.8527	12.7539
9.57	4.6006	-10.3941	14.9947
9.67	8.5666	-8.5729	17.1395
9.77	12.4562	-6.6006	19.0568
9.87	15.9754	-4.6780	20.6534
9.97	18.8972	-2.9755	21.8727
10.07	21.0619	-1.6230	22.6849
10.17	22.3640	-0.7090	23.0730
10.26	22.7476	-0.3046	23.0522
10.36	22.2662	-0.2232	22.4894
10.46	20.8684	-0.5584	21.4268
10.56	18.6199	-1.2284	19.8483
10.66	15.6304	-2.1202	17.7506
10.76	12.0671	-3.0919	15.1590
10.86	8.1637	-3.9798	12.1435
10.96	4.2192	-4.6091	8.8283
11.06	0.5781	-4.8131	5.3912
11.16	-2.4142	-4.4584	2.0442
11.26	-4.4740	-3.4714	-1.0026

19. ACID Plots of Radical 24 (top) and Radical 25 (bottom)(calculated without mesityl groups)

nmr=csgt upbe0/def2tzvp (GD3BJ) iop(10/93=1); isosurface values of 0.025

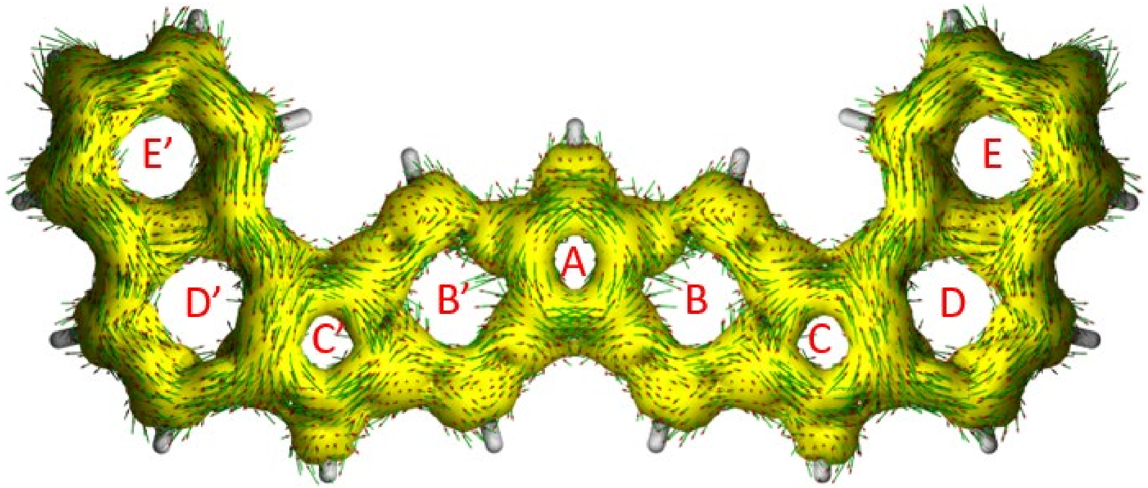

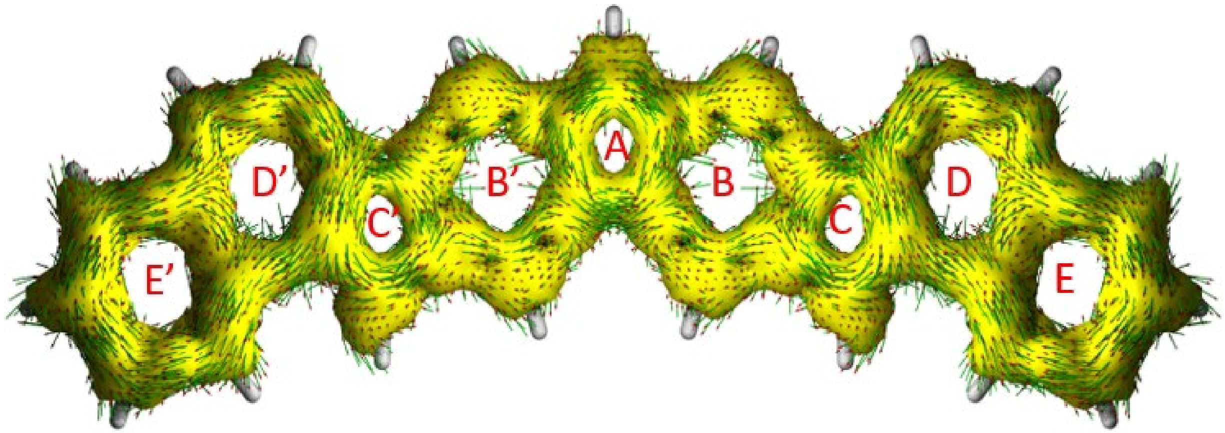

20. References

[20] X. Yang, D. Zhang, Y. Liao, D. Zhao, "Toward an Air-Stable Triradical with Strong Spin Coupling: Synthesis of Substituted Truxene-5,10,15-triyl" *J. Org. Chem.* **2020**, *85*, 5761-5770.

[53] J.-B. Bongui, A. Elomri, D. Cahard, F. Tillequin, B. Pfeiffer, A. Pierré, E. Seguin, "Synthesis and Cytotoxic Activity of Acronycine Analogues in the Benzo[*c*]pyrano[3,2-*h*]acridin-7-one and Naphtho[1,2-*b*][1,7] and [1,10]-Phenanthrolin-7(14*H*)-one Series" *Chem. Pharm. Bull.* **2005**, *53*, 1540-1546.

[54] S. Staniland, R. W. Adams, J. J. W. McDouall, I. Maffucci, A. Contini, D. M. Grainger, N. J. Turner, J. Clayden, "Biocatalytic Dynamic Kinetic Resolution for the Synthesis of Atropisomeric Biaryl N-Oxide Lewis Base Catalysts" *Angew. Chem. Int. Ed.* **2016**, *55*, 10755-10759.

[55] B. S. Kumar, K. Ravi, A. K. Verma, K. Fatima, M. Hasanain, A. Singh, J. Sarkar, S. Luqman, D. Chanda, A. S. Negi, "Synthesis of Pharmacologically Important Naphthoquinones and Anticancer Activity of 2-Benzyllawsone through DNA Topoisomerase-II Inhibition" *Bioorg. Med. Chem.* **2017**, *25*, 1364-1373.

[56] Z.-T. Du, J. Lu, H.-R. Yu, Y. Xu, A.-P. Li, "A Facile Demethylation of ortho Substituted Aryl Methyl Ethers Promoted by $AlCl_3$" *J. Chem. Res.* **2010**, *34*, 222-227.

[57] K. Urakawa, M. Sumimoto, M. Arisawa, M. Matsuda, H. Ishikawa, "Redox Switching of Orthoquinone-Containing Aromatic Compounds with Hydrogen and Oxygen Gas" *Angew. Chem. Int. Ed.* **2016**, *55*, 7432-7436.

[58] R. Savela, M. Majewski, R. Leino, "Iron-Catalyzed Arylation of Aromatic Ketones and Aldehydes Mediated by Organosilanes" *Eur. J. Org. Chem.* **2014**, 4137-4147.

[59] H. Sharma, P. K. Sharma, S. Das, "Revisiting Indeno[2,1-*c*]fluorene Synthesis while Exploring the Fully Conjugated *s*-Indaceno[2,1-*c*:6,5-*c'*]difluorene" *Chem. Commun.* **2020**, *56*, 11319-11322.

[66] Z. Chen, C. S. Wannere, C. Corminboeuf, R. Puchta, P. von R. Schleyer, "Nucleus-Independent Chemical Shifts (NICS) as an Aromaticity Criterion" *Chem. Rev.* **2005**, *105*, 3842-3888.

[71] D. Geuenich, K. Hess, F. Köhler, R. Herges, "Anisotropy of the Induced Current Density (ACID), a General Method To Quantify and Visualize Electronic Delocalization" *Chem. Rev.* **2005**, *105*, 3758-3772.

[76] G. R. Fulmer, A. J. M. Miller, N. H. Sherden, H. E. Gottlieb, A. Nudelman, B. M. Stoltz, J. E. Bercaw, K. I. Goldberg, "NMR Chemical Shifts of Trace Impurities: Common Laboratory Solvents, Organics, and Gases in Deuterated Solvents Relevant to the Organometallic Chemist" *Organometallics* **2010**, *29*, 2176-2179.

[77] L. Van Gerven, J. Talpe, A. Van Itterbeek, "Demagnetizing Effects in Paramagnetic Resonance. The *g*-Factor of DPPH" *Physica* **1967**, *33*, 207-211.

[78] S. Stoll, A. Schweiger, "EasySpin, a Comprehensive Software Package for Spectral Simulation and Analysis in EPR" *J. Magn. Reson.* **2006**, *178*, 42-55.

[79] J. Koziskova, F. Hahn, J. Richter, J. Kožíšek, " Comparison of Different Absorption Corrections on the Model Structure of Tetrakis(μ_2-acetato)-diaqua-di-copper(II)" *Acta Chim. Slovaca* **2016**, *9*, 136-140.

[80] O. V. Dolomanov, L. J. Bourhis, R. J. Gildea, J. A. K. Howard, H. Puschmann, "OLEX2: A Complete Structure Solution, Refinement and Analysis Program" *J. Appl. Crystallogr.* **2009**, *42*, 339-341.

[81] G. M. Sheldrick, "SHELXT - Integrated Space-Group and Crystal-Structure Determination" *Acta Crystallogr., Sect. A* **2015**, *71*, 3-8.

[82] G. M. Sheldrick, "Crystal Structure Refinement with SHELXL" *Acta Crystallogr., Sect. C: Struct. Chem.* **2015**, *71*, 3-8.

[83] J. P. Perdew, K. Burke, M. Ernzerhof, "Generalized Gradient Approximation Made Simple" *Phys. Rev. Lett.* **1996**, *77*, 3865-3868.

[84] J. P. Perdew, K. Burke, M. Ernzerhof, "Generalized Gradient Approximation Made Simple (Erratum)" *Phys. Rev. Lett.* **1997**, *78*, 1396.

[85] C. Adamo, V. Barone, "Toward Reliable Density Functional Methods without Adjustable Parameters: The PBE0 Model" *J. Chem. Phys.* **1999**, *110*, 6158-6170.

[86] F. Weigend, R. Ahlrichs, "Balanced Basis Sets of Split Valence, Triple Zeta Valence and Quadruple Zeta Valence Quality for H to Rn: Design and Assessment of Accuracy" *Phys. Chem. Chem. Phys.* **2005**, *7*, 3297-3305.

[87] F. Weigend, "Accurate Coulomb-Fitting Basis Sets for H to Rn" *Phys. Chem. Chem. Phys.* **2006**, *8*, 1057-1065.

[88] S. Grimme, J. Antony, S. Ehrlich, H. Krieg, "A Consistent and Accurate *ab initio* Parametrization of Density Functional Dispersion Correction (DFT-D) for the 94 Elements H-Pu" *J. Chem. Phys.* **2010**, *132*, 154104.

[89] S. Grimme, S. Ehrlich, L. Goerigk, "Effect of the Damping Function in Dispersion Corrected Density Functional Theory" *J. Comput. Chem.* **2011**, *32*, 1456-1465.

[90] Gaussian 16, Revision C.01, M. J. Frisch, G. W. Trucks, H. B. Schlegel, G. E. Scuseria, M. A. Robb, J. R. Cheeseman, G. Scalmani, V. Barone, G. A. Petersson, H. Nakatsuji, X. Li, M. Caricato, A. V. Marenich, J. Bloino, B. G. Janesko, R. Gomperts, B. Mennucci, H. P. Hratchian, J. V. Ortiz, A. F. Izmaylov, J. L. Sonnenberg, D. Williams-Young, F. Ding, F. Lipparini, F. Egidi, J. Goings, B. Peng, A. Petrone, T. Henderson, D. Ranasinghe, V. G. Zakrzewski, J. Gao, N. Rega, G. Zheng, W. Liang, M. Hada, M. Ehara, K. Toyota, R. Fukuda, J. Hasegawa, M. Ishida, T. Nakajima, Y. Honda, O. Kitao, H. Nakai, T. Vreven, K. Throssell, J. A. Montgomery, Jr., J. E. Peralta, F. Ogliaro, M. J. Bearpark, J. J. Heyd, E. N. Brothers, K. N. Kudin, V. N. Staroverov, T. A. Keith, R. Kobayashi, J. Normand, K. Raghavachari, A. P. Rendell, J. C. Burant, S. S. Iyengar, J. Tomasi, M. Cossi, J. M. Millam, M. Klene, C. Adamo, R. Cammi, J. W. Ochterski, R. L. Martin, K. Morokuma, O. Farkas, J. B. Foresman, D. J. Fox, Gaussian, Inc., Wallingford CT, **2019**.

[91] B. G. Johnson, M. J. Frisch, "Analytic Second Derivatives of the Gradient-Corrected Density Functional Energy. Effect of Quadrature Weight Derivatives" *Chem. Phys. Lett.* **1993**, *216*, 133–140.

[92] B. G. Johnson, M. J. Fisch, "An Implementation of Analytic Second Derivatives of the Gradient-Corrected Density Functional Energy" *J. Chem. Phys.* **1994**, *100*, 7429-7442.

[93] R. E. Stratmann, J. C. Burant, G. E. Scuseria, M. J. Frisch, "Improving Harmonic Vibrational Frequencies Calculations in Density Functional Theory" *J. Chem. Phys.* **1997**, *106*, 10175-10183.

[94] Y. Zhao, D. G. Truhlar, "The M06 Suite of Density Functionals for Main Group Thermochemistry, Thermochemical Kinetics, Noncovalent Interactions, Excited States, and Transition Elements: Two New Functionals and Systematic Testing of Four M06-Class Functionals and 12 Other Functionals" *Theor. Chem. Acc.* **2008**, *120*, 215-241.

[95] A. J. H. Wachters, "Gaussian Basis Set for Molecular Wavefunctions Containing Third-Row Atoms" *J. Chem. Phys.* **1970**, *52*, 1033-1036.

[96] P. J. Hay, "Gaussian Basis Sets for Molecular Calculations. The Representation of 3d Orbitals in Transition-Metal Atoms" *J. Chem. Phys.* **1977**, *66*, 4377-4384.

[97] A. D. McLean, G. S. Chandler, "Contracted Gaussian-Basis Sets for Molecular Calculations. 1. Second Row Atoms, *Z*=11-18" *J. Chem. Phys.* **1980**, *72*, 5639-5648.

[98] R. Krishnan, J. S. Binkley, R. Seeger, J. A. Pople, "Self-Consistent Molecular Orbital Methods. XX. A Basis Set for Correlated Wave Functions" *J. Chem. Phys.* **1980**, *72*, 650-654.

[99] T. Clark, J. Chandrasekhar, G. W. Spitznagel, P. von R. Schleyer, "Efficient Diffuse Function-Augmented Basis Sets for Anion Calculations. III. The 3-21+G Basis Set for First-Row Elements, Li–F" *J. Comput. Chem.* **1983**, *4*, 294-301.

[100] K. Raghavachari, G. W. Trucks, "Highly Correlated Systems. Excitation Energies of First Row Transition Metals Sc–Cu" *J. Chem. Phys.* **1989**, *91*, 1062-1065.

[101] N. M. O'Boyle, A. L. Tenderholt, K. M. Langner, "cclib: A Library for Package-independent Computational Chemistry Algorithms" *J. Comput. Chem.* **2008**, *29*, 839-845.

[102] M. P. Andersson, P. Uvdal, "New Scale Factors for Harmonic Vibrational Frequencies Using the B3LYP Density Functional Method with the Triple-ζ Basis Set 6-311+G(d,p)" *J. Phys. Chem. A* **2005**, *109*, 2937-2941.

[103] R. E. Stratmann, G. E. Scuseria, M. J. Frisch, "An Efficient Implementation of Time-Dependent Density-Functional Theory for the Calculation of Excitation Energies of Large Molecules" *J. Chem. Phys.* **1998**, *109*, 8218-8224.

[104] R. Bauernschmitt, R. Ahlrichs, "Treatment of Electronic Excitations within the Adiabatic Approximation of Time Dependent Density Fuctional Theory" *Chem. Phys. Lett.* **1996**, *256*, 454–464.

[105] M. E. Casida, C. Jamorski, K. C. Casida, D. R. Salahub, "Molecular Excitation Energies to High-Lying Bound States from Time-Dependent Density-Functional Response Theory: Characterization and Correction of the Time-Dependent Local Density Approximation Ionization Threshold" *J. Chem. Phys.* **1998**, *108*, 4439-4449.

[106] A. Klamt, G. Schüürmann, "COSMO: A New Approach to Dielectric Screening in Solvents with Explicit Expressions for the Screening Energy and its Gradient" *J. Chem. Soc., Perkin Trans. 2* **1993**, 799-805.

[107] V. Barone, M. Cossi, "Quantum Calculation of Molecular Energies and Energy Gradients in Solution by a Conductor Solvent Model" *J. Phys. Chem. A* **1998**, *102*, 1995-2001.

[108] M. Cossi, N. Rega, G. Scalmani, V. Barone, "Energies, Structures, and Electronic Properties of Molecules in Solution with the C-PCM Solvation Model" *J. Comput. Chem.* **2003**, *24*, 669-681.

[109] C. Lee, W. Yang, R. G. Parr, "Development of the Colle-Salvetti Correlation-Energy Formula into a Functional of the Electron Density" *Phys. Rev. B* **1988**, *37*, 785-789.

[110] W. Koch, M. C. Holthausen, *A Chemist's Guide to Density Functional Theory*, 2. ed., Wiley-VCH, Weinheim, **2002**.

[111] Y. Miyazawa, Z. Wang, M. Matsumoto, S. Hatano, I. Antol, E. Kayahara, S. Yamago, M. Abe, "1,3-Diradicals Embedded in Curved Paraphenylene Units: Singlet versus Triplet State and In-Plane Aromaticity" *J. Am. Chem. Soc.* **2021**, *143*, 7426-7439.

[112] Z. Wang, py.Aroma 4, https://wongzit.github.io/program/pyaroma (accessed data, 07/2025).

[113] F. London, "Théorie Quantique des Courants Interatomiques dans les Combinaisons Aromatiques" *J. Phys. Radium* **1937**, *8*, 397-409.

[114] R. McWeeny, "Perturbation Theory for the Fock-Dirac Density Matrix" *Phys. Rev.* **1962**, *126*, 1028-1034.

[115] K. Wolinski, J. F. Hinton, P. Pulay, "Efficient Implementation of the Gauge-Independent Atomic Orbital Method for NMR Chemical Shift Calculations" *J. Am. Chem. Soc.* **1990**, *112*, 8251-8260.

[116] J. R. Cheeseman, G. W. Trucks, T. A. Keith, M. J. Frisch, "A Comparison of Models for Calculating Nuclear Magnetic Resonance Shielding Tensors" *J. Chem. Phys.* **1996**, *104*, 5497-5509.

[117] V. Barone, " Electronic, Vibrational and Environmental Effects on the Hyperfine Coupling Constants of Nitroside Radicals. H_2NO as a Case Study" *Chem. Phys. Lett.* **1996**, *262*, 201-206.

[118] N. Rega, M. Cossi, V. Barone, "Development and Validation of Reliable Quantum Mechanical Approaches for the Study of Free Radicals in Solution" *J. Chem. Phys.* **1996**, *105*, 11060-11067.

[119] V. Barone, in *Recent Advances in Density Functional Methods. Part 1* (Ed.: D. P. Chong), World Scientific Publishing, Singapore, London, **1995**, pp. 287-334.

[120] GaussView (Version 6.1.1), R. Dennington, T. Keith, J. Millam, Semichem Inc., Shawnee Mission, KS, **2019**.

[121] Persistence of Vision (Version 3.6) Persistence of Vision Pty. Ltd., Williamstown, Australia **2004**, http://www.povray.org (accessed data, 07/2025).
